# Treatment of hyperprolactinemia: a systematic review and meta-analysis

**DOI:** 10.1186/2046-4053-1-33

**Published:** 2012-07-24

**Authors:** Amy T Wang, Rebecca J Mullan, Melanie A Lane, Ahmad Hazem, Chaithra Prasad, Nicola W Gathaiya, M Mercè Fernández-Balsells, Amy Bagatto, Fernando Coto-Yglesias, Jantey Carey, Tarig A Elraiyah, Patricia J Erwin, Gunjan Y Gandhi, Victor M Montori, Mohammad Hassan Murad

**Affiliations:** 1 Knowledge and Evaluation Research Unit, Mayo Clinic, 200 First Street SW, Rochester, MN, 55905, USA; 2 Division of General Internal Medicine, Mayo Clinic, 200 First Street SW, Rochester, MN, 55905, USA; 3 Division of Preventive Medicine, Mayo Clinic, 200 First Street SW, Rochester, MN, 55905, USA; 4 Division of Endocrinology, Diabetes, Metabolism, Nutrition, Mayo Clinic, 200 First Street SW, Rochester, MN, 55905, USA; 5 Endocrinology, Diabetes and Nutrition Unit, Hospital Universitari de Girona, Dr. Josep Trueta, Avinguda de França, Girona, 17007, Spain; 6 Hospital Nacional de Geriatría y Gerontología, Caja Costarricense de Seguro Social, Avenue 8, San José, Costa Rica; 7 Division of Endocrinology and Metabolism, Mayo Clinic, 4500 San Pablo Road, Jacksonville, FL, 32224, USA; 8 Mayo Clinic Libraries, Mayo Clinic, 200 First Street SW, Rochester, MN, 55905, USA

**Keywords:** Treatment, Hyperprolactinemia, Macroprolactinoma, Microprolactinoma

## Abstract

**Background:**

Hyperprolactinemia is a common endocrine disorder that can be associated with significant morbidity. We conducted a systematic review and meta-analyses of outcomes of hyperprolactinemic patients, including microadenomas and macroadenomas, to provide evidence-based recommendations for practitioners. Through this review, we aimed to compare efficacy and adverse effects of medications, surgery and radiotherapy in the treatment of hyperprolactinemia.

**Methods:**

We searched electronic databases, reviewed bibliographies of included articles, and contacted experts in the field. Eligible studies provided longitudinal follow-up of patients with hyperprolactinemia and evaluated outcomes of interest. We collected descriptive, quality and outcome data (tumor growth, visual field defects, infertility, sexual dysfunction, amenorrhea/oligomenorrhea and prolactin levels).

**Results:**

After review, 8 randomized and 178 nonrandomized studies (over 3,000 patients) met inclusion criteria. Compared to no treatment, dopamine agonists significantly reduced prolactin level (weighted mean difference, -45; 95% confidence interval, -77 to −11) and the likelihood of persistent hyperprolactinemia (relative risk, 0.90; 95% confidence interval, 0.81 to 0.99). Cabergoline was more effective than bromocriptine in reducing persistent hyperprolactinemia, amenorrhea/oligomenorrhea, and galactorrhea. A large body of noncomparative literature showed dopamine agonists improved other patient-important outcomes. Low-to-moderate quality evidence supports improved outcomes with surgery and radiotherapy compared to no treatment in patients who were resistant to or intolerant of dopamine agonists.

**Conclusion:**

Our results provide evidence to support the use of dopamine agonists in reducing prolactin levels and persistent hyperprolactinemia, with cabergoline proving more efficacious than bromocriptine. Radiotherapy and surgery are useful in patients with resistance or intolerance to dopamine agonists.

## Background

Hyperprolactinemia is the most common disorder of the hypothalamic-pituitary axis. Patients typically present with hypogonadism, infertility or, in the case of macroadenomas, symptoms related to mass effect (headache and visual field defects).

In general, treatment of hyperprolactinemia, secondary to pituitary macroadenoma, is accepted as necessary. Medications in the form of dopamine agonists are the first line of treatment, with surgery and radiotherapy reserved for refractory and medication-intolerant patients [1]. The primary aim of treatment in patients with pituitary macroadenoma is to control compressive effects of the tumor, including compression of optic chiasm, with a secondary goal to restore gonadal function. However, indications and modalities of treatment of hyperprolactinemia due to pituitary microadenomas are less well defined [1]. Commonly cited indications for treatment of microprolactinomas include infertility, hypogonadism, prevention of bone loss and bothersome galactorrhea [1,2]. Treatment with dopamine agonists can restore normal prolactin levels and gonadal function. Dopamine agonists have been associated with various adverse effects including nausea, vomiting, psychosis and dyskinesia. However, the choice of which dopamine agonist is most efficacious and produces the least adverse effects is unclear.

To provide evidence-based recommendations to practicing clinicians facing these common therapeutic dilemmas, we conducted a systematic review and meta-analyses of the literature to evaluate outcomes and adverse effects with medications, surgery and radiotherapy in hyperprolactinemic patients. Outcomes of interest include prolactin levels, tumor size, and persistent hyperprolactinemia and patient-important outcomes, including visual disturbances, fertility, sexual dysfunction and galactorrhea,

## Methods

The results are reported according to the PRISMA statement (Preferred reporting items for systematic reviews and meta-analyses) [3]. We used the relevant components of the Ottawa-Newcastle tool (whether cohorts represent clinical practice, blinding of outcome assessment, analysis adjustment for confounders, and adequacy of follow-up) [4] and the Cochrane risk of bias tool (extent of blinding, allocation concealment, and funding) to evaluate the quality of observational and randomized trials, respectively. Summary judgments about the quality of evidence for each outcome followed the GRADE (Grading of Recommendations Assessment, Development, and Evaluation) framework (Additional file 1: Tables 2–4) [5].

### Study eligibility

Eligible studies provided longitudinal follow-up data of cohorts of patients with hyperprolactinemia, that is, observational cohort studies or randomized controlled trials (RCTs). The outcomes of interest were tumor size, visual field defects, prolactin levels, galactorrhea, infertility, hypogonadism (amenorrhea/oligomenorrhea and low libido for premenopausal women, low libido or erectile dysfunction for men), bone density loss and fragility fracture rates, quality of life, and treatment adverse effects. We assumed author reports of “irregular menses” to mean amenorrhea or oligomenorrhea unless otherwise specified. We included studies with follow-up duration of at least six months and studies of at least 10 subjects. We did not impose any language restrictions.

### Search strategy

We sought articles addressing hyperprolactinemia or prolactin-secreting tumors that were treated by dopamine agonists, surgery or radiotherapy, which focused on outcomes from those treatments. The search concepts of hyperprolactinemia, outcomes of interest (specific sequelae of amenorrhea/oligomenorrhea, sexual dysfunction, vision disorders, cranial nerve disorders and bone disorders), treatments and study design (observational longitudinal studies or RCTs) were represented in the search strategy using database-specific controlled vocabulary. We searched in Ovid MEDLINE, Ovid EMBASE and the Ovid Cochrane Library, ISI Web of Science and Scopus from inception through September 2009. The search was updated on 15 December 2011. The complete search strategy was done with the help of an experienced research librarian and is available in the Additional file 1: Appendix.

### Study selection

Study selection and data extraction procedures were conducted by pairs of reviewers working independently until adequate agreement (kappa ≥ 0.90) was obtained; then the process was conducted by single reviewers. First, eligibility criteria were applied to titles and abstracts, and potentially eligible studies were retrieved in full text. Then, eligibility criteria were applied to the full report. Disagreements were noted and resolved by discussion and consensus, erring on inclusion. We extracted descriptive data about enrolled patients, any treatment provided, study quality measures and outcome data from each study. Both study selection and data extraction were conducted using web-based software (Distiller SR, Ottawa, ON, Canada).

### Statistical analysis

The effect size and the associated measures of precision were estimated from each study (relative risk (RR) for dichotomous outcomes, weighted mean difference (WMD) for continuous outcomes, and event rate for uncontrolled studies).

Effect sizes were pooled across studies using a random effects meta-analytical model [6]. Heterogeneity was assessed using the I^2^ statistic, which represents the proportion of between-study differences that are not attributable to chance or random error [7]. I^2^ values of <25%, 25 to 50% and >50% indicate mild, moderate and substantial heterogeneity, respectively. When meta-analysis includes less than three studies, the I^2^ is not calculable and is not reported. *A priori* planned subgroup interactions were based on sex and size of tumor (macro- vs. microprolactinomas). Median and range of event rates were estimated from uncontrolled cohort studies or case series that did not provide sufficient data for meta-analysis. All analyses were completed using *Comprehensive Meta Analysis Version 2.2*, Biostat, Englewood NJ (2005).

## Results

**Figure 1:**
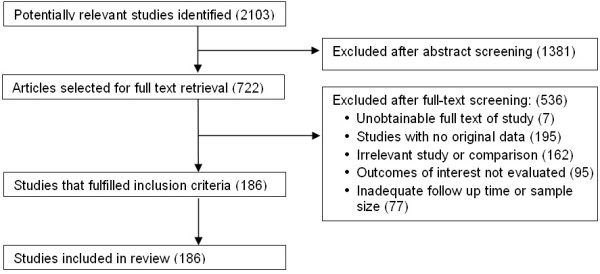
Study selection process.

Literature search revealed 2,103 potentially relevant references, of which 189 were included (Figure 1). The description, quality assessment and bibliography of the studies are available in the Additional file 1: Appendix.

**Figure 2:**
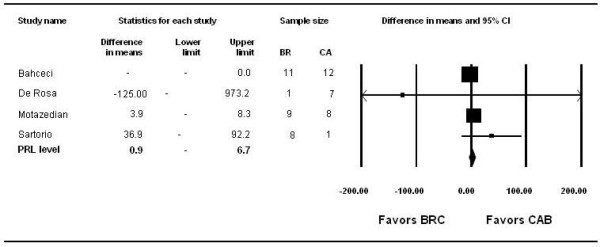
Bromocriptine vs. Cabergoline: prolactin levels.

**Figure 3:**
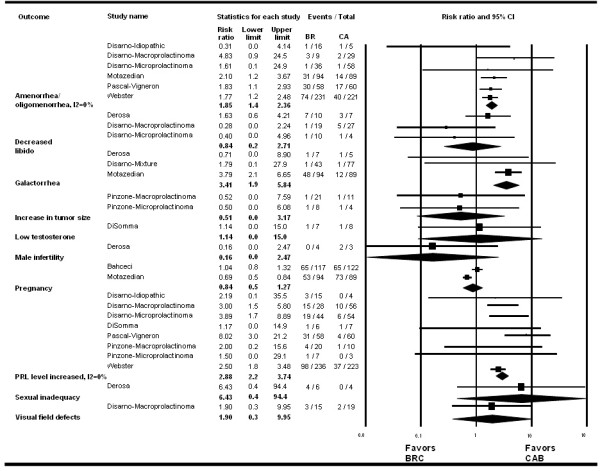
Bromocriptine vs. Cabergoline: clinical outcomes.

Twenty-nine studies were controlled (that is, two arms allowing for comparative analysis) (Additional file 1: Table 1), whereas 157 were uncontrolled (that is, the entire cohort received the same intervention allowing the estimation of event rates but no comparative analysis). We contacted the authors of the comparative studies via e-mail if possible and by postal mail if no e-mail was available; 20 authors replied, of which 15 confirmed or corrected study data.

### Study quality

The quality of the observational studies was limited, with no blinding of outcome assessment and poor reporting of adjustments for confounders or other prognostic variables (Additional file 1: Tables 2 and 3). The quality of the eight RCTs [8-14] was fair (allocation was concealed in five; patients were blinded to assignment in six RCTs, caregivers in five) (Additional file 1: Table 4).

### Patients treated with dopamine agonists

A large body of noncomparative cohort studies supported the use of dopamine agonists in patients with hyperprolactinemia. Those studies included: bromocriptine (n = 39); cabergoline (n = 26); and quinagolide (CV 205-502) (n = 15), which is not approved in the US. Bromocriptine studies had the longest follow-up (exceeding 10 years) and showed consistent benefits on several patient-important outcomes and surrogate outcomes (Additional file 1: Table 5A). Comparing across studies, 68% (median %) of patients treated with bromocriptine had normalization of prolactin levels and 62% experienced a reduction in tumor size. Bromocriptine also successfully treated other major outcomes, including 86% of patients with galactorrhea, 78% with amenorrhea, 67% with sexual dysfunction, 67% with visual field defects and 53% of patients with infertility. Studies of cabergoline and quinagolide showed similar results (Additional file 1: Tables 5B, C). In three observational studies that followed patients from 7 to 12 months, long-acting forms of bromocriptine were found to be as efficacious as the short-acting forms (Additional file 1: Table 5D). Other dopamine agonists typically used for other conditions, such as Parkinson’s disease, were also used in this setting; namely, pergolide, lisuride, and roxindol (Additional file 1: Table 5E), with comparable findings.

A smaller body of evidence offers comparative effectiveness data from observational studies and eight RCTs. Forest plots depicting the results of these meta-analyses are in Additional file 1: Figures 1A-5B. The results are presented by comparison.

· *Bromocriptine* vs. *Cabergoline* (Figures 2 and 3): Six observational studies and three randomized trials compared bromocriptine to cabergoline. Bromocriptine was less effective than cabergoline in reducing the risk of persistent hyperprolactinemia (RR, 2.88; 95% CI, 2.20 to 3.74; I^2^ = 0%), amenorrhea/oligomenorrhea (RR, 1.85; 95% CI, 1.40 to 2.36), and galactorrhea (RR, 3.41; 95% CI, 1.9 to 5.84). There were no significant differences between the two drugs in terms of overall change in prolactin level or other patient-important outcomes.

· *Bromocriptine* vs. *Quinagolide:* Two observational studies and four RCTs compared quinagolide to bromocriptine. There were no significant differences between these agents across all outcomes reviewed (Additional file 1: Figures 1A and 1B).

· *Dopamine agonists compared to no treatment:* Three observational studies and one RCT compared dopamine agonists to no treatment. Dopamine agonists significantly reduced prolactin level (WMD, -45; 95% CI, -77 to -11) and the risk of persistent hyperprolactinemia (RR, 0.9; 95% CI, 0.81 to 0.99) but not other patient-important outcomes (Additional file 1: Figures 2A and 2B).

· *Comparison of dopamine agonists* vs. *surgery and combinations thereof:* Additional file 1: Figures 3-5B depict comparisons between surgery vs. dopamine agonists, dopamine agonists vs. dopamine agonists + surgery, and surgery vs. surgery + dopamine agonists. The only significant difference among these comparisons was dopamine agonists were more effective in reducing the risk of persistent hyperprolactinemia compared to surgery alone.

The quality of evidence in this comparison for all outcomes is very low due to methodological limitations of included studies and the serious imprecision of meta-analytic estimates that include both trivial and large effects. Subgroup analyses for these comparisons (Additional file 1: Table 6) did not reveal a significant interaction based on sex or tumor size (macro- vs. microprolactinoma).

### Patients treated with other modalities

Other treatments, such as radiotherapy, surgery and combinations of treatments were evaluated in an uncontrolled series of patients. Meta-analysis was not conducted due to the significant clinical heterogeneity in terms of patient characteristics and symptomatology as well as the heterogeneity of study settings, design and follow-up duration.

Radiotherapy was evaluated in eight studies with follow-up of at least two years. In patients with medically and surgically refractory prolactinomas, radiotherapy produced a reduction in prolactin levels in nearly all patients and normalization in over a quarter of patients with low complication rates (Additional file 1: Table 7A). External and implanted radiotherapy methods were also used in conjunction with dopamine agonists and resulted in significant improvement in prolactin levels, visual symptoms and fertility (four studies with follow-up of between 12 and 140 months, Additional file 1: Table 7B).

Trans-sphenoidal surgery for pituitary adenomas was evaluated in 27 uncontrolled studies (Additional file 1: Table 7C) and was found to be effective in normalizing prolactin levels and resolving symptoms. Patients opting for this approach had often failed other management options and may have had a worse prognosis that was independent of the treatment; this selection bias may underestimate the effectiveness of surgery. In five studies, a combination of surgery and dopamine agonists achieved high rates of prolactin normalization and had relatively low rates of recurrence (Additional file 1: Table 7D). In two studies (Additional file 1: Table 7E), surgery combined with radiotherapy was also seen to be effective.

### Adverse effects

Commonly reported side effects for all dopamine agonists included nausea, dizziness, postural hypotension and headache. In studies comparing cabergoline and bromocriptine, side effects were less frequent and milder with cabergoline compared to bromocriptine. In one study, 18%, 18%, 9% and 3% of patients experienced nausea, hypotension, headache and vomiting, respectively, compared with 44 21%, 27% and 20% in patients receiving bromocriptine [Motazedian, 2010, #105]. Bahceci found an overall side effect rate of 2.5% for cabergoline versus 15.3% for bromocriptine [Bahceci, 2010, #103]. Another study found a 29% overall side effect rate for cabergoline vs. 70% with bromocriptine, and that cabergoline side effects were more mild, self-limited, and did not require intervention, compared to bromocriptine side effects which required dose reduction and intervention in 29% of cases [De Rosa, 1998, #104]. Non-comparative studies revealed similar findings with the most common side effects of dopamine agonists being nausea, vomiting, headache, hypotension, with rare side effects of rhinorrhea and hypotonia. Adverse effects reported with transsphenoidal surgery included cerebrospinal fluid leak, diabetes insipidus, rhinorrhea and hypopituitarism, while radiotherapy was associated with nausea, headache, visual disturbances and hearing loss.

### Pregnancy studies

Twenty studies followed pregnant women and their offspring from 6 months up to 12 years (Additional file 1: Table 7F). A fairly consistent finding was that there was no significant increase in the risk of obstetric complications, miscarriages, fetal malformation or other pregnancy outcomes, even if they had been treated with dopamine agonists to induce ovulation. The quality of this evidence is low considering the lack of contemporary untreated control groups in most studies or the enrollment of nonconsecutive samples of patients.

## Discussion

The two most commonly prescribed drugs in the treatment of hyperprolactinemia are bromocriptine and cabergoline. Both medications are dopamine receptor agonists and share many characteristics and adverse effects, such as headache, nausea and vomiting, among others, though frequency and severity of adverse effects appears to be less in cabergoline compared to bromocriptine. Previous concerns about valvular heart disease [15,16] with the use of these agents have largely been disproved by more recent reports [17-19]. Our review demonstrated that cabergoline was significantly better than bromocriptine in decreasing the risks of persistent hyperprolactinemia, amenorrhea/oligomenorrhea and galactorrhea. Frequency of dosing may also affect treatment decisions as cabergoline is dosed twice weekly, whereas bromocriptine is given daily. However, cabergoline costs at least twice as much as bromocriptine and was not found to be superior in other outcomes. Though both drugs have been found to be safe in pregnancy, the number of reports studying bromocriptine in pregnancy far exceeds that of cabergoline in pregnancy.

A large body of moderate quality evidence from observational studies supports the use of dopamine agonists to normalize prolactin levels and resolve the symptoms related to mass effect and elevated prolactin levels. The large treatment effect of dopamine agonists, the potential dose response effect, biological plausibility, temporality between treatment and effect, consistency across studies, settings and methods, and coherence (consistency across agents within the same class), strongly support the effectiveness of these treatment agents in reducing prolactin levels and improving symptoms [20]. In addition, the recurrence of hyperprolactinemia after withdrawal of dopamine agonists strengthens the inference about causality (that is, challenge-rechallenge phenomenon). Clinicians using these medications are well aware of potential adverse effects that sometimes limit use, which include nausea, vomiting, psychosis and dyskinesia, among others.

Efficacy of surgery and radiotherapy in selected patients is also substantiated, although by low-to-moderate quality evidence at higher risk of bias. Radiotherapy and surgery appear to be efficacious in patients with resistance or intolerance to dopamine agonists. However, surgery as a primary therapy has also been described in a recent consecutive series of 212 patients with prolactinomas [21]. This study reports high short-term remission rates, particularly in patients with microadenomas and cystic tumors. Besides the usual surgical risks, hypopituitarism is a potential long-term effect of both radiotherapy and surgery and should be discussed with patients as part of the decision-making process.

### Comparison with previous reviews, strengths and limitations

Only a few previous systematic reviews have been published in this field, and to the best of our knowledge, this is the first to comprehensively address the efficacy questions outlined in our protocol. Our work is also referenced as unpublished data in the 2011 Endocrine Society Clinical Practice Guideline: Diagnosis and Treatment of Hyperprolactinemia [22]. Our results are similar to other reviews, including Dekkers’ meta-analysis of the sustainability of normoprolactinemia after treatment withdrawal, which found recurrences in a substantial proportion of patients [23], and Dos Santos Nunes’ systematic review and meta-analysis of four randomized controlled trials, which demonstrated that normalization of prolactin levels and menstruation favored cabergoline compared to bromocriptine [24].

The strengths of our review include the comprehensive nature of the literature search, the immediate relevancy of the questions at hand to decision making, and the adoption of bias protection measures that included contacting the authors of the included studies. Limitations to the inferences presented in this report relate to the overall low quality of evidence due to the methodological limitations of the included studies, and by the imprecision and heterogeneity in the results. Also, this evidence is at high risk of publication and reporting biases, both of which are more likely when the evidence consists of mostly small RCTs and observational studies. Inferences should also be limited considering the frequent use of the surrogate outcome, prolactin level, as opposed to patient-important outcomes [25], such as loss of quality of life due to tumor-related and hypogonadal symptoms.

## Conclusion

This systematic review and meta-analyses affirm the use of dopamine agonists in treating hyperprolactinemia and reducing associated morbidity. Cabergoline was found to be more effective than bromocriptine in achieving normoprolactinemia and resolving amenorrhea/oligomenorrhea and galactorrhea. Radiotherapy and surgery are efficacious in patients with resistance or intolerance to dopamine agonists.

## Abbreviations

PRISMA, Preferred reporting items for systematic reviews and meta-analyses; RCT, Randomized controlled trial; RR, Relative risk; WMD, Weighted mean difference.

## Competing interests

The authors declare that they have no competing interests.

## Authors’ contributions

ATW, PJE, GYG, VMM and MHM were responsible for the study’s conception and design. ATW, RJM, MAL, AH, CP, NWG, MMF, AB, FC, JC, TAE and, PJE acquired the data. ATW, RJM, MAL, TAE and MHM analyzed and interpreted the data. All the authors were responsible for drafting, critical revisions, and final approval of the manuscript.

## Acknowledgements

This research was partially funded by the Endocrine Society.

## Supplementary Material

Additional file 1 **Appendix. **Treatment of hyperprolactinemia: a systematic review and meta-analysis*.* Description of data: Contents: Baseline characteristics of the included comparative studies (Supplemental Table 1)*,* Quality of the included observational comparative studies (Supplemental Table 2)*,* Quality of the included observational dopamine withdrawal studies (Supplemental Table 3)*,* Quality of randomized trials (Supplemental Table 4)*,* Summary of uncontrolled studies of dopamine agonists (Supplemental Tables 5A-E)*,* Meta-analyses figures (Supplemental Figures 1A-5B), Subgroup analyses (Supplemental Tables 6A-D), Summary of uncontrolled studies of radiotherapy, surgery, combinations of treatment, and pregnancy (Supplemental Tables 7A-F), References, Search strategy [26-205]. Click here for file

## References

[B1] Casanueva FF Molitch ME Schlechte JA Abs R Bonert V Bronstein MD Brue T Cappabianca P Colao A Fahlbusch R Guidelines of the Pituitary Society for the diagnosis and management of prolactinomas Clin Endocrinol 2006 65 265 273 10.1111/j.1365-2265.2006.02562.x 16886971

[B2] Gillam M Molitch M Lombardi G Colao A Advances in the treatment of prolactinomas Endocr Rev 2006 27 485 534 10.1210/er.2005-9998 16705142

[B3] Moher D Liberati A Tetzlaff J Altman DG Preferred reporting items for systematic reviews and meta-analyses: the PRISMA statement BMJ 2009 339 b2535 10.1136/bmj.b2535 19622551 PMC2714657

[B4] Wells G Shea B O'Connell D Peterson J Welch V Losos M Tugwell P The Newcastle-Ottawa Scale (NOS) for assessing the quality of nonrandomised studies in meta-analyses[http://www.ohri.ca/programs/clinical_epidemiology/oxford.htm]

[B5] Swiglo BA Murad MH Schunemann HJ Kunz R Vigersky RA Guyatt GH Montori VM A case for clarity, consistency, and helpfulness: state-of-the-art clinical practice guidelines in endocrinology using the grading of recommendations, assessment, development, and evaluation system J Clin Endocrinol Metab 2008 93 666 673 18171699 10.1210/jc.2007-1907

[B6] DerSimonian R Laird N Meta-analysis in clinical trials Control Clin Trials 1986 7 177 188 10.1016/0197-2456(86)90046-2 3802833

[B7] Higgins JP Thompson SG Deeks JJ Altman DG Measuring inconsistency in meta-analyses BMJ 2003 327 557 560 10.1136/bmj.327.7414.557 12958120 PMC192859

[B8] Hirahara F Andoh N Sawai K Hirabuki T Uemura T Minaguchi H Hyperprolactinemic recurrent miscarriage and results of randomized bromocriptine treatment trials Fertil Steril 1998 70 246 252 10.1016/S0015-0282(98)00164-2 9696215

[B9] Homburg R West C Brownell J Jacobs HS A double-blind study comparing a new non-ergot, long-acting dopamine agonist, CV 205-502, with bromocriptine in women with hyperprolactinaemia Clin Endocrinol 1990 32 565 571 10.1111/j.1365-2265.1990.tb00899.x 1973085

[B10] Lappohn RE van de Wiel HB Brownell J The effect of two dopaminergic drugs on menstrual function and psychological state in hyperprolactinemia Fertil Steril 1992 58 321 327 1353028 10.1016/s0015-0282(16)55201-7

[B11] Pascal-Vigneron V Weryha G Bosc M Leclere J Hyperprolactinemic amenorrhea:treatment with cabergoline versus bromocriptine. Results of a national multicenter randomized double-blind study Presse Med 1995 24 753 757 7784413

[B12] van der Heijden PF de Wit W Brownell J Schoemaker J Rolland R CV 205-502, a new dopamine agonist, versus bromocriptine in the treatment of hyperprolactinaemia Eur J Obstet Gynecol Reprod Biol 1991 40 111 118 10.1016/0028-2243(91)90101-P 1676973

[B13] Verhelst JA Froud AL Touzel R Wass JA Besser GM Grossman AB Acute and long-term effects of once-daily oral bromocriptine and a new long-acting non-ergot dopamine agonist, quinagolide, in the treatment of hyperprolactinemia: a double-blind study Acta Endocrinol 1991 125 385 391 1683503 10.1530/acta.0.1250385

[B14] Webster J Piscitelli G Polli A Ferrari C Ismail I Scanlon M A comparison of cabergoline and bromocriptine in the treatment of hyperprolactinemic amenorrhea. Cabergoline Comparative Study Group N Engl J Med 1994 331 904 909 10.1056/NEJM199410063311403 7915824

[B15] Motazedian S Babakhani L Fereshtehnejad SM Mojtahedi K A comparison of bromocriptine & cabergoline on fertility outcome of hyperprolactinemic infertile women undergoing intrauterine insemination Indian J Med Res 2010 131 670 674 20516539

[B16] Bahceci M Sismanoglu A Ulug U Comparison of cabergoline and bromocriptine in patients with asymptomatic incidental hyperprolactinemia undergoing ICSI-ET Gynecol Endocrinol 2010 26 505 508 10.3109/09513591003632233 20459348

[B17] De Rosa M Colao A Di Sarno A Ferone D Landi ML Zarrilli S Paesano L Merola B Lombardi G Cabergoline treatment rapidly improves gonadal function in hyperprolactinemic males: a comparison with bromocriptine Eur 1998 138 286 293 10.1530/eje.0.1380286 9539303

[B18] Horvath J Fross RD Kleiner-Fisman G Lerch R Stalder H Liaudat S Raskoff WJ Flachsbart KD Rakowski H Pache JC Severe multivalvular heart disease: a new complication of the ergot derivative dopamine agonists Mov Disord 2004 19 656 662 10.1002/mds.20201 15197703

[B19] Rascol O Pathak A Bagheri H Montastruc JL New concerns about old drugs: Valvular heart disease on ergot derivative dopamine agonists as an exemplary situation of pharmacovigilance Mov Disord 2004 19 611 613 10.1002/mds.20202 15197697

[B20] Lafeber M Stades AME Valk GD Cramer MJ van Berkhout FT Zelissen PMJ Absence of major fibrotic adverse events in hyperprolactinemic patients treated with cabergoline Eur 2010 162 667 675 10.1530/EJE-09-0989 20071478

[B21] Tan T Cabrita IZ Hensman D Grogono J Dhillo WS Baynes KC Eliahoo J Meeran K Robinson S Nihoyannopoulos P Martin NM Assessment of cardiac valve dysfunction in patients receiving cabergoline treatment for hyperprolactinaemia Clin Endocrinol 2010 73 369 374 10.1111/j.1365-2265.2010.03827.x 20550538

[B22] Valassi E Klibanski A Biller BM Clinical Review#: Potential cardiac valve effects of dopamine agonists in hyperprolactinemia J Clin Endocrinol Metab 2010 95 1025 1033 10.1210/jc.2009-2095 20130078

[B23] Glasziou P Chalmers I Rawlins M McCulloch P When are randomised trials unnecessary? Picking signal from noise BMJ 2007 334 349 351 10.1136/bmj.39070.527986.68 17303884 PMC1800999

[B24] Kreutzer J Buslei R Wallaschofski H Hofmann B Nimsky C Fahlbusch R Buchfelder M Operative treatment of prolactinomas: indications and results in a current consecutive series of 212 patients Eur J Endocrinol 2008 158 11 18 10.1530/EJE-07-0248 18166812

[B25] Melmed S Casanueva FF Hoffman AR Kleinberg DL Montori VM Schlechte JA Wass JA Diagnosis and treatment of hyperprolactinemia: an Endocrine Society clinical practice guideline J Clin Endocrinol Metab 2011 96 273 288 10.1210/jc.2010-1692 21296991

[B26] Dekkers O Lagro J Burman P Jørgensen J Romijn J Pereira A Recurrence of hyperprolactinemia after withdrawal of dopamine agonists: systematic review and meta-analysis J Clin Endocrinol Metab 2010 95 43 51 10.1210/jc.2009-1238 19880787

[B27] Dos Santos Nunes V El Dib R Boguszewski CL Nogueira CR Cabergoline versus bromocriptine in the treatment of hyperprolactinemia: a systematic review of randomized controlled trials and meta-analysis Pituitary 2011 14 259 265 10.1007/s11102-010-0290-z 21221817

[B28] Gandhi GY Murad MH Fujiyoshi A Mullan RJ Flynn DN Elamin MB Swiglo BA Isley WL Guyatt GH Montori VM Patient-important outcomes in registered diabetes trials JAMA 2008 299 2543 2549 10.1001/jama.299.21.2543 18523223

[B29] Asano S Ueki K Suzuki I Kirino T Clinical features and medical treatment of male prolactinomas Acta Neurochir 2001 143 465 470 10.1007/s007010170075 11482696

[B30] Brue T Pellegrini I Priou A Morange I Jaquet P Prolactinomas and resistance to dopamine agonists Horm Res 1992 38 84 89 10.1159/000182496 1306523

[B31] Candrina R Galli G Bollati A Pizzocolo G Orlandini A Gualandi GF Giustina G Results of combined surgical and medical therapy in patients with prolactin-secreting pituitary macroadenomas Neurosurgery 1987 21 894 897 10.1227/00006123-198712000-00018 3437957

[B32] Colao A Merola B Sarnacchiaro F Di Sarno A Landi ML Marzullo P Cerbone G Ferone D Lombardi G Comparison among different dopamine-agonists of new formulation in the clinical management of macroprolactinomas Horm Res 1995 44 222 228 10.1159/000184630 8582715

[B33] Di Sarno A Landi ML Cappabianca P Di Salle F Rossi FW Pivonello R Di Somma C Faggiano A Lombardi G Colao A Resistance to cabergoline as compared with bromocriptine in hyperprolactinemia: prevalence, clinical definition, and therapeutic strategy J Clin Endocrinol Metab 2001 86 5256 5261 10.1210/jc.86.11.5256 11701688

[B34] Di Somma C Colao A Di Sarno A Klain M Landi ML Facciolli G Pivonello R Panza N Salvatore M Lombardi G Bone marker and bone density responses to dopamine agonist therapy in hyperprolactinemic males J Clin Endocrinol Metab 1998 83 807 813 10.1210/jc.83.3.807 9506732

[B35] Hildebrandt G Bauer T Stracke H Fassbender WJ Mueller HW Agnoli AL Federlin K Roosen K Surgery, dopamine agonist therapy of combined treatment–results in prolactinoma patients after a 12 month follow-up Zentralbl Neurochir 1992 53 123 134 1414079

[B36] Jeffcoate WJ Pound N Sturrock ND Lambourne J Long-term follow-up of patients with hyperprolactinaemia Clin Endocrinol (Oxf) 1996 45 299 303 10.1046/j.1365-2265.1996.00824.x 8949567

[B37] Mahmood IH Al-Husaynei AJ Mohamad SH Comparative effects of bromocriptine and cabergoline on serum prolactin levels, liver and kidney function tests in hyperprolactinemic women Pak J Med Sci 2010 26 255 260

[B38] Mattei AM Severini V Crosignani PG Natural history of hyperprolactinemia Ann N Y Acad Sci 1991 626 130 136 10.1111/j.1749-6632.1991.tb37907.x 2058949

[B39] Perrin G Treluyer C Trouillas J Sassolas G Goutelle A Surgical outcome and pathological effects of bromocriptine preoperative treatment in prolactinomas Pathol Res Pract 1991 187 587 592 10.1016/S0344-0338(11)80151-2 1923955

[B40] Pinzone JJ Katznelson L Danila DC Pauler DK Miller CS Klibanski A Primary medical therapy of micro- and macroprolactinomas in men.[see comment] J Clin Endocrinol Metab 2000 85 3053 3057 10.1210/jc.85.9.3053 10999785

[B41] Rush S Donahue B Cooper P Lee C Persky M Newall J Prolactin reduction after combined therapy for prolactin macroadenomas Neurosurgery 1991 28 502 505 10.1227/00006123-199104000-00003 2034342

[B42] Samaan NA Schultz PN Leavens TA Leavens ME Lee YY Pregnancy after treatment in patients with prolactinoma: operation versus bromocriptine Am J Obstet Gynecol 1986 155 1300 1305 3789042 10.1016/0002-9378(86)90164-x

[B43] Sartorio A Conti A Ambrosi B Muratori M Morabito F Faglia G Osteocalcin levels in patients with microprolactinoma before and during medical treatment J Endocrinol Invest 1990 13 419 422 1974270 10.1007/BF03350694

[B44] Shih CJ Tsou CK Chiu WT Tsai SH Management of prolactin-secreting pituitary adenomas with surgery and bromocriptine Southeast Asian J Surg 1983 6 38 46

[B45] Sluijmer AV Lappohn RE Clinical history and outcome of 59 patients with idiopathic hyperprolactinemia Fertil Steril 1992 58 72 77 1624026 10.1016/s0015-0282(16)55139-5

[B46] Torres I Carral F Vilchez F Gavilán J Aguilar M Clinical, radiologic, and follow-up findings in patients with macroprolactinoma Endocrinologist 2006 16 241 244 10.1097/01.ten.0000240933.97458.bf

[B47] Touraine P Plu-Bureau G Beji C Mauvais-Jarvis P Kuttenn F Long-term follow-up of 246 hyperprolactinemic patients Acta Obstet Gynecol Scand 2001 80 162 168 10.1034/j.1600-0412.2001.080002162.x 11167213

[B48] Webster J Piscitelli G Polli A Ferrari CI Ismail I Scanlon MF A comparison of cabergoline and bromocriptine in the treatment of hyperprolactinemic amenorrhea. Cabergoline Comparative Study Group N Engl J Med 1994 331 904 909 10.1056/NEJM199410063311403 7915824

[B49] Biswas M Smith J Jadon D McEwan P Rees DA Evans LM Scanlon MF Davies JS Long-term remission following withdrawal of dopamine agonist therapy in subjects with microprolactinomas Clin Endocrinol 2005 63 26 31 10.1111/j.1365-2265.2005.02293.x 15963057

[B50] Cannavo S Curto L Squadrito S Almoto B Vieni A Trimarchi F Cabergoline: a first-choice treatment in patients with previously untreated prolactin-secreting pituitary adenoma J Endocrinol Invest 1999 22 354 359 10401709 10.1007/BF03343573

[B51] Ciccarelli E Grottoli S Razzore P Gaia D Bertagna A Cirillo S Cammarota T Camanni M Camanni F Long-term treatment with cabergoline, a new long-lasting ergoline derivate, in idiopathic or tumorous hyperprolactinaemia and outcome of drug-induced pregnancy J Endocrinol Invest 1997 20 547 551 9413809 10.1007/BF03348017

[B52] Colao A Di Sarno A Cappabianca P Di Somma C Pivonello R Lombardi G Withdrawal of long-term cabergoline therapy for tumoral and nontumoral hyperprolactinemia.[see comment] N Engl J Med 2003 349 2023 2033 10.1056/NEJMoa022657 14627787

[B53] Corenblum B Taylor PJ Idiopathic hyperprolactinemia may include a distinct entity with a natural history different from that of prolactin adenomas Fertil Steril 1988 49 544 546 3342909 10.1016/s0015-0282(16)59790-8

[B54] Di Sarno A Landi ML Marzullo P Di Somma C Pivonello R Cerbone G Lombardi G Colao A The effect of quinagolide and cabergoline, two selective dopamine receptor type 2 agonists, in the treatment of prolactinomas Clin Endocrinol (Oxf) 2000 53 53 60 10.1046/j.1365-2265.2000.01016.x 10931080

[B55] Eversmann T Fahlbusch R Rjosk HK von Werder K Persisting suppression of prolactin secretion after long-term treatment with bromocriptine in patients with prolactinomas Acta Endocrinol (Copenh) 1979 92 413 427 574700 10.1530/acta.0.0920413

[B56] Hancock KW Scott JS Lamb JT Gibson RM Chapman C Long term suppression of prolactin concentrations after bromocriptine induced regression of pituitary prolactinomas Br Med J (Clin Res Ed) 1985 290 117 118 10.1136/bmj.290.6462.117-a PMC1415489 3917709

[B57] Johnston DG Prescott RW Kendall-Taylor P Hall K Crombie AL Hall R McGregor A Watson MJ Cook DB Hyperprolactinemia. Long-term effects of bromocriptine Am J Med 1983 75 868 874 10.1016/0002-9343(83)90418-7 6638052

[B58] Kharlip J Salvatori R Yenokyan G Wand GS Recurrence of hyperprolactinemia after withdrawal of long-term cabergoline therapy.[see comment] J Clin Endocrinol Metab 2009 94 2428 2436 10.1210/jc.2008-2103 19336508 PMC2708963

[B59] Liuzzi A Dallabonzana D Oppizzi G Verde GG Cozzi R Chiodini P Luccarelli G Low doses of dopamine agonists in the long-term treatment of macroprolactinomas N Engl J Med 1985 313 656 659 10.1056/NEJM198509123131103 4022058

[B60] Mattei AM Ferrari C Ragni G Benco R Picciotti MC Rampini P Caldara R Crosignani PG Serum prolactin and ovarian function after discontinuation of drug treatment for hyperprolactinaemia: a study with bromocriptine and metergoline Br J Obstet Gynaecol 1984 91 244 250 10.1111/j.1471-0528.1984.tb04761.x 6704349

[B61] Moriondo P Travaglini P Nissim M Conti A Faglia G Bromocriptine treatment of microprolactinomas: evidence of stable prolactin decrease after drug withdrawal J Clin Endocrinol Metab 1985 60 764 772 10.1210/jcem-60-4-764 3919052

[B62] Muratori M Arosio M Gambino G Romano C Biella O Faglia G Use of cabergoline in the long-term treatment of hyperprolactinemic and acromegalic patients J Endocrinol Invest 1997 20 537 546 9413808 10.1007/BF03348016

[B63] Passos VQ Souza JJ Musolino NR Bronstein MD Long-term follow-up of prolactinomas: normoprolactinemia after bromocriptine withdrawal J Clin Endocrinol Metab 2002 87 3578 3582 10.1210/jc.87.8.3578 12161478

[B64] Tartagni M Nicastri PL Diaferia A Di Gesu I Loizzi P Long-term follow-up of women with amenorrhea-galactorrhea treated with bromocriptine Clin Exp Obstet Gynecol 1995 22 301 306 8777784

[B65] van 't Verlaat JW Croughs RJ Withdrawal of bromocriptine after long-term therapy for macroprolactinomas; effect on plasma prolactin and tumour size.[see comment] Clin Endocrinol (Oxf) 1991 34 175 178 10.1111/j.1365-2265.1991.tb00289.x 2036725

[B66] Winkelmann W Allolio B Deuss U Heesen D Kaulen D Macleod RM, Thorner MO, Scapagnini U Persisting normoprolactinemia after withdrawal of bromocriptine long-term therapy in patients with prolactinomas Basic and Clinical Correlates 1985 Liviana Press, Padova 817 822

[B67] Wu ZB Yu CJ Su ZP Zhuge QC Wu JS Zheng WM Bromocriptine treatment of invasive giant prolactinomas involving the cavernous sinus: results of a long-term follow up J Neurosurg 2006 104 54 61 10.3171/jns.2006.104.1.54 16509147

[B68] Zarate A Canales ES Cano C Pilonieta CJ Follow-up of patients with prolactinomas after discontinuation of long-term therapy with bromocriptine Acta Endocrinol (Copenh) 1983 104 139 142 6356741 10.1530/acta.0.1040139

[B69] Al-Suleiman SA Najashi S Rahman J Rahman MS Outcome of treatment with bromocriptine in patients with hyperprolactinaemia Aust N Z J Obstet Gynaecol 1989 29 176 179 10.1111/j.1479-828X.1989.tb01712.x 2803131

[B70] Bergh T Nillius J Wide L Bromocriptine treatment of 42 hyperprolactinaemic women with secondary amenorrhoea Acta Endocrinol (Copenh) 1978 88 435 451 354299 10.1530/acta.0.0880435

[B71] Brue T Lancranjan I Louvet JP Dewailly D Roger P Jaquet P A long-acting repeatable form of bromocriptine as long-term treatment of prolactin-secreting macroadenomas: a multicenter study Fertil Steril 1992 57 74 80 1730334

[B72] Cannavo S De Natale R Curto L Li Calzi L Trimarchi F Effectiveness of computer-assisted perimetry in the follow-up of patients with pituitary microadenoma responsive to medical treatment Clin Endocrinol 1992 37 157 161 10.1111/j.1365-2265.1992.tb02300.x 1395066

[B73] Chattopadhyay A Bhansali A Masoodi SR Long-term efficacy of bromocriptine in macroprolactinomas and giant prolactinomas in men Pituitary 2005 8 147 154 10.1007/s11102-005-5111-4 16379032

[B74] Corenblum B Taylor PJ Long-term follow-up of hyperprolactinemic women treated with bromocriptine Fertil Steril 1983 40 596 599 6628704 10.1016/s0015-0282(16)47415-7

[B75] Espinos JJ Rodriguez-Espinosa J Webb SM Calaf-Alsina J Long-acting repeatable bromocriptine in the treatment of patients with microprolactinoma intolerant or resistant to oral dopaminergics Fertil Steril 1994 62 926 931 7926136 10.1016/s0015-0282(16)57052-6

[B76] Essais O Bouguerra R Hamzaoui J Marrakchi Z Hadjri S Chamakhi S Zidi B Ben Slama C Efficacy and safety of bromocriptine in the treatment of macroprolactinomas Ann Endocrinol 2002 63 524 531 12527854

[B77] Falsetti L Zanagnolo V Gastaldi A A retrospective study of idiopathic hyperprolactinemias Curr Ther Res Clin Exp 1988 43 1063 1072

[B78] Fletes Rabago VM Torres Farias S Dominguez Jimenez A Padilla Ruiz R [Alternative to bromocriptine (BEC) management in patients with prolactinoma and intolerance to oral BEC] Ginecol Obstet Mex 1991 59 283 288 1797615

[B79] Greenspan SL Oppenheim DS Klibanski A Importance of gonadal steroids to bone mass in men with hyperprolactinemic hypogonadism Ann Intern Med 1989 110 526 531 2923387 10.7326/0003-4819-110-7-526

[B80] Haase R Jaspers C Schulte HM Lancranja I Pfingsten H Orri-Fend M Reinwein D Benker G Control of prolactin-secreting macroadenomas with parenteral, long-acting bromocriptine in 30 patients treated for up to 3 years Clin Endocrinol 1993 38 165 176 10.1111/j.1365-2265.1993.tb00989.x 8435897

[B81] Holtkamp W Nagel GA Bromocriptine with chemotherapy resistant, metastatic breast cancer. Results of the AIO-Study GO-MC-BROMO 2/82 Onkologie 1988 11 121 127 10.1159/000216502 3045725

[B82] Jamrozik SI Bennet AP James-Deidier A Tremollieres F Saint-Martin F Dumoulin S Valat-Coustols M de Glisezinski I Tremoulet M Manelfe C Louvet JP Treatment with long acting repeatable bromocriptine (Parlodel-LAR*) in patients with macroprolactinomas: long-term study in 29 patients J Endocrinol Invest 1996 19 472 479 8884542 10.1007/BF03349893

[B83] Lengyel AM Mussio W Imamura P Vieira JG Lancranjan I Long-acting injectable bromocriptine (Parlodel LAR) in the chronic treatment of prolactin-secreting macroadenomas Fertil Steril 1993 59 980 987 8486199 10.1016/s0015-0282(16)55914-7

[B84] Lin SQ Experiences with bromocriptine treatment of female infertility due to hyperprolactinemia Zhonghua Fu Chan Ke Za Zhi 1992 27 28 31 1505275

[B85] Moberg E af Trampe E Wersall J Werner S Long-term effects of radiotherapy and bromocriptine treatment in patients with previous surgery for macroprolactinomas Neurosurgery 1991 29 200 204 discussion 204-205 10.1227/00006123-199108000-00005 1886657

[B86] Maraschini C Moro M Masala A Toja P Alagna S Brunani A Rovasio PP Ginanni A Lancranjan I Cavagnini F Chronic treatment with parlodel LAR of patients with prolactin-secreting tumours. Different responsiveness of micro- and macroprolactinomas Acta Endocrinol (Copenh) 1991 125 494 501 1759539 10.1530/acta.0.1250494

[B87] Merola B Colao A Caruso E Sarnacchiaro F Lancranjan I Lombardi G Schettini G Effectiveness and long-term tolerability of the slow release oral form of bromocriptine on tumoral and non-tumoral hyperprolactinemia J Endocrinol Invest 1992 15 173 176 1624676 10.1007/BF03348700

[B88] Molitch ME Elton RL Blackwell RE Caldwell B Chang RJ Jaffe R Joplin G Robbins RJ Tyson J Thorner MO Bromocriptine as primary therapy for prolactin-secreting macroadenomas: results of a prospective multicenter study J Clin Endocrinol Metab 1985 60 698 705 10.1210/jcem-60-4-698 3882737

[B89] Mornex R Orgiazzi J Hugues B Gagnaire JC Claustrat B Normal pregnancies after treatment of hyperprolactinemia with bromoergocryptine, despite suspected pituitary tumors J Clin Endocrinol Metab 1978 47 290 295 10.1210/jcem-47-2-290 400717

[B90] Moro M Maraschini C Toja P Masala A Alagna S Rovasio PP Ginanni A Lancranjan I Cavagnini F Comparison between a slow-release oral preparation of bromocriptine and regular bromocriptine in patients with hyperprolactinemia: a double blind, double dummy study Horm Res 1991 35 137 141 10.1159/000181889 1806467

[B91] Paoletti AM Cagnacci A Mais V Ajossa S Guerriero S Murgia C Depau GF Serra GG Melis GB Shrinkage of pituitary PRL-secernent adenoma after short-term treatment with bromocriptine long-acting repeatable injections Clin Exp Obstet Gynecol 1994 21 124 128 8070116

[B92] Rasmussen C Larsson SG Bergh T Long-term radiographic follow-up of the sella turcica in hyperprolactinaemic women Aust N Z J Obstet Gynaecol 1990 30 257 264 10.1111/j.1479-828X.1990.tb03228.x 2256866

[B93] Schettini G Lombardi G Merola B Colao A Miletto P Caruso E Lancranjan I Rapid and long-lasting suppression of prolactin secretion and shrinkage of prolactinomas after injection of long-acting repeatable form of bromocriptine (Parlodel LAR) Clin Endocrinol 1990 33 161 169 10.1111/j.1365-2265.1990.tb00479.x 2225475

[B94] Skrabanek P McDonald D De Valera E Plasma prolactin in amenorrhoea, infertility, and other disorders: A retrospective study of 608 patients Ir J Med Sci 1980 149 236 245 10.1007/BF02939147 7190555

[B95] Spark RF Baker R Bienfang DC Bergland R Bromocriptine reduces pituitary tumor size and hypersection. Requiem for pituitary surgery? JAMA 1982 247 311 316 10.1001/jama.1982.03320280031025 7054532

[B96] Thorner MO Besser GM Bromocriptine treatment of hyperprolactinaemic hypogonadism Acta Endocrinol Suppl 1978 216 131 146 347858

[B97] Tsagarakis S Tsiganou E Tzavara I Nikolou H Thalassinos N Effectiveness of a long-acting injectable form of bromocriptine in patients with prolactin and growth hormone secreting macroadenomas Clin Endocrinol 1995 42 593 599 10.1111/j.1365-2265.1995.tb02685.x 7634499

[B98] van 't Verlaat JW Croughs RJ Hendriks MJ Bosma NJ Nortier JW Thijssen JH Bromocriptine treatment of prolactin secreting macroadenomas: a radiological, ophthalmological and endocrinological study Acta Endocrinol (Copenh) 1986 112 487 493 3751462 10.1530/acta.0.1120487

[B99] Walsh JP Pullan PT Hyperprolactinaemia in males: a heterogeneous disorder Aust N Z J Med 1997 27 385 390 10.1111/j.1445-5994.1997.tb02196.x 9448878

[B100] Wass JA Williams J Charlesworth M Kingsley DP Halliday AM Doniach I Rees LH McDonald WI Besser GM Bromocriptine in management of large pituitary tumours Br Med J (Clin Res Ed) 1982 284 1908 1911 10.1136/bmj.284.6333.1908 PMC1498802 6805756

[B101] Weingrill CO Mussio W Moraes CR Portes E Castro RC Lengyel AM Long-acting oral bromocriptine (Parlodel SRO) in the treatment of hyperprolactinemia Fertil Steril 1992 57 331 335 1735484 10.1016/s0015-0282(16)54840-7

[B102] Yang MS Hong JW Lee SK Lee EJ Kim SH Clinical management and outcome of 36 invasive prolactinomas treated with dopamine agonist J Neurooncol 2011 104 195 204 10.1007/s11060-010-0459-3 21107645

[B103] Bhansali A Walia R Dutta P Khandelwal N Sialy R Bhadada S Efficacy of cabergoline on rapid escalation of dose in men with macroprolactinomas Indian J Med Res 2010 131 530 535 20424304

[B104] Biller BMK Molitch ME Vance ML Cannistraro KB Davis KR Simons JA Schoenfelder JR Klibanski A Treatment of prolactin-secreting macroadenomas with the once-weekly dopamine agonist cabergoline J Clin Endocrinol Metab 1996 81 2338 2343 10.1210/jc.81.6.2338 8964874

[B105] Bolko P Jaskula M Wasko R Wolun M Sowinski J The assessment of cabergoline efficacy and tolerability in patients with pituitary prolactinoma type Polskie Archiwum Medycyny Wewnetrznej 2003 109 489 495 14768178

[B106] Buyukbayrak EE Karageyim Karsidag AY Kars B Balcik O Pirimoglu M Unal O Turan C Effectiveness of short-term maintenance treatment with cabergoline in microadenoma-related and idiopathic hyperprolactinemia Arch Gynecol Obstet 2010 282 561 566 10.1007/s00404-010-1562-6 20571820

[B107] Cho E-H Lee SA Chung JY Koh EH Cho YH Kim JH Kim CJ Kim M-S Efficacy and safety of cabergoline as first line treatment for invasive giant prolactinoma J Korean Med Sci 2009 24 874 878 10.3346/jkms.2009.24.5.874 19794986 PMC2752771

[B108] Ciccarelli E Giusti M Miola C Potenzoni F Sghedoni D Camanni F Giordano G Effectiveness and tolerability of long term treatment with cabergoline, a new long-lasting ergoline derivative, in hyperprolactinemic patients J Clin Endocrinol Metab 1989 69 725 728 10.1210/jcem-69-4-725 2570790

[B109] Colao A Di Sarno A Landi ML Cirillo S Sarnacchiaro F Facciolli G Pivonello R Cataldi M Merola B Annunziato L Lombardi G Long-term and low-dose treatment with cabergoline induces macroprolactinoma shrinkage J Clin Endocrinol Metab 1997 82 3574 3579 10.1210/jc.82.11.3574 9360509

[B110] Colao A Di Sarno A Landi ML Scavuzzo F Cappabianca P Pivonello R Volpe R Di Salle F Cirillo S Annunziato L Lombardi G Macroprolactinoma shrinkage during cabergoline treatment is greater in naive patients than in patients pretreated with other dopamine agonists: a prospective study in 110 patients J Clin Endocrinol Metab 2000 85 2247 2252 10.1210/jc.85.6.2247 10852458

[B111] Colao A Vitale G Di Sarno A Spiezia S Guerra E Ciccarelli A Lombardi G Prolactin and prostate hypertrophy: a pilot observational, prospective, case-control study in men with prolactinoma J Clin Endocrinol Metab 2004 89 2770 2775 10.1210/jc.2003-032055 15181056

[B112] Colao A Vitale G Cappabianca P Briganti F Ciccarelli A De Rosa M Zarrilli S Lombardi G Outcome of cabergoline treatment in men with prolactinoma: effects of a 24-month treatment on prolactin levels, tumor mass, recovery of pituitary function, and semen analysis J Clin Endocrinol Metab 2004 89 1704 1711 10.1210/jc.2003-030979 15070934

[B113] Corsello SM Ubertini G Altomare M Lovicu RM Migneco MG Rota CA Colosimo C Giant prolactinomas in men: Efficacy of cabergoline treatment Clin Endocrinol 2003 58 662 670 10.1046/j.1365-2265.2003.01770.x 12699451

[B114] De Bellis A Colao A Savoia A Coronella C Pasquali D Conte M Pivonello R Bellastella A Sinisi AA Bizzarro A Lombardi G Bellastella G Effect of long-term cabergoline therapy on the immunological pattern and pituitary function of patients with idiopathic hyperprolactinaemia positive for antipituitary antibodies Clin Endocrinol 2008 69 285 291 10.1111/j.1365-2265.2008.03200.x 18221394

[B115] De Rosa M Ciccarelli A Zarrilli S Guerra E Gaccione M Di Sarno A Lombardi G Colao A The treatment with cabergoline for 24 month normalizes the quality of seminal fluid in hyperprolactinaemic males Clin Endocrinol 2006 64 307 313 10.1111/j.1365-2265.2006.02461.x 16487441

[B116] Delgrange E Maiter D Donckier J Effects of the dopamine agonist cabergoline in patients with prolactinoma intolerant or resistant to bromocriptine Eur 1996 134 454 456 10.1530/eje.0.1340454 8640297

[B117] Ferrari C Paracchi A Mattei AM De Vincentiis S D'Alberton A Crosignani P Cabergoline in the long-term therapy of hyperprolactinemic disorders Acta Endocrinol (Copenh) 1992 126 489 494 1642081 10.1530/acta.0.1260489

[B118] Ferrari CI Abs R Bevan JS Brabant G Ciccarelli E Motta T Mucci M Muratori M Musatti L Verbessem G Scanlon MF Treatment of macroprolactinoma with cabergoline: a study of 85 patients Clin Endocrinol 1997 46 409 413 10.1046/j.1365-2265.1997.1300952.x 9196602

[B119] Ono M Miki N Kawamata T Makino R Amano K Seki T Kubo O Hori T Takano K Prospective study of high-dose cabergoline treatment of prolactinomas in 150 patients.[see comment] J Clin Endocrinol Metab 2008 93 4721 4727 10.1210/jc.2007-2758 18812485

[B120] Ono M Miki N Amano K Kawamata T Seki T Makino R Takano K Izumi S-Okada Y Hori T Individualized high-dose cabergoline therapy for hyperprolactinemic infertility in women with micro- and macroprolactinomas J Clin Endocrinol Metab 2010 95 2672 2679 10.1210/jc.2009-2605 20357175

[B121] Pontikides N Krassas GE Nikopoulou E Kaltsas T Cabergoline as a first-line treatment in newly diagnosed macroprolactinomas Pituitary 2000 2 277 281 10.1023/A:1009913200542 11081149

[B122] Raverot G Jacob M Jouanneau E Delemer B Vighetto A Pugeat M Borson-Chazot F Secondary deterioration of visual field during cabergoline treatment for macroprolactinoma Clin Endocrinol 2009 70 588 592 10.1111/j.1365-2265.2008.03364.x 18673461

[B123] Shimon I Benbassat C Hadani M Effectiveness of long-term cabergoline treatment for giant prolactinoma: study of 12 men Eur 2007 156 225 231 10.1530/EJE-06-0646 17287412

[B124] Sinha S Sharma BS Mahapatra AK Microsurgical management of prolactinomas - clinical and hormonal outcome in a series of 172 cases Neurol India 2011 59 532 536 10.4103/0028-3886.84332 21891928

[B125] Walia R Bhansali A Dutta P Khandelwal N Sialy R Bhadada S Recovery pattern of hypothalamo-pituitary-testicular axis in patients with macroprolactinomas after treatment with cabergoline Indian J Med Res 2011 134 314 319 21985814 PMC3193712

[B126] Webster J Piscitelli G Polli A D'Alberton A Falsetti L Ferrari C Fioretti P Giordano G L'Hermite M Ciccarelli E The efficacy and tolerability of long-term cabergoline therapy in hyperprolactinaemic disorders: an open, uncontrolled, multicentre study. European Multicentre Cabergoline Study Group Clin Endocrinol 1993 39 323 329 10.1111/j.1365-2265.1993.tb02372.x 7900937

[B127] Colao A De Rosa M Sarnacchiaro F Di Sarno A Landi ML Iervolino E Zarrilli S Merola B Lombardi G Chronic treatment with CV 205-502 restores the gonadal function in hyperprolactinemic males Eur 1996 135 548 552 10.1530/eje.0.1350548 8980156

[B128] Duranteau L Chanson P Lavoinne A Horlait S Lubetzki J Kuhn JM Effect of the new dopaminergic agonist CV 205-502 on plasma prolactin levels and tumour size in bromocriptine-resistant prolactinomas Clin Endocrinol 1991 34 25 29 10.1111/j.1365-2265.1991.tb01731.x 1672268

[B129] Kvistborg A Halse J Bakke S Bjoro T Hansen E Djoseland O Brownell J Jervell J Long-term treatment of macroprolactinomas with CV 205-502 Acta Endocrinol (Copenh) 1993 128 301 307 8098891 10.1530/acta.0.1280301

[B130] Merola B Sarnacchiaro F Colao A Disomma C Disarno A Landi ML Marzullo P Panza N Battista C Lombardi G CV-205-502 in the treatment of tumoral and nontumoral hyperprolactinemic states Biomed Pharmacother 1994 48 167 174 10.1016/0753-3322(94)90105-8 7993981

[B131] Morange I Barlier A Pellegrini I Brue T Enjalbert A Jaquet P Prolactinomas resistant to bromocriptine: Long-term efficacy of quinagolide and outcome of pregnancy Eur 1996 135 413 420 10.1530/eje.0.1350413 8921822

[B132] Newman CB Hurley AM Kleinberg DL Effect of CV 205-502 in hyperprolactinaemic patients intolerant of bromocriptine Clin Endocrinol 1989 31 391 400 10.1111/j.1365-2265.1989.tb01263.x 2576397

[B133] Nickelsen T Jungmann E Althoff P Schummdraeger PM Usadel KH Treatment of macroprolactinoma with the new potent nonergot D2-dopamine agonist quinagolide and effects on prolactin levels, pituitary-function, and the renin-aldosterone system- results of a clinical long-term study Arzneimittelforschung 1993 43 421 425 8098604

[B134] Rasmussen C Bergh T Wide L Brownell J Long-term treatment with a new non-ergot long-acting dopamine agonist, CV 205-502, in women with hyperprolactinaemia Clin Endocrinol 1988 29 271 279 10.1111/j.1365-2265.1988.tb01225.x 2908030

[B135] Rohmer V Freneau E Morange I Simonetta C Efficacy of quinagolide in resistance to dopamine agonists: results of a multicenter study. Club de l'Hypophyse Ann Endocrinol (Paris) 2000 61 411 417 11084391

[B136] Shoham Z Homburg R Jacobs HS CV 205–502–effectiveness, tolerability, and safety over 24-month study Fertil Steril 1991 55 501 506 1672113 10.1016/s0015-0282(16)54175-2

[B137] van der Heijden PF Lappohn RE Corbey RS de Goeij WB Brownell J Rolland R The effectiveness, safety, and tolerability of CV 205-502 in hyperprolactinemic women: a 12-month study Fertil Steril 1989 52 574 579 2806597 10.1016/s0015-0282(16)60966-4

[B138] van der Lely AJ Brownell J Lamberts SW The efficacy and tolerability of CV 205-502 (a nonergot dopaminergic drug) in macroprolactinoma patients and in prolactinoma patients intolerant to bromocriptine J Clin Endocrinol Metab 1991 72 1136 1141 10.1210/jcem-72-5-1136 1673685

[B139] Vance ML Lipper M Klibanski A Biller BMK Samaan NA Molitch ME Treatment of prolactin-secreting pituitary macroadenomas with the long-acting non-ergot dopamine agonist CV 205-502 Ann Intern Med 1990 112 668 673 1970714 10.7326/0003-4819-112-9-668

[B140] Vilar L Burke CW Quinagolide efficacy and tolerability in hyperprolactinaemic patients who are resistant to or intolerant of bromocriptine.[see comment] Clin Endocrinol 1994 41 821 826 10.1111/j.1365-2265.1994.tb02799.x 7889620

[B141] Freda PU Andreadis CI Khandji AG Khoury M Bruce JN Jacobs TP Wardlaw SL Long-term treatment of prolactin-secreting macroadenomas with pergolide J Clin Endocrinol Metab 2000 85 8 13 10.1210/jc.85.1.8 10634356

[B142] Jaspers C Benker G Reinwein D Treatment of prolactinoma patients with the new non-ergot dopamine agonist roxindol: First results Clin Investig 1994 72 451 456 10.1007/BF00180520 7950157

[B143] Orrego JJ Chandler WF Barkan AL Pergolide as primary therapy for macroprolactinomas Pituitary 2000 3 251 256 10.1023/A:1012836331506 11788013

[B144] Sibal L Ugwu P Kendall-Taylor P Ball SG James RA Pearce SH Hall K Quinton R Medical therapy of macroprolactinomas in males: I. Prevalence of hypopituitarism at diagnosis. II. Proportion of cases exhibiting recovery of pituitary function Pituitary 2002 5 243 246 10.1023/A:1025377816769 14558672

[B145] Verde G Chiodini PG Liuzzi A Cozzi R Favales F Botalla L Spelta B Dalla Bonzana D Rainer E Horowski R Effectiveness of the dopamine agonist lisuride in the treatment of acromegaly and pathological hyperprolactinemic states J Endocrinol Invest 1980 3 405 414 6782153 10.1007/BF03349379

[B146] Jezkova J Hana V Krsek M Weiss V Vladyka V Liscak R Vymazal J Pecen L Marek J Use of the Leksell gamma knife in the treatment of prolactinoma patients Clin Endocrinol 2009 70 732 741 10.1111/j.1365-2265.2008.03384.x 18710463

[B147] Pan L Zhang N Wang EM Wang BJ Dai JZ Cai PW Gamma knife radiosurgery as a primary treatment for prolactinomas J Neurosurg 2000 93 10 13 11143223 10.3171/jns.2000.93.supplement

[B148] Pouratian N Sheehan J Jagannathan J Laws ER Steiner L Vance ML Gamma knife radiosurgery for medically and surgically refractory prolactinomas Neurosurgery 2006 59 255 266 discussion 255-266 10.1227/01.NEU.0000223445.22938.BD 16883166

[B149] Sun DQ Cheng JJ Frazier JL Batra S Wand G Kleinberg LR Rigamonti D Quinones-Hinojosa A Salvatori R Lim M Treatment of pituitary adenomas using radiosurgery and radiotherapy: a single center experience and review of literature Neurosurg Rev 2010 34 181 189 20838838 10.1007/s10143-010-0285-2

[B150] Tsagarakis S Grossman A Plowman PN Jones AE Touzel R Rees LH Wass JA Besser GM Megavoltage pituitary irradiation in the management of prolactinomas: long-term follow-up Clin Endocrinol 1991 34 399 406 10.1111/j.1365-2265.1991.tb00312.x 1647898

[B151] Yoon SC Suh TS Jang HS Chung SM Kim YS Ryu MR Choi KH Son HY Kim MC Shinn KS Clinical results of 24 pituitary macroadenomas with linac-based stereotactic radiosurgery Int J Radiat Oncol Biol Phys 1998 41 849 853 10.1016/S0360-3016(98)00124-2 9652848

[B152] Zhang N Pan L Dai J Wang B Wang E Zhang W Cai P Gamma Knife radiosurgery as a primary surgical treatment for hypersecreting pituitary adenomas Stereotact Funct Neurosurg 2000 75 123 128 10.1159/000048393 11740180

[B153] Zierhut D Flentje M Adolph J Erdmann J Raue F Wannenmacher M External Radiotherapy of Pituitary Adenomas Int J Radiat Oncol Biol Phys 1995 33 307 314 10.1016/0360-3016(95)00071-6 7673017

[B154] Kelly WF Mashiter K Doyle FH Banks LM Joplin GF Treatment of prolactin-secreting pituitary tumours in young women by needle implantation of radioactive yttrium Q J Med 1978 47 473 493 108759

[B155] Littley MD Shalet SM Reid H Beardwell CG Sutton ML The effect of external pituitary irradiation on elevated serum prolactin levels in patients with pituitary macroadenomas Q J Med 1991 81 985 998 10.1093/qjmed/81.3.985 1808643

[B156] Rudoler SB Ruffer JE Gennarelli TA Savino PJ Fowble BF Long-term results of radiotherapy for prolactin-secreting pituitary macroadenomas Radiat Oncol Investig 1996 4 185 191 10.1002/(SICI)1520-6823(1996)4:4<185::AID-ROI6>3.0.CO;2-U

[B157] Tanaka S Link MJ Brown PD Stafford SL Young WF Pollock BE Gamma knife radiosurgery for patients with prolactin-secreting pituitary adenomas World Neurosurg 2010 74 147 152 10.1016/j.wneu.2010.05.007 21300006

[B158] Amar AP Couldwell WT Chen JCT Weiss MH Predictive value of serum prolactin levels measured immediately after transsphenoidal surgery J Neurosurg 2002 97 307 314 10.3171/jns.2002.97.2.0307 12186458

[B159] Babey M Sahli R Vajtai I Andres RH Seiler RW Pituitary surgery for small prolactinomas as an alternative to treatment with dopamine agonists Pituitary 2011 14 222 230 10.1007/s11102-010-0283-y 21170594 PMC3146980

[B160] Barbarino A De Marinis L Anile C Menini E Merlini G Maira G Dopaminergic mechanisms regulating prolactin secretion in patients with prolactin-secreting pituitary adenoma. Long-term studies after selective transsphenoidal surgery Metabolism 1982 31 1100 1104 10.1016/0026-0495(82)90159-7 6813636

[B161] Charpentier G de Plunkett T Jedynak P Peillon F Le Gentil P Racadot J Visot A Derome P Surgical treatment of prolactinomas. Short- and long-term results, prognostic factors Horm Res 1985 22 222 227 10.1159/000180098 4054842

[B162] Dusick JR Fatemi N Mattozo C McArthur D Cohan P Wang C Swerdloff RS Kelly DF Pituitary function after endonasal surgery for nonadenomatous parasellar tumors: Rathke's cleft cysts, craniopharyngiomas, and meningiomas Surg Neurol 2008 70 482 490 10.1016/j.surneu.2008.03.027 18482750

[B163] Fatemi N Dusick JR Mattozo C McArthur DL Cohan P Boscardin J Wang C Swerdloff RS Kelly DF Pituitary hormonal loss and recovery after transsphenoidal adenoma removal Neurosurgery 2008 63 709 718 10.1227/01.NEU.0000325725.77132.90 18981881

[B164] Feigenbaum SL Downey DE Wilson CB Jaffe RB Transsphenoidal pituitary resection for preoperative diagnosis of prolactin-secreting pituitary adenoma in women: long term follow-up J Clin Endocrinol Metab 1996 81 1711 1719 10.1210/jc.81.5.1711 8626821

[B165] Gokalp HZ Deda H Attar A Ugur HC Arasil E Egemen N The neurosurgical management of prolactinomas J Neurosurg Sci 2000 44 128 132 11126446

[B166] Guidetti B Fraioli B Cantore GP Results of surgical management of 319 pituitary adenomas Acta Neurochir 1987 85 117 124 10.1007/BF01456107 3591473

[B167] Hamilton DK Vance ML Boulos PT Laws ER Surgical outcomes in hyporesponsive prolactinomas: analysis of patients with resistance or intolerance to dopamine agonists Pituitary 2005 8 53 60 10.1007/s11102-005-5086-1 16411069

[B168] Hirohata T Uozumi T Mukada K Arita K Kurisu K Yano T Takechi A Onda J Influence of pregnancy on the serum prolactin level following prolactinoma surgery Acta Endocrinol (Copenh) 1991 125 259 267 1950339 10.1530/acta.0.1250259

[B169] Laws ER Fode NC Redmond MJ Transsphenoidal surgery following unsuccessful prior therapy. An assessment of benefits and risks in 158 patients J Neurosurg 1985 63 823 829 10.3171/jns.1985.63.6.0823 2997414

[B170] Maira G Anile C De Marinis L Barbarino A Prolactin-secreting adenomas: Surgical results and long-term follow-up Neurosurgery 1989 24 736 743 10.1227/00006123-198905000-00013 2716983

[B171] Massoud F Serri O Hardy J Somma M Beauregard H Transsphenoidal adenomectomy for microprolactinomas: 10 to 20 years of follow-up Surg Neurol 1996 45 341 346 10.1016/0090-3019(95)00430-0 8607082

[B172] Nakagawa H Iwatsuki K Yamada M Hagiwara Y Moriuchi S Kadota T Latent prolactinoma on MRI-selective venous sampling and trans-sphenoidal microsurgical treatment Neurol Res 2001 23 691 696 10.1179/016164101101199199 11680507

[B173] Pandey P Ojha BK Mahapatra AK Pediatric pituitary adenoma: A series of 42 patients J Clin Neurosci 2005 12 124 127 10.1016/j.jocn.2004.10.003 15749410

[B174] Qu X Wang M Wang GD Han T Mou CZ Han LZ Jiang M Qu YM Zhang MA Pang Q Xu GM Surgical outcomes and prognostic factors of transsphenoidal surgery for prolactinoma in men: a single-center experience with 87 consecutive cases Eur 2011 164 499 504 10.1530/EJE-10-0961 21252173

[B175] Rawe SE Williamson HO Levine JH Phansey SA Hungerford D Adkins WY Prolactinomas: surgical therapy, indications and results Surg Neurol 1980 14 161 167 7434180

[B176] Saitoh Y Mori S Arita N Nagatani M Hayakawa T Koizumi K Tanizawa O Uozumi T Mogami H Treatment of prolactinoma based on the results of transsphenoidal operations Surg Neurol 1986 26 338 344 10.1016/0090-3019(86)90133-3 3750191

[B177] Santoro A Minniti G Ruggeri A Esposito V Jaffrain-Rea ML Delfini R Biochemical remission and recurrence rate of secreting pituitary adenomas after transsphenoidal adenomectomy: long-term endocrinologic follow-up results Surg Neurol 2007 68 513 518 10.1016/j.surneu.2007.05.057 17961741

[B178] Scamoni C Balzarini C Crivelli G Dorizzi A Treatment and long-term follow-up results of prolactin secreting pituitary adenomas J Neurosurg Sci 1991 35 9 16 1890464

[B179] Shen CC Wang YC Wei-Shan H Chang CS Sun MH Endoscopic endonasal transsphenoidal surgery for pituitary tumors Chinese Med J (Taipei) 2000 63 301 310 10820909

[B180] Soule SG Farhi J Conway GS Jacobs HS Powell M The outcome of hypophysectomy for prolactinomas in the era of dopamine agonist therapy.[see comment] Clin Endocrinol 1996 44 711 716 10.1046/j.1365-2265.1996.738559.x 8759184

[B181] Webster J Page MD Bevan JS Richards SH Douglas-Jones AG Scanlon MF Low recurrence rate after partial hypophysectomy for prolactinoma: the predictive value of dynamic prolactin function tests Clin Endocrinol 1992 36 35 44 10.1111/j.1365-2265.1992.tb02900.x 1559298

[B182] Wolfsberger S Czech T Vierhapper H Benavente R Knosp E Microprolactinomas in males treated by transsphenoidal surgery Acta Neurochir 2003 145 935 940 discussion 940-931 10.1007/s00701-003-0134-y 14628197

[B183] Zhang HW Sun W Yang J Yan CX Yu CJ Diagnosis and treatment of pituitary microadenoma: report of 80 cases Neurol Res 2008 30 587 593 10.1179/174313208X310287 18647498

[B184] Jamjoom ZAB Malabarey T Jamjoom AHB Sulimani R Rahman NU Sadiq S Problems in the management of large prolactin-secreting pituitary adenomas Saudi Med J 1995 16 119 125

[B185] Saeki N Nakamura M Sunami K Yamaura A Surgical indication after bromocriptine therapy on giant prolactinomas: Effects and limitations of the medical treatment Endocr J 1998 45 529 537 10.1507/endocrj.45.529 9881903

[B186] Thompson CJ Tam NNC Joyce JM Leav I Ho SM Gene expression profiling of testosterone and estradiol-17 beta-induced prostatic dysplasia in noble rats and response to the antiestrogen ICI 182,780 Endocrinology 2002 143 2093 2105 10.1210/en.143.6.2093 12021174

[B187] Thomson JA Davies DL McLaren EH Teasdale GM Ten year follow up of microprolactinoma treated by transsphenoidal surgery Br Med J 1994 309 1409 1410 10.1136/bmj.309.6966.1409 7819849 PMC2541328

[B188] Oruckaptan HH Senmevsim O Ozcan OE Ozgen T Pituitary adenomas: results of 684 surgically treated patients and review of the literature Surg Neurol 2000 53 211 219 10.1016/S0090-3019(00)00171-3 10773251

[B189] Rush S Cooper PR Symptom resolution, tumor control, and side effects following postoperative radiotherapy for pituitary macroadenomas Int J Radiat Oncol Biol Phys 1997 37 1031 1034 10.1016/S0360-3016(96)00586-X 9169809

[B190] Badawy SZ Marziale JC Rosenbaum AE Chang JK Joy SE The long-term effects of pregnancy and bromocriptine treatment on prolactinomas–the value of radiologic studies Early Pregnancy 1997 3 306 311 10086082

[B191] Berinder K Hulting AL Granath F Hirschberg AL Akre O Parity, pregnancy and neonatal outcomes in women treated for hyperprolactinaemia compared with a control group Clin Endocrinol 2007 67 393 397 10.1111/j.1365-2265.2007.02897.x 17561983

[B192] Colao A Abs R Barcena DG Chanson P Paulus W Kleinberg DL Pregnancy outcomes following cabergoline treatment: extended results from a 12-year observational study Clin Endocrinol 2008 68 66 71 10.1111/j.1365-2265.2007.03000.x 17760883

[B193] Cristiani P De March A Bulletti C Follow-up of 17 hyperprolactinemic pregnant women by plasma prolactin levels and pituitary politomograms J Gynaecol Endocrinol 1985 1 48 51

[B194] Crosignani PG Mattei AM Scarduelli C Cavioni V Boracchi P Is pregnancy the best treatment for hyperprolactinaemia? Hum Reprod 1989 4 910 912 2575622 10.1093/oxfordjournals.humrep.a137010

[B195] Crosignani PG Mattei AM Severini V Cavioni V Maggioni P Testa G Long-term effects of time, medical treatment and pregnancy in 176 hyperprolactinemic women Eur J Obstet Gynecol Reprod Biol 1992 44 175 180 10.1016/0028-2243(92)90094-F 1607056

[B196] Godo G Koloszar S Szilagyi I Daru J Sas M Experience relating to pregnancy, lactation, and the afterweaning condition of hyperprolactinemic patients treated with bromocriptine Fertil Steril 1989 51 529 531 2920853 10.1016/s0015-0282(16)60569-1

[B197] Holmgren U Bergstrand G Hagenfeldt K Werner S Women with prolactinoma–effect of pregnancy and lactation on serum prolactin and on tumour growth Acta Endocrinol (Copenh) 1986 111 452 459 3705884 10.1530/acta.0.1110452

[B198] Jeffcoate WJ Pound N Sturrock ND Lambourne J Long-term follow-up of patients with hyperprolactinaemia. [see comment] Clin Endocrinol (Oxf) 1996 45 299 303 10.1046/j.1365-2265.1996.00824.x 8949567

[B199] Karunakaran S Page RC Wass JA The effect of the menopause on prolactin levels in patients with hyperprolactinaemia Clin Endocrinol (Oxf) 2001 54 295 300 10.1046/j.1365-2265.2001.01190.x 11298080

[B200] Kelly WF Doyle FH Mashiter K Banks LM Gordon H Joplin GF Pregnancies in women with hyperprolactinaemia: clinical course and obstetric complications of 41 pregnancies in 27 women Br J Obstet Gynaecol 1979 86 698 705 10.1111/j.1471-0528.1979.tb11269.x 497142

[B201] Kupersmith MJ Rosenberg C Kleinberg D Visual loss in pregnant women with pituitary adenomas.[see comment] Ann Intern Med 1994 121 473 477 8067644 10.7326/0003-4819-121-7-199410010-00001

[B202] Rasmussen C Bergh T Nillius SJ Wide L Return of menstruation and normalization of prolactin in hyperprolactinemic women with bromocriptine-induced pregnancy Fertil Steril 1985 44 31 34 4007192 10.1016/s0015-0282(16)48673-5

[B203] Rossi AM Vilska S Heinonen PK Outcome of pregnancies in women with treated or untreated hyperprolactinemia Eur J Obstet Gynecol Reprod Biol 1995 63 143 146 10.1016/0301-2115(95)02257-0 8903770

[B204] Woodhouse NJ Niles N McDonald D McCorkell S Prolactin levels in pregnancy: comparison of normal subjects with patients having micro- or macroadenomas after early bromocriptine withdrawal Horm Res 1985 21 1 9 10.1159/000180019 3972334

[B205] Zarate A Canales ES Alger M Forsbach G The effect of pregnancy and lactation on pituitary prolactin-secreting tumours Acta Endocrinol 1979 92 407 412 517046 10.1530/acta.0.0920407

