# Supplementary material for: Treatment of hyperprolactinemia: a systematic review and meta-analysis
Source: Syst Rev. 2012 Jul 24;1:33. doi: 10.1186/2046-4053-1-33 (PMC3483691; doi:10.1186/2046-4053-1-33)
Supplement: Additional file 1 — Appendix. Treatment of hyperprolactinemia: a systematic review and meta-analysis. Description of data: Contents: Baseline characteristics of the included comparative studies (Supplemental Table 1), Quality of the included observational comparative studies (Supplemental Table 2), Quality of the included observational dopamine withdrawal studies (Supplemental Table 3), Quality of randomized trials (Supplemental Table 4), Summary of uncontrolled studies of dopamine agonists (Supplemental Tables 5A-E), Meta-analyses figures (Supplemental Figures 1A-5B), Subgroup analyses (Supplemental Tables 6A-D), Summary of uncontrolled studies of radiotherapy, surgery, combinations of treatment, and pregnancy (Supplemental Tables 7A-F), References, Search strategy [26-205]. [file 2046-4053-1-33-S1.pdf]

Treatment of hyperprolactinemia: a systematic review and meta-analysis

Appendix

Contents

1. Baseline characteristics of the included comparative studies (Supplemental Table 1)
2. Quality of the included observational comparative studies (Supplemental Table 2)
3. Quality of the included observational dopamine withdrawal studies (Supplemental Table 3)
4. Quality of randomized trials (Supplemental Table 4)
5. Summary of uncontrolled studies of dopamine agonists (Supplemental Tables 5A-E)
6. Meta-analyses figures (Supplemental Figures 1A-5B)
7. Subgroup analyses (Supplemental Tables 6A-D)
8. Summary of uncontrolled studies of radiotherapy, surgery, combinations of treatment, and pregnancy (Supplemental Tables 7A-F)
9. References
10. Search strategy

Supplemental Table 1: Baseline characteristics of the included comparative studies

**Author, Year**	**Sample Size**	**Study Design**	**Description of Patients**	**Age** (yrs +/-SD unless noted otherwise)	**Intervention**	**Duration of Intervention** (months)
Asano, 2001[1]	13	Retrospective cohort	Men with pure prolactinomas	37.3	8 men underwent transsphenoidal surgery followed by bromocriptine administration;<br>5 received bromocriptine or terguride alone	NR
Bahceci, 2010[2]	239	Retrospective cohort	Infertile patients with otherwise asymptomatic hyperprolactinemia	32.0	122 treated with cabergoline (1mg/wk in two divide doses) and 117 treated with bromocriptine (5mg/day) during controlled ovarian hyperstimulation	NR
Brue, 1992 [3]	27	Retrospective cohort	Hyperprolactinemic bromocriptine resistant patients	29	Patients were treated with dopamine agonist CV205-502 alone (doses between 0.15 to 0.525mg daily) or with surgery	NR
Candrina, 1987 [4]	21	Prospective cohort	Patients with macroprolactinomas undergoing surgery	44+/-8	13 patients were operated by transsphenoidal approach, 8 patients combined TSS with TCS or only TCS. After intervention 6 patients were immediately given DA, the other 15 were only given DA if evidence of relapse.	NR
Colao, 1995 [5]	34	Prospective cohort	Patients with prolactinomas	Range: 18-54	10 patients received BRC-SRO daily, dose of 5-20 mg for 1-24 months; 8 patients received BRC-LAR monthly, dose of 50-100 mg for 6-24 months; 16 patients received CV 205-502 daily, dose of 0.075-0.6 mg for 6-12 months	NR
De Rosa, 1998 [6]	17	Retrospective cohort	Men with macroprolactinoma	Range: 22-38	Cabergoline at 0.5mg once weekly for 15 days, then 0.5mg twice weekly, then titrated by PRL level versus Bromocriptine at 1.25mg twice daily for 1 week. then to 2.5mg twice daily for 3 weeks, then titrated by PRL level	6
Di Sarno, 2001 [7]	207	Retrospective cohort	Consecutive de novo patients with hyperprolactinemia	29	Micro- 64 patients- Cabergoline 0.25mg once weekly x 1 week, twice weekly x 1 week, then 0.5 mg twice weekly, then titrated based on PRL level; Macro-56 patients- Cabergoline 0.5mg weekly x 1 week, then twice weekly, then titrated based on PRL level. 87 patients- Bromocriptine 2.5 mg q.P.M. x 2 weeks then to 5 mg after lunch and 2.5 mg q.P.M., then titrated based on PRL level	24
Di Somma, 1998 [8]	20	Prospective cohort	Consecutive hyperprolactinemic men	36.7	Group 1 (6 patients) treated with bromocriptine dose 2.5 to 10mg 2 to 3 times daily; group 2 (7 patients) treated with quinagolide dose 0.075 to 0.3mg 1 or 2 times daily; group 3 (7 patients) dose 0.5 to 1.5mg once or twice a week	18
Hildebrandt, 1992[9]	36	Retrospective cohort	Patients with immunohistochemically proven PRL secreting micro or macroadenomas	33+/-15	Group A: 14 patients-single dose of a long-acting bromocriptine followed by chronic oral dopamine agonist therapy; group B: 12 patients underwent surgical excision first and if hyperprolactinemia persisted then were started on oral dopamine therapy. Group C; 10 patients received DA and then surgery (additional DA after surgery if needed)	1-6
Hirahara, 1998[10]	48	Randomized controlled trial	Hyperprolactinemic women with history of	Range: 20-40	24 with occult hyperprolactinemia and 24 with hyperprolactinemia were randomly assigned to bromocriptine	12

			recurrent spontaneous abortion not associated with other etiologies		(2.5mg to 5mg/day until the end of 9th week of gestation) vs. no treatment	
Homburg, 1990[11]	22	Randomized controlled trial	Women with persistent hyperprolactinemia (microprolactinoma or macroprolactinoma)	33	Patients were randomized to either Bromocriptine (2.5mg/day up to 10mg/day) or CV-205-502 (0.075mg/day up to 0.15mg/day).	6
Jeffcoate, 1996[12]	10	Retrospective cohort	Women with confirmed microprolactinoma	29.1	Intermittent treatment with DA, 60 received DA, 10 did not	NR
Lappohn, 1992[13]	24	Randomized controlled trial	Women with hyperprolactinemia	35	CV 205-502 0.025 mg/d then increased to 0.075 mg at bedtime vs. bromocriptine 1.25 mg/d, then increased to 2.5 mg twice daily	6
Mahmood, 2010[14]	100	Prospective cohort	Hyperprolactinemic women	28.1	50 women received cabergoline at 0.5 mg/week vs. 50 women who received bromocriptine up to a maximum of 2.5 mg twice daily	8
Mattei, 1991[15]	176	Retrospective cohort	Hyperprolactinemic women treated medically, pregnancy or “just waiting”	NR	107 women (46 with normal sella and 61 with prolactinoma) were treated with dopamine agonist (bromocriptine, metergoline, lisuride, dihydroergocristine, dihydroergocryptine and cabergoline)	Normal sella-12.6 months; Prolactinoma-11 months
Motazedian, 2010[16]	183	Randomized controlled trial	Hyperprolactinemic infertile women undergoing ovulation induction for intrauterine insemination	28.9	94 women received bromocriptine 2.5 mg twice daily vs. 89 women given cabergoline 0.25 mg twice weekly	NR
Pascal-Vigneron, 1995[17]	120	Randomized controlled trial	Hyperprolactinemic women with amenorrhea at 21 French centers	32	Cabergoline 0.5-1mg twice weekly vs. bromocriptine 2.5-5mg twice daily	6
Perrin, 1991[18]	40	Retrospective cohort	Patients with prolactinomas and elevated prolactin levels	33.8	20 patients with bromocriptine preoperatively 2.5 to 7.5 mg daily dose then transsphenoidal resection; 20 patients resection alone	BCT: Micro 15 months; Macro 5 months
Pinzone, 2000[19]	46	Retrospective cohort	Men with prolactinomas	49 micro; 46 macro	Bromocriptine, quinagolide, and/or cabergoline were administered as medical therapy.	NR
Rush, 1991[20]	20	Retrospective cohort	Hyperprolactinemic patients PRL >200ng/ml, tumor size >2cm	Range: 21-64	Treated by transsphenoidal surgery and RDT with or without DA treatment	NA (Surgery)
Samaan, 1986[21]	190	Retrospective cohort	Women with hyperprolactinemia and evidence of pituitary tumor	Range: 17-39	Bromocriptine vs Surgery; 88 patients underwent transsphenoidal excision, 102 were given 5 to 7.5 mg bromocriptine daily	36-60
Sartorio, 1990[22]	29	Prospective cohort	Hyperprolactinemic women with microprolactinoma	Range: 18-43	Treated with dopaminergic agents; 10 received 20-30 mg/d dihydroergocryptine, 8 7.5-10 mg/d bromocriptine and 11 were given 500 ug/ once a week cabergoline.	12

Shih, 1983[23]	30	Prospective cohort	Women with primary/secondary amenorrhea, galactorrhea, infertility who were found to be hyperprolactinemic	Range: 17-42	3 macros 1 micro treated with surgery; others with bromocriptine 2.5 for 3 days, 5 x 3d, then 7.5 daily	NR
Sluijmer, 1992[24]	59	Prospective cohort	Women with idiopathic hyperprolactinemia with menstrual irregularities and or infertility	26.5	Group 1 (10) patients who opted for no treatment, group 2 (33) treated at one time with bromocriptine (dose range 2.5 to 7.5mg BID), group 3 (16) Patients who were treated continuously with bromocriptine throughout the study (dose 2.5mg to 7.5mg BID)	Median: 78 Range: 6-190
Torres, 2006[25]	32	Retrospective cohort	Patients with macroprolactinomas	35+/-17.2	Patients were treated with dopamine agonist (type and dose not specified) alone, or with surgery alone	86.4
Touraine, 2001[26]	246	Retrospective cohort	Hyperprolactinemic women, not due to drug intake, hypothyroidism or chronic renal failure	29+/-0.6	191 patients were treated with bromocriptine. 32 underwent surgery and 23 received no treatment.	NR
van der Heijden, 1991[27]	41	Randomized controlled trial	Hyperprolactinemic patients- PRL exceeding 1500mU/l persistently	Range: 18-47	CV 205-502 0.025 mg q.H.S. x 3 days, then 0.05 mg x 3 days, then 0.075 mg q.H.S. always with AM placebo vs. bromocriptine 1.25 mg q.H.S. with q.A.M. placebo x 3 days, then bromocriptine 1.25 mg twice daily x 3 days, then 2.5 mg twice daily	6
Verhelst, 1991[28]	12	Randomized controlled trial	Hyperprolactinemic patients	Range: 19-56	Patients randomly assigned to receive bromocriptine starting dose 1.25mg increased weekly up to 5mg/day then to a max of 20mg/day or Quinagolide starting dose 0.025mg/day and increased weekly to 0.1mg/day maintenance and then increased monthly to obtain normalized PRL levels	6
Webster, 1994[29]	459	Randomized controlled trial	Hyperprolactinemic women with amenorrhea	31	Cabergoline 0.5 to 1.0 mg twice weekly vs. bromocriptine 2.5 to 5.0 mg twice daily	6

TSS - Transsphenoidal surgery; TCS – Transcranial surgery; DA – Dopamine agonist; RDT – Radiotherapy

Supplemental Table 2: Quality of the observational comparative studies

Author, Year	% of patients lost to followup	Length of follow-up (months)	Cohorts are similar at baseline?	Cohorts are representative of clinical practice?	Outcome assessment blinded?	Analysis adjusted for confounders?	Funding
Asano, 2001[1]	NR	42.48 (surgery)<br>22.44 (no-surgery)	No	Yes	No	NR	NR
Bahceci, 2010[2]	NR	NR	Yes	Yes	No	NR	NR
Brue, 1992[3]	0	96+/-48	Yes	No	No	NR	NR
Candrina, 1987[4]	NR	46+/-6	No	Yes	No	NR	NR
Colao, 1995[5]	NR	NR	Yes	Yes	No	NR	NR
De Rosa, 1998[6]	NR	6	Yes	Yes	No	NR	NR
Di Sarno, 2001[7]	NR	24	Yes	Yes	No	NR	NR
Di Somma, 1998[8]	NR	18	No	Yes	No	Yes	NR
Frey, 2009 [8]	NR	116.5	Yes	Yes	No	NR	NR
Hildebrandt, 1992[9]	0	12	No	Yes	No	No	Part-for-profit
Jeffcoate, 1996[12]	0	61.2+/-45.6	Yes	Yes	No	NR	Not-for-profit
Mahmood, 2010[14]	NR	2	Yes	Yes	No	NR	NR
Mattei, 1991[15]	0	44.9	Unclear	Yes	No	NR	NR
Perrin, 1991[18]	NR	NR	Yes	Yes	No	NR	NR
Pinzone, 2000[19]	NR	50.4	Unclear	Yes	No	NR	NR
Rush, 1991[20]	10	24-144	No	Yes	No	NR	NR
Samaan, 1986[21]	NR	42	Unclear	Yes	No	NR	Not-for-profit
Sartorio, 1990[22]	NR	12	Unclear	Yes	No	NR	For-profit
Shih, 1983[23]	0	10 (1-19)	Unclear	Yes	No	NR	NR
Sluijmer, 1992[24]	0	78 (6-190)	Unclear	Yes	No	NR	NR
Torres, 2006[25]	0	86.4+/-62.4	Unclear	Yes	No	NR	NR
Touraine, 2001[26]	NR	99.9+/-3.6	Unclear	Yes	No	NR	NR

Supplemental Table 3: Quality of the included observational dopamine withdrawal studies

Author, Year	% patients lost to followup	Length of follow-up (months)	Cohorts representative of clinical practice?	Outcome assessment blinded?	Analysis adjusted for confounders?	Funding
Biswas, 2005[30]	NR	84	Yes	No	Yes	NR
Cannavo, 1999[31]	NR	12	Yes	No	NR	NR
Ciccarelli, 1997[32]	4	1 to 82	Yes	No	NR	NR
Colao, 2003[33]	NR	24	Yes	No	Yes	Part-for-profit
Corenblum, 1988[34]	NR	NR	Yes	No	NR	NR
Di Sarno, 2000[35]	NR	12	Yes	No	NR	Not-for-profit
Eversmann, 1979[36]	NR	NR	Yes	No	NR	NR
Hancock, 1985[37]	0	19-88	Yes	No	NR	NR
Johnston, 1983[38]	NR	NR	Yes	No	NR	For-profit
Kharlip, 2009[39]	0	1-48	Yes	No	Yes	Not-for-profit
Liuzzi, 1985[40]	NR	21	Yes	No	NR	Not-for-profit
Mattei, 1984[41]	0	7 to 9	Yes	No	NR	Not-for-profit
Moriondo, 1985[42]	NR	24.8	Yes	No	NR	Not-for-profit
Muratori, 1997[43]	19	24	Yes	No	NR	Not-for-profit
Passos, 2002[44]	NR	NR	Yes	No	Yes	NR
Tartagni, 1995[45]	NR	NR	Yes	No	NR	NR
Touraine, 2001[26]	NR	99.9+/-3.6	Yes	No	NR	NR
van't Verlaat, 1991[46]	NR	12	Yes	No	NR	NR
Winkelmann, 1985[47]	NR	21.5	Yes	No	NR	NR
Wu, 2008[48]	NR	18	No	No	NR	NR
Zarate, 1983[49]	0	48	Yes	NR	No	NR

Supplemental Table 4: Quality of the randomized trials

Author, Year	% of patients lost to follow-up	Length of follow-up (months)	Blinding			Allocation Concealment	Funding
			P	CG	OA		
Hirahara, 1998[10]	4	12	NR	NR	NR	Yes	NR
Homburg, 1990[11]	10	6	Y	Y	NR	Yes	For-profit
Lappohn, 1992[13]	17	6	Y	NR	NR	Yes	NR
Motazedian, 2010[16]	NR	NR	NR	NR	NR	Yes	Not-for-profit
Pascal-Vigneron, 1995[17]	20	6	Y	Y	NR	NR	NR
van der Heijden, 1991[27]	NR	NR	Y	Y	NR	NR	NR
Verhelst, 1991[28]	NR	6	Y	Y	NR	No	NR
Webster, 1994[29]	2	6	Y	Y	NR	Yes	For-profit

P: Patients CG: Caregiver OA: Outcome assessor

Summary of uncontrolled studies of dopamine agonists (Supplemental Tables 5A-E)

Supplemental Table 5A: Bromocriptine studies

Study	Main findings
Al-Suleiman, 1989[50]	Symptomatic tumoral and non tumoral hyperprolactinemic women who presented with infertility. Most had resolution of galactorrhea, amenorrhea and infertility after treatment
Bergh, 1978[51]	Hyperprolactinemic women with secondary amenorrhea had reduction of PRL after treatment with restoration of ovulation
Brue, 1992[52]	After 6-24 months of follow up of patients with macroprolactinomas, tumor size and PRL levels were significantly reduced
Cannavo, 1992[53]	After 12 months of follow up of patients with microprolactinomas, tumor size was significantly reduced in most patients
Chattopadhyay, 2005[54]	After 31 months of follow up, men with macro and giant prolactinomas had significant reduction in PRL level, tumor size, restoration of libido and potency and improvement in visual field defects
Corenblum, 1983[55]	After 5-9 years of follow up of women with hyperprolactinemia, tumor size, PRL levels and hypogonadal symptoms were significantly reduced
Corenblum, 1988[34]	After 5 years of follow up of women with hyperprolactinemia, tumor size, PRL levels and hypogonadal symptoms were significantly reduced
Espinos, 1994[56]	After 6 months of follow up of patients with microprolactinomas, PRL levels were significantly reduced in most patients
Essais, 2002[57]	After 6 months of follow up of patients with macroprolactinomas, tumor size and PRL levels were significantly reduced in most patients
Falsetti, 1988[58]	Non tumoral hyperprolactinemia (per CT scan) treated with bromocriptine. PRL declined but many cases turned out to harbor microprolactinomas after extended follow up.
Fletes Rabago, 1991[59]	After 12 months of follow up of patients with hyperprolactinemia who received vaginal bromocriptine, PRL levels were significantly reduced
Greenspan, 1989[60]	Hyperprolactinemic patients who had reversal of hypogonadism by bromocriptine had improvement in bone mass
Haase, 1993[61]	After 36 months of follow up of patients with macroprolactinomas, tumor size and PRL levels were significantly reduced in most patients
Holtkamp, 1988[62]	Women with progressive metastatic breast cancer and hyperprolactinemia who are resistant to chemotherapy. Bromocriptine reduced PRL levels but did not improve sensitivity to chemotherapy
Jamrozik, 1996[63]	After 3.3 years of follow up of patients with macroprolactinomas, tumor size and PRL levels were significantly reduced in most patients
Lengyel, 1993[64]	After 12 months of follow up of patients with macroprolactinomas, tumor size and PRL levels were significantly reduced in most patients
Lin, 1992[65]	After 3-7 months of follow up of infertile women with hyperprolactinemia, 80% became pregnant
Moberg, 1991[66]	Post menopausal women with hyperprolactinemia. Most cases turned out to be due to macroadenomas. PRL level significantly decreased
Maraschini, 1991[67]	After 19 months of follow up of patients with micro and macro prolactinomas, PRL levels and tumor size were significantly reduced in most patients with clinical improvement in other symptoms
Merola, 1992[68]	After 9 months of follow up of patients with tumoral and non tumoral hyperprolactinemia, tumor size and PRL levels were significantly reduced in most patients

Molitch, 1985[69]	After 6-12 months of follow up of patients macroprolactinomas, tumor size and PRL levels were significantly reduced and improvement in visual field defects was noted
Mornex, 1978[70]	Most women with hyperprolactinemia amenorrhea and galactorrhea regained fertility
Moro, 1991[71]	After 6 months of follow up of patients with hyperprolactinemia, PRL levels were significantly reduced and most patients had clinical improvement
Paoletti, 1994[72]	After 6 months of follow up of patients with prolactinomas, tumor size decreased in most patients
Rasmussen, 1990[73]	After 5-13 years of follow up, a third of women with tumoral hyperprolactinemia had progression of tumor size although the overall course was benign for almost all patients
Schettini, 1990[74]	After 48 weeks of follow up of patients with tumoral and non tumoral hyperprolactinemia, tumor size and PRL levels were significantly reduced in most patients
Skrabanek, 1980[75]	Most symptomatic hyperprolactinemic patients had resolution of infertility and related symptoms
Spark, 1982[76]	After 27 months of follow up of patients with pituitary adenomas (majority had hyperprolactinemia, some had previous surgery or radiotherapy), tumor size, visual field defects and pituitary function improved in most patients
Thorner, 1978[77]	Most patients with hyperprolactinemia had improved PRL level and gonadal functions
Tsagarakis, 1995[78]	After 6 months of follow up of patients with prolactin and GH producing adenomas, PRL levels were significantly reduced in most patients
van 't Verlaat, 1986[79]	After 2.4 years of follow up of patients with macroprolactinomas, tumor size and PRL levels were significantly reduced. Endocrinopathies (hypogonadism, hypothyroidism and hypocorticism) also improved
Walsh, 1997[80]	Men (70% macro, 15% micro, 15% no mass) had significant reduction in PRL level, tumor size, and restoration of sexual function
Wass, 1982[81]	After 3-22 months of follow up of patients with large pituitary adenomas (majority had hyperprolactinemia), tumor size, visual field defects and pituitary function improved in most patients
Weingrill, 1992[82]	After 12 months of follow up of patients with hyperprolactinemia, PRL levels were significantly reduced and infertility resolved in most patients
Wu, 2006[48]	After 37.5 months of follow up of patients with giant invasive macroprolactinomas, tumor size and PRL levels were significantly reduced
Yang, 2011[83]	After 44 months of followup, 86% of patients with invasive prolactinoma receiving bromocriptine were clinically controlled, while 14% required surgical debulking
Zarate, 1983[49]	After 2 years of follow up of women with prolactinomas , PRL levels were significantly reduced

Supplemental Table 5B: Cabergoline studies

Study	Outcome
Bhansali, 2010[84]	After 6 months of followup, men undergoing rapid escalation of cabergoline over 3 weeks experienced improvement in symptoms (93%) and normalization of PRL levels in 93%, and reduction in tumor size by 50% (73%)
Biller, 1996[85]	After 48 weeks of followup, cabergoline was found to be highly effective for lowering prolactin, including in patients who had failed to achieve normal levels on bromocriptine
Bolko, 2003[86]	After 6 months of cabergoline treatment, the drug was found to have a high efficacy and a very good tolerability in the treatment of patients with pituitary adenomas
Byuykbayrak, 2010[87]	After 6 months of followup in hyperprolactinemic patients, varying doses of short-term maintenance therapy (8 weeks) after an initial 8 weeks of cabergoline 0.5 mg twice per week were found to be no different
Cho, 2009[88]	After 19 months of follow-up, men with invasive giant prolactinoma given cabergoline treatment for 3 months exhibited a mean decrease in serum PRL of 98% and mean reduction in tumor size of 85+/-4%, at 12 months reduction in tumor size was 97+/-1%
Ciccarelli, 1989[89]	After 6-12 months of followup, cabergoline was found to have a well-tolerated long lasting activity in treating hyperprolactinemic patients
Ciccarelli, 1997[32]	After 1-82 months of treatment with cabergoline, it was found that long term treatment with this medication is effective and well-tolerated in patients with tumorous or idiopathic hyperprolactinemia
Colao, 1997[90]	After 12-24 months of cabergoline therapy, cabergoline was found to be an effective first line pharmacological treatment for macroprolactinoma
Colao, 2000[91]	Treatment with cabergoline for 1-3 years was found to increase the prevalence of macroprolactinoma shrinkage at standard doses, more so in naïve patients, than in intolerant, resistant and responsive patients
Colao, 2004[92]	After 24 months of cabergoline treatment, an increase in prostate size was noticed, along with an increase in testosterone and DHT until normal levels were reached
Colao, 2004[93]	After 24 months of cabergoline treatment, prolactin levels normalization was achieved in the majority of men, along with the restoration of testosterone, GH and ACTH in approximately 60% of cases
Corsello, 2003[94]	After followup of 13-68 months, cabergoline was found to be highly effective in reducing prolactin levels and causing tumor shrinkage in patients with giant aggressive prolactinomas
De Bellis, 2008[95]	After 3 years of followup, the presence of anti pituitary antibodies (APA) in some patients with idiopathic hyperprolactinemia suggests a possible occurrence of autoimmune hypophysitis at potential/subclinical stage. Early and prolonged cabergoline therapy could interrupt the progression of to an overt clinical stage of the disease
De Rosa, 2006[96]	After 24 months of cabergoline treatment, gonadal function was restored in 66.7% of men with hyperprolactinemia
Delgrange, 1996[97]	After 5-134 weeks of treatment, cabergoline was associated with improved compliance, tolerability and efficacy. It was also found to be useful in patients who are intolerant or resistant to bromocriptine
Ferrari, 1992[98]	After 3-52 months of followup, cabergoline was found to be safe, efficacious, and very simple to use
Ferrari, 1997[99]	After 3 months to 8 years of followup, cabergoline was found to be an effective and well-tolerated treatment for macroprolactinoma patients
Muratori, 1997[43]	After 21 months of followup, cabergoline was found to be effective in inhibiting hormonal secretion and controlling tumor growth in pathological conditions like tumorous prolactin and GH hypersecretion
Ono, 2008[100]	After one year of treatment with cabergoline, it was found that individualized high-doses of it can normalize

	hyperprolactinemia and hypogonadism in nearly all prolactinomas irrespective of tumor size or preceding treatments
Ono, 2010[101]	After 8 years of follow-up after initiation of cabergoline in hyperprolactinemic infertile women with micro- or macroprolactinoma, hyperprolactinemia and ovulatory cycle was recovered in 100% of patients, 94% became pregnant
Pontikides, 2000[102]	12 months of cabergoline treatment, resulted in tumor shrinkage and normalization of PRL levels in all subjects
Raverot, 2009[103]	After 12 months of followup in patients with newly diagnosed macroprolactinoma treated with cabergoline, 18% (5/28) of patients had chiasmal herniation on MRI; of these, 60% (3/5) experienced visual field worsening
Shimon, 2007[104]	After followup of 1-50 months, cabergoline therapy was found to be effective and safe in men with giant prolactinomas
Stalldecker, 2010[105]	90 women with hyperprolactinemia achieved 103 pregnancies after cabergoline treatment for 1 to 120 months with doses ranging from 0.125 to 5 mg/week
Walia, 2011[106]	After 6 months of followup, men with macroprolactinoma who were given cabergoline for six months, exhibited a decrease in PRL levels and improvement in seminal volume, sperm count and motility.
Webster, 1993[107]	After approximately 49 weeks of followup, cabergoline was found to be highly effective in suppressing prolactin secretion and restoring gonadal function

Supplemental Table 5C: Quinagolide (CV 205-502) studies

Study	Outcome
Colao, 1996[108]	Following 6-24 months of CV 205-502 use, it was found that prolonged periods of use of this drug, were successful in normalizing hyperprolactinemia and in restoring gonadal function, with excellent tolerability
Duranteau, 1991[109]	Following 6 months of treatment with CV 205-502, the drug was found to be more potent than bromocriptine in reducing prolactin levels in some bromocriptine-resistant prolactinomas
Kvistborg, 1993[110]	After followup of up to 36 months, CV 205-502 was found to be efficacious and mostly well-tolerated, in treating patients with macroprolactinomas. It was also found to be easier to use than bromocriptine
Merola, 1994[111]	After 6-12 months of CV 205-502 treatment, prolactin levels normalized in most patients and induced tumor shrinkage in macroprolactinomas, all that with very few side effects, making it a useful alternative to ergot dopamine agonists
Morange, 1996[112]	After 6 months-3 years of followup, long term control of hyperprolactinemia by quinagolide was obtained in 39% of patients previously resistant to bromocriptine, and 75% of women resumed normal gonadal function
Newman, 1989[113]	Following 2 years of CV 205-502 treatment, the drug was found to be useful in the treatment of hyperprolactinemia
Nickelsen, 1993[114]	Following 6-12 months of treatment, quinagolide did not affect hormonal systems other than prolactin
Rasmussen, 1988[115]	After 6 months of CV 205-502 once a day treatment, the drug was found to be effective in decreasing serum prolactin levels and in restoring ovulatory function in hyperprolactinemic women, with few mild side effects
Rohmer, 2000[116]	After 1-48 months of followup, quinagolide had an anti-tumoral effect in 30% of study population (selected from ergot dopamine agonist resistant patients). Normalization of plasma prolactin was obtained in 44% of cases
Shoham, 1991[117]	After 3-24 months of followup, CV 205-502 in one daily dose, was found to be effective, safe, tolerable and a valuable alternative to the dopamine agonists
van der Heijden, 1989[118]	After 12-52 weeks of followup, it was concluded that CV 205-502 had good prolactin lowering effects in hyperprolactinemic women. It was also well-tolerated
van der Lely, 1991[119]	After 52 weeks of followup, CV 205-502 was found to effectively suppress prolactin secretion and shrink tumor size in the majority of prolactinoma patients
Van't Verlaat, 1990[79]	After a followup of 2-18 months (mean: 11), CV 205-502 in a once daily dose was found to be an effective and safe alternative in the long term treatment of macroprolactinomas
Vance, 1990[120]	After 24 weeks of CV 205-502 treatment, the drug was found to reverse hyperprolactinemia and to promote reduction in tumor size with reversal of visual abnormalities and restoration of gonadal function in most patients
Vilar, 1994[121]	After 1-24 months of quinagolide (CV 205-502) therapy, after reviewing past literature, it was concluded that, prolactin can be normalized in 16-20% of patients with bromocriptine resistance (dose dependent) and in 58% of patient with bromocriptine intolerance, using quinagolide CV 205-502. Half of all patients received this drug did experience side effects, and around 7% were quinagolide intolerant

Supplemental Table 5D: Bromocriptine short release vs long release

Study	Outcome
Merola, 1992[68]	After 9 months of followup, Parlodel SRO, a slow acting form of bromocriptine, was found to be very effective in the management of hyperprolactinemic syndromes
Moro, 1991[71]	After followup of 7 months, long acting bromocriptine forms, were shown to be as efficacious as short acting forms, in lowering plasma prolactin, while being more convenient and better tolerated
Weingrill, 1992[82]	After one year of followup, Parlodel SRO –a long acting oral bromocriptine- was found to be effective, tolerable and active for a long duration of time, making it an excellent alternative for the treatment of hyperprolactinemia

Supplemental Table 5E: Other dopamine agonist studies

Study	Outcome
Freda, 2000[122]	After 9-64 months of followup (mean: 27.4), pergolide was found to be effective, safe and generally well tolerated in long term treatment of macroprolactinomas
Jaspers, 1994[123]	After 6 months of followup, roxindol, a non-ergot dopamine agonist, was found to have a marked and prolonged prolactin-lowering activity that it may prove suitable for treatment of prolactinoma patients
Orrego, 2000[124]	Following 12 months of pergolide treatment, the drug was found to be safe, effective, and usually well tolerated, that it can be used as primary therapy for treatment of macroprolactinomas in men and women. Unlike bromocriptine, this dopamine agonists can be administered once daily
Sibal, 2002[125]	After a mean followup period of 4.2 years, it was determined that treating macroprolactinoma with dopamine agonists (bromocriptine, pergolide and cabergoline), had resulted in recovery of gonadotroph, thyrotroph and corticotroph function in 62, 44 and 67% of cases
Verde, 1980[126]	Following 2-25 months of Lisuride treatment, it was found that this dopamine agonist can control prolactin levels and in responsive individuals influence tumor size

Supplemental Figure 1A: Bromocriptine vs. Quinagolide
Prolactin Level (ng/mL)

Study name	Difference in means	Lower limit	Upper limit	Bromo	CV
Lappohn	-35.07	-88.45	18.31	12	12
Colao	163.30	-168.08	494.68	10	16
**PRL level**	**-6.07**	**-143.43**	**131.30**	**22**	**28**

(Statistics for each study)

Difference in means and 95% CI

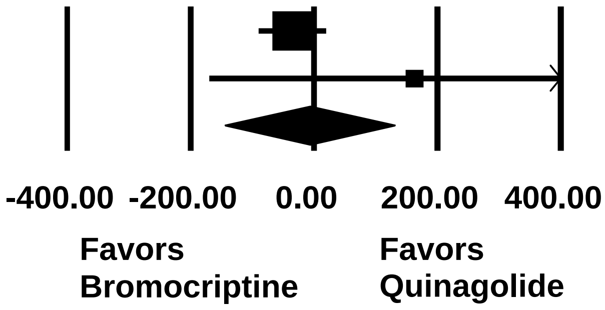

Supplemental Figure 1B: Bromocriptine vs. Quinagolide
Clinical Outcomes

Outcome	Study name	Risk ratio	Lower limit	Upper limit	Bromo	CV
	van der Heijden	0.69	0.20	2.41	3 / 12	4 / 11
	Lappohn	1.50	0.30	7.43	3 / 12	2 / 12
	Colao	0.56	0.11	2.79	2 / 13	3 / 11
	Verhelst	1.50	0.13	17.67	1 / 4	1 / 6
	Homburg	2.00	0.21	18.98	2 / 11	1 / 11
		**0.95**	**0.45**	**2.00**	**11 / 52**	**11 / 51**
	van der Heijden	1.91	0.04	83.79	1 / 6	1 / 11
	Colao	0.70	0.02	32.45	1 / 14	1 / 10
	Homburg	4.00	0.21	74.84	2 / 8	1 / 8
**Galactorrhea, I^2=0%**		**2.05**	**0.28**	**14.88**	**3 / 27**	**2 / 28**
	Colao	0.91	0.11	7.84	2 / 11	1 / 5
**Decreased libido**		**0.91**	**0.11**	**7.84**	**2 / 11**	**1 / 5**
	Colao	1.00	0.08	11.93	1 / 5	1 / 5
**Erectile dysfunction**		**1.00**	**0.08**	**11.93**	**1 / 5**	**1 / 5**
	van der Heijden	1.17	0.65	2.10	5 / 6	5 / 7
	Homburg	2.25	0.41	12.28	3 / 4	1 / 3
**Female infertility**		**1.25**	**0.72**	**2.19**	**8 / 10**	**6 / 10**
	van der Heijden	2.24	0.66	7.62	7 / 24	3 / 23
	Verhelst	1.05	0.40	2.75	3 / 5	4 / 7
	Di Somma	1.15	0.03	50.38	1 / 7	1 / 8
	Homburg	6.00	0.86	41.96	6 / 11	1 / 11
**PRL level increased. I^2=0%**		**1.67**	**0.84**	**3.35**	**17 / 47**	**9 / 49**
	Di Somma	1.15	0.03	50.38	1 / 7	1 / 8
**Low testosterone level**		**1.15**	**0.03**	**50.38**	**1 / 7**	**1 / 8**
**Amenorrhea/ oligomenorrhea, I^2=0%**						

Risk ratio and 95% CI

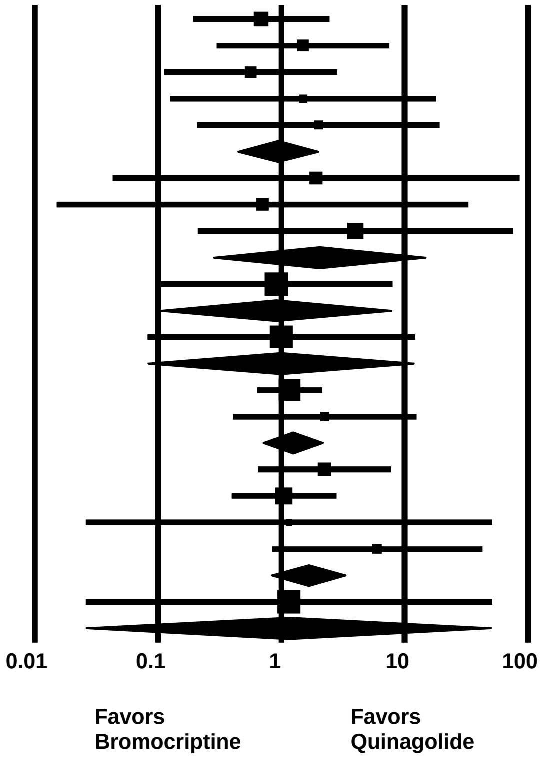

Supplemental Figure 2A: Dopamine Agonists vs. No Treatment
Prolactin Level (ng/mL)

Study name	Statistics for each study			Sample size	
	Difference in means	Lower limit	Upper limit	DA	No treatment
Touraine	-53.90	-95.96	-11.84	191	23
Jeffcoate	-28.90	-81.10	23.30	23	10
**PRL level**	**-44.06**	**-76.81**	**-11.31**	**214**	**33**

Difference in means and 95% CI

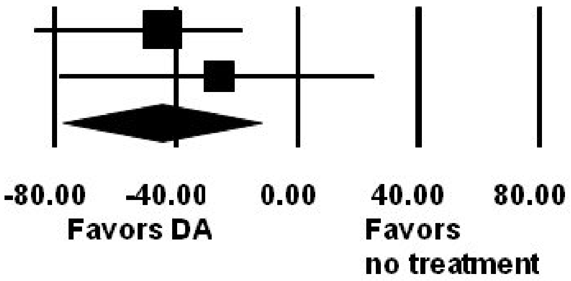

Supplemental Figure 2B: Dopamine Agonists vs. No Treatment
Clinical Outcomes

Outcome	Study name	Risk ratio	Lower limit	Upper limit	Events / Total DA	Events / Total No treatment
	Sluimer	0.38	0.04	3.52	1 / 12	2 / 9
	Mattei	0.95	0.89	1.01	91 / 97	36 / 36
**Amenorrhea/oligomenorrhea**		**0.95**	**0.89**	**1.01**	**92 / 109**	**38 / 45**
	Sluimer	0.45	0.01	17.03	1 / 6	1 / 3
**Galactorrhea**		**0.45**	**0.01**	**17.03**	**1 / 6**	**1 / 3**
	Sluimer	1.20	0.20	7.05	4 / 10	1 / 3
	Hirahara	2.75	0.31	24.52	3 / 24	1 / 22
**Infertility**		**1.67**	**0.42**	**6.60**	**7 / 34**	**2 / 25**
	Hirahara	0.30	0.10	0.94	3 / 21	10 / 21
**Miscarriages**		**0.30**	**0.10**	**0.94**	**3 / 21**	**10 / 21**
	Sluimer	0.63	0.16	2.51	3 / 16	3 / 10
	Mattei-Idiopathic	0.84	0.72	0.97	38 / 46	21 / 21
	Mattei-Prolactinoma	0.96	0.83	1.11	55 / 61	16 / 17
**PRL Level**		**0.90**	**0.81**	**0.99**	**96 / 123**	**40 / 48**
	Mattei	0.83	0.04	16.66	2 / 107	0 / 17
**Tumor increase**		**0.83**	**0.04**	**16.66**	**2 / 107**	**0 / 17**

Risk ratio and 95% CI

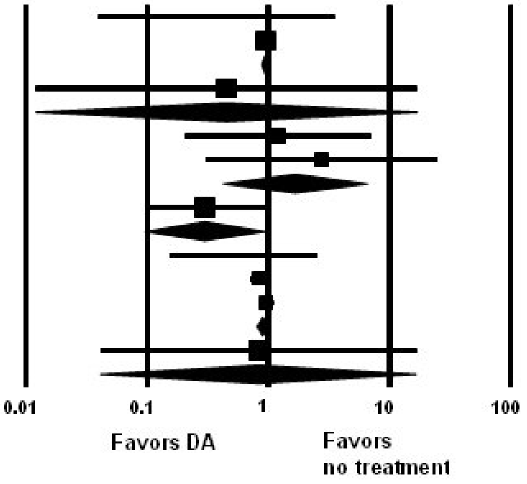

Supplemental Figure 3: Dopamine Agonists vs. Surgery
Clinical Outcomes

Outcome	Study name	Risk ratio	Lower limit	Upper limit	Events / Total DA	Events / Total Surgery
	Shih-Microprolactinoma	0.16	0.00	5.34	1 / 10	1 / 2
	Shih-Macroprolactinoma	2.00	0.45	8.94	1 / 1	1 / 3
**Amenorrhea/oligomenorrhea**		0.95	0.10	9.15	2 / 11	2 / 5
	Torres	3.33	0.29	38.75	2 / 2	0 / 1
**Cranial nerve dysfunction**		**3.33**	**0.29**	**38.75**	**2 / 2**	**0 / 1**
	Torres	1.50	0.08	29.15	1 / 7	0 / 3
**Galactorrhea**		**1.50**	**0.08**	**29.15**	**1 / 7**	**0 / 3**
	Torres	2.42	0.71	8.26	9 / 13	2 / 7
**Headaches**		**2.42**	**0.71**	**8.26**	**9 / 13**	**2 / 7**
	Samaan-Microprolactinoma	4.69	1.53	14.41	22 / 50	3 / 32
	Samaan-Macroprolactinoma	1.00	0.28	3.61	2 / 6	7 / 21
**Female infertility**		**2.24**	**0.49**	**10.17**	**24 / 56**	**10 / 53**
	Torres	0.47	0.30	0.75	11 / 25	7 / 7
	Shih-Microprolactinoma	0.16	0.00	5.34	1 / 10	1 / 2
	Shih-Macroprolactinoma	2.33	0.08	71.44	1 / 2	1 / 4
	Brue	0.67	0.15	2.98	2 / 6	2 / 4
**PRL level increased, I2=0%**		**0.49**	**0.32**	**0.76**	**14 / 42**	**10 / 16**
	Shih-Microprolactinoma	0.16	0.00	5.34	1 / 10	1 / 2
	Shih-Macroprolactinoma	2.33	0.08	71.44	1 / 2	1 / 4
	Brue	0.69	0.02	28.89	1 / 7	1 / 5
**Increase in tumor size**		**0.65**	**0.08**	**5.04**	**2 / 18**	**2 / 10**
	Torres	0.57	0.28	1.15	5 / 10	3 / 3
**Visual field defects**		**0.57**	**0.28**	**1.15**	**5 / 10**	**3 / 3**

Risk ratio and 95% CI

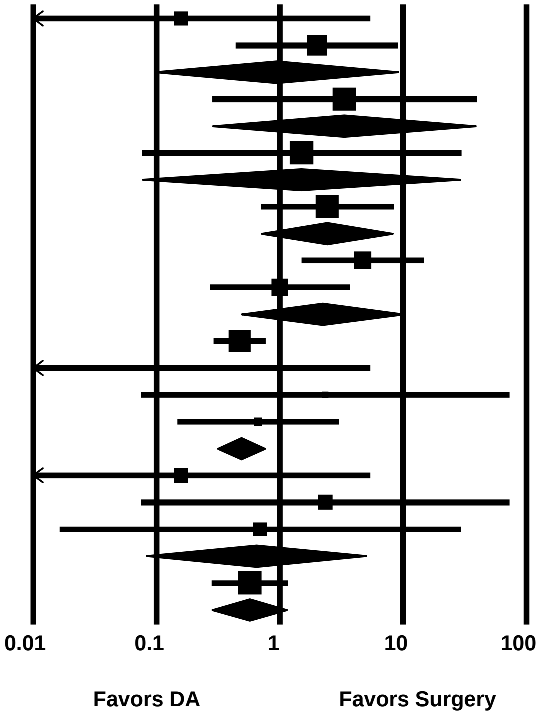

Supplemental Figure 4A: Dopamine Agonists vs. Dopamine Agonists + Surgery
Prolactin level (ng/mL)

Study name	Statistics for each study: Difference in means	Lower limit	Upper limit	Sample size: DA	DA + Surgery
Asano	1197.30	-677.86	3072.46	5	8

Difference in means and 95% CI

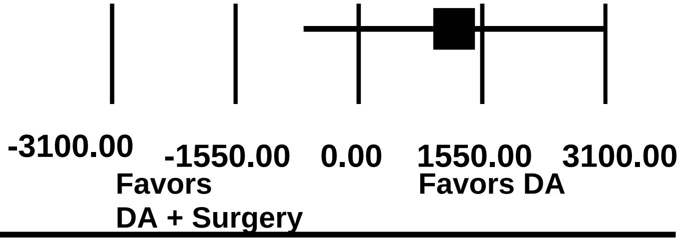

Supplemental Figure 4B: Dopamine Agonists vs. Dopamine Agonists + Surgery
Clinical Outcomes

Outcome	Study name	Risk ratio	Lower limit	Upper limit	DA	DA + Surgery
PRL levels increased	Hildebrandt	0.71	0.23	2.20	4 / 14	4 / 10
Tumor size increased	Hildebrandt	0.72	0.02	33.64	1 / 15	1 / 11

Risk ratio and 95% CI

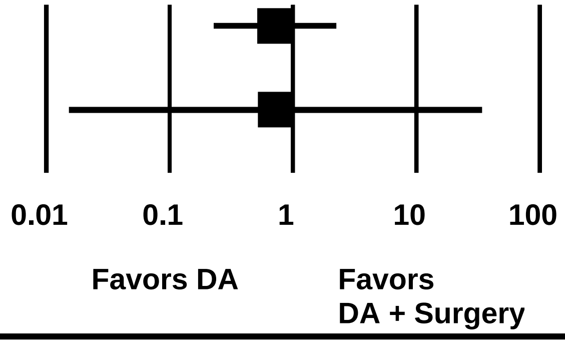

Supplemental Figure 5A: Surgery vs. Surgery + Dopamine Agonists
Prolactin level (ng/mL)

Study name	Statistics for each study			Sample size	
	Difference in means	Lower limit	Upper limit	Surgery	Surgery + DA
Rush	-2.37	-1569.01	1564.27	8	12
Candrina	-1580.00	-4201.57	1041.57	15	6
**PRL level**	**-426.67**	**-1797.74**	**944.41**	**23**	**18**

Difference in means and 95% CI

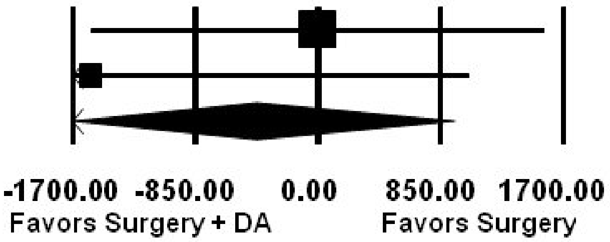

Supplemental Figure 5B: Surgery vs. Surgery + Dopamine Agonist
Clinical Outcomes

Outcome	Study name	Risk ratio	Lower limit	Upper limit	Surgery	Surgery + DA
	Perrin	3.50	0.83	14.83	7 / 20	2 / 20
**Endocrine dysfunction**		**3.50**	**0.83**	**14.83**	**7 / 20**	**2 / 20**
	Candrina	5.60	0.39	79.70	3 / 4	0 / 3
**Amenorrhea/oligomenorrhea**		**5.60**	**0.39**	**79.70**	**3 / 4**	**0 / 3**
	Perrin-Microprolactinoma	4.00	0.56	28.40	4 / 8	1 / 8
	Perrin-Macroprolactinoma	1.25	0.78	2.01	10 / 12	8 / 12
**PRL level increased**		**1.49**	**0.66**	**3.38**	**14 / 20**	**9 / 20**
	Candrina	9.19	0.62	135.95	10 / 15	0 / 6
**Tumor size increased**		**9.19**	**0.62**	**135.95**	**10 / 15**	**0 / 6**
	Candrina	1.00	0.30	3.32	4 / 8	2 / 4
**Visual Field defects**		**1.00**	**0.30**	**3.32**	**4 / 8**	**2 / 4**

Risk ratio and 95% CI

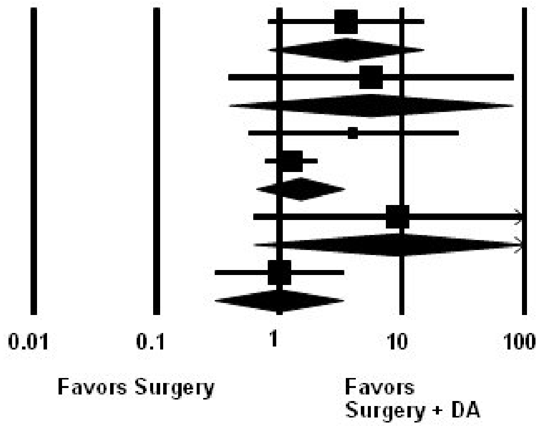

Subgroup analyses (Supplemental Tables 6A-D)

Supplemental Table 6A: Bromocriptine vs. Cabergoline

**Outcome/subgroup**	**# of Studies**	**RR (95% CI)**	I^2	$P_{interaction}$
**PRL level increased**				
Macro	2	2.89 (1.54 to 5.41)	NA	0.71
Micro	2	3.49 (1.62 to 7.51)	NA	
Men	2	1.69 (0.6 to 7.90)	NA	0.37
Women	2	4.05 (1.29 to 12.68)	NA	
**Amenorrhea/oligomenorrhea**				
Macro	1	4.83 (0.95 to 24.55)	NA	0.22
Micro	2	0.68 (0.04 to 10.05)	NA	
**Galactorrhea**				
Men	1	0.69 (0.02 to 28.89)	NA	0.73
Women	1	1.80 (0.04 to 89.09)	NA	
**Decreased libido**				
Macro	1	0.28 (0.04 to 2.24)	NA	0.91
Micro	1	0.37 (0.01 to 15.25)	NA	
**Increased tumor size**				
Macro	1	0.512 (0.01 to 24.04)	NA	0.97
Micro	1	0.47 (0.01 to 19.04)	NA	

Supplemental Table 6B: Bromocriptine vs. CV 205-502

**Outcome/subgroup**	**# of Studies**	**RR (95% CI)**	I^2	$P_{interaction}$
**PRL Levels**				
Men	1	1.15 (0.03 to 50.38)	NA	0.64
Women	2	2.96 (1.05 to 8.35)	NA	
**Amenorrhea/oligomenorrhea**				
Macro	1	0.56 (0.11 to 2.79)	NA	0.72
Micro	2	0.81 (0.26 to 2.47)	NA	
**Galactorrhea**				
Macro	1	0.70 (0.02 to 32.45)	NA	0.72
Micro	1	1.91 (0.04 to 83.79)	NA	

Supplemental Table 6C: DA vs. Surgery

Outcome/subgroup	# of Studies	RR (95% CI)	I^2	$P_{interaction}$
**PRL level increased**				
Macro	2	0.49 (0.31 to 0.77)	NA	0.14
Micro	1	0.16 (0.00 to 5.34)	NA	
**Amenorrhea/oligomenorrhea**				
Macro	1	2.00 (.045 to 8.94)	NA	0.36
Micro	1	0.16 (0.00 to 5.34)	NA	
**Increased tumor size**				
Macro	1	2.33 (0.08 to 71.44)	NA	0.28
Micro	1	0.16 (0.001 to 5.34)	NA	

Supplemental Table 6D: Withdrawal from DA

Outcome/subgroup	# of Studies	RR (95% CI)	I^2	$P_{interaction}$
**PRL level increased**				
Macro	4	0.60 (0.41 to 0.76)	40.1	0.74
Micro	5	0.64 (0.47to 0.78)	63.7	
**Amenorrhea/oligomenorrhea**				
Macro	4	0.41 (0.1 to 0.82)	82.3	0.75
Micro	6	0.33 (0.1 to 0.67)	86.0	
**Increased tumor size**				
Macro	4	0.1 (0.04 to 0.24)	59.4	0.49
Micro	3	0.03 (0.01 to 0.09)	0%	
**Galactorrhea**				
Macro	4	0.20 (0.05 to 0.56)	75.4	0.90
Micro	5	0.23 (0.06 to 057)	78.2	
**Decreased sexual potency and libido**				
Macro	3	0.19 (0.09 to 0.36)	83.4	0.63
Micro	1	0.23 (0.08 to 0.52)	NA	

Summary of uncontrolled studies of radiotherapy, surgery, combinations of treatment, and pregnancy (Supplemental Tables 7A-F)

Supplemental Table 7A: Radiotherapy studies

Study	Main findings
Jezkova, 2009[127]	80% of patients with prolactinoma who failed DA treatment achieved normoprolactinemia in 96 months (mean) with gamma knife irradiation with halt in growth in 97% of patients.
Pan, 2000[128]	Nearly all patients with prolactinomas treated with gamma knife radiosurgery as a primary treatment achieved tumor control while clinical cure was achieved in over 50% of cases with associated clinical improvement followed for 33.2 months mean with higher doses associated with better clinical outcomes.
Pouratian, 2006[129]	26% of patients with medically and surgically refractory prolactinomas treated with gamma knife radiosurgery achieved clinical remission with low complication rates.
Sun, 2010[130]	In patients with prolactin-secreting adenomas, normalization of PRL occurred in 5/7 (71%) after fractionated radiotherapy, and 0/1 (0%) of patients after stereotactic radiosurgery.
Tsagarkis, 1991[131]	In women with prolactinomas, 50% had normalization of PRL, 28% had reduction in PRL after treatment megavoltage radiotherapy with 8 years follow-up
Yoon, 1998[132]	46% of patients with prolactinomas had normalization of hormonal levels after 1 year of stereotactic radiosurgery compared with 2 years in conventional radiation therapy with similar complication rates.
Zhang, 2000[133]	In patients with hypersecreting pituitary adenomas followed for average 31.6 months, gamma knife radiosurgery as a primary treatment may be safe and effective.
Zierhut,1995[134]	In patients with prolactinomas that were inoperable or had unsuccessful drug treatment who were treated with radiotherapy, all patients achieved persistent reduction of the prolactin level and 27% had normalization of PRL levels with mean follow-up of 6.5 years.

Supplemental Table 7B: Combination of radiotherapy and dopamine agonists studies

Study	Main findings
Kelly, 1978 [135]	Women with hyperprolactinemia and infertility were treated with pituitary implanted radiotherapy with mean follow-up of 27 months, with nearly 75% achieving fertility alone or in combination with bromocriptine.
Littley, 1991[136]	External radiotherapy is effective in reducing serum prolactin levels in patients with pituitary macroadenomas, especially in prolactin-producing tumors with observation of up to 154 months.
Rudoler, 1996[137]	Patients with macroprolactinomas received external beam radiotherapy for residual or recurrent disease after surgery alone or in conjunction with bromocriptine with follow-up for 140 months showed 10 and 15 year freedom from recurrence rates of 95% and 71% respectively with improvement in visual symptoms and prolactin levels in >80% of patients with complications of new or worsening endocrinopathies in 50% of patients.
Tanaka, 2010[138]	After 12 months of followup, 100% of patients with prolactinoma refractory to dopamine agonists who underwent stereotactic radiosurgery achieved tumor control, 77% had a reduction in size > 2mm. Dopamine agonist therapy was continued after radiosurgery until PRL normalized then was discontinued. 18% of patients had biochemical remission at 34 months after radiosurgery.

Supplemental Table 7C: Surgery studies

Study	Outcome
Amar, 2002[139]	After 6-18 years of followup, it was concluded that prolactin levels lower than 10ng/ml on post-op-day 1 predict a long term chemical cure in patients with pituitary adenoma, after undergoing transsphenoidal surgery
Babey, 2011[140]	After 33.5 months of followup, 90% of patients experiences improvement in signs and symptoms, 94% of patients had postoperative PRL levels that returned to normal in patients with small prolactinomas who underwent transsphenoidal surgery
Barbarino, 1982[141]	After 8-24 months of followup, it was found that total selective removal of the microadenoma acutely decreases the prolactin concentration, but a functional inhibition of the normal lactotrope can persist for a period of few months following surgery in some patients
Charpentier, 1985[142]	After average followup of 4.4 years, selective surgical adenomectomy was found to be the only treatment able to achieve a definitive cure with a low iatrogenic risk, in patients with prolactinomas
Dusick, 2008[143]	After at least 3 months of followup, it was found that, following transsphenoidal surgery on pituitary adenoma hyperprolactinemia resolution occurred in 67% of patients
Fatemi, 2008[144]	After 8 years of followup, it was concluded that after transsphenoidal adenomectomy, new unplanned hypopituitarism occurred in approximately 5% of patients, whereas improved hormonal function occurred in 50% of patients, and that new hypopituitarism occurs most commonly in patients with tumors larger than 20 mm in size
Feigenbaum, 1996[145]	After 9 years of followup, it was concluded that, women undergoing transsphenoidal surgery for prolactin-secreting adenoma form a heterogenous patient population and that the best long term results would be achieved in the pure prolactinoma group, for which this surgery is safe and effective
Gokalp, 2000[146]	After 1-10 years of followup, it was concluded that transsphenoidal microsurgery is a safe and efficient approach and can be recommended as an alternative to medical treatment, or for patients who are intolerant or resistant to dopamine agonists
Guidatti, 1987[147]	After 11 years of followup, it was determined that, transcranial surgery was preferred over transsphenoidal in debulking large adenomas. It was also determined, that post-operative radiotherapy was of value in invasive adenomas and in cases in which tumor removal was not radical
Hamilton, 2005[148]	After surgically intervening on 79 prolactinoma patients that are either resistant or intolerant to medical therapy, it was concluded that transsphenoidal surgery often provides a suitable option for treating that category of patients.
Hirohata, 1991[149]	After a mean followup period of 60 months, pregnancy was found to have no effect on prolactin level, in patients with normal post-operative hormone levels
Laws, 1985[150]	After 37 months of followup, 31% of patients receiving secondary transsphenoidal surgery for prolactinoma were deemed successful but with higher complication rates postoperatively
Maira, 1989[151]	After an average followup of 3.4 years, it was found that a slightly elevated postsurgical prolactin level does not imply that tumoral tissue is still present
Massoud, 1996[152]	After 10-20 years of followup, it was concluded that most of the patients who received transsphenoidal surgery for their microprolactinoma were asymptomatic. Furthermore, those who have had late relapse of hyperprolactinemia, it was found their hyperprolactinemia was mild, non symptomatic and functional
Nakagawa, 2001[153]	Following transsphenoidal surgery on 13 patients with prolactinomas, the result was normalization of prolactin levels post-op in 9 patients, with preservation of normal pituitary function
Pandey, 2005[154]	After a followup period ranging from 3 months, up to 10 years, it was concluded that the transsphenoidal approach is an effective and safe surgical modality for pituitary macroadenomas

Qu, 2011[155]	After 45 months of followup, men with prolactinoma undergoing transsphenoidal surgery, 52.9% of patients achieved initial remission (83.3% of microadenomas; 44.9% of macroadenomas). The long-term remission rate was 42.5% while relapse of hyperprolactinemia occurred in 19.6% of cured patients. The 5-year recurrence-free survival was 78.2%
Rawe, 1980[156]	After 18 months of followup, it was concluded that transsphenoidal microsurgical removal of prolactinomas is a highly effective approach, leading to significant normalization of preoperative prolactin levels, resumption of menses and the recovery of fertility in patients with microadenomas
Saitoh, 1986[157]	Following the surgical intervention on 98 patients with prolactinomas, it was found the post-operative course was closely related to tumor size and the preoperative levels of prolactin. Prolactin levels returned to normal in 37, 33% in patients with microadenomas, and expansive macroadenomas in that order. Furthermore, 35% of premenopausal women resumed normal menopause
Santoro, 2007[158]	After 56 months of followup, it was concluded that transsphenoidal surgery is an effective treatment for secreting pituitary tumors. Long term endocrinologic followup was recommended due to possibility of tumor recurrence
Scamoni, 1991[159]	After a followup period ranging from 18-120 months, transsphenoidal microsurgery was deemed the therapy of first choice for microadenoma and mesoadenoma treatment, especially with the presence of bromocriptine intolerance
Shen, 2000[160]	Following endoscopic endonasal transsphenoidal surgery on 18 patients with prolactinomas and subsequent 2-26 month followup, complete resolution was achieved in 78% of patients, 1 patient with recurrent microprolactinoma required second operation, all without complications related to the surgical approach
Sinha, 2011[105]	After 39 months of followup in patients with prolactinoma undergoing surgery either transsphenoidally or transcranially, hormonal remission was achieved in 44% (83% microadenomas, 48% macroadenomas, and 16% of giant adenomas). The overall mortality was 1.7%, all who had giant invasive tumors.
Soule, 1996[161]	After 38 months of followup of 34 patients, who underwent pituitary surgery for micro and macroprolactinomas, the cure rate in a tertiary referral centre, was relatively low compared to the results achieved in the past when surgery was used as a primary therapeutic modality
Webster, 1992[162]	After a mean followup period of 51 months, it was concluded that in carefully selected patients, partial hypophysectomy is an acceptable alternative to medical treatment for prolactinoma
Wolfsberger, 2003[163]	After 2-13 years of following up 11 men who received transsphenoidal surgery for pituitary microprolactinoma, it was concluded that microadenomas in men are potentially curable by transsphenoidal adenomectomy alone with low complication and recurrence rates in the hands of an experienced surgeon
Zhang, 2008[164]	After 13 months of followup, transsphenoidal surgery was found to be a safe and effective treatment for pituitary microadenoma, and that in order to obtain better effects; it must be ordered as first-line treatment for patients with locally non-invasive prolactin secreting microadenoma

Supplemental Table 7D: Combination of surgery and dopamine agonists studies

Study	Main findings
Jamjoom, 1995[165]	In large prolactin-secreting adenomas is high, only 27% of patients were normoprolactinemic after surgery; the rest required additional postoperative treatment with surgery and dopamine agonist therapy achieving overall normalization rates of 71% over a 10 year period.
Saeki, 1998[166]	Patients with giant prolactinomas with poor initial response to bromocriptine alone were treated with continued bromocriptine achieving normalization in 60% of cases while the remaining 40% required surgery
Thompson, 2002[167]	Patients with microprolactinoma who underwent transsphenoidal surgery were followed for 15-21 years with initial normalization in all patients. 18.2% experienced recurrent hyperprolactinemia at a mean interval of 5.3 years but was permanent in only 5% of patients.
Thomson, 1994[168]	Women with microprolactinoma who underwent transsphenoidal surgery were followed for 10 years had recurrence in 12% of patients.
Touraine, 2001[26]	Hyperprolactinemic women with menstrual disorders were followed for 100 months treated with bromocriptine with disappearance of adenoma in 45% and nearly all stabilization of tumor size in 40%; surgery which led to disappearance of adenoma in almost all cases but failed to provide a definitive cure.

Supplemental Table 7E: Combination of surgery and radiation therapy studies

Study	Main findings
Oruckaptan, 2000[169]	Patients with pituitary adenomas treated surgically with or without postoperative radiotherapy with mean follow-up time 40.5 months. Surgery alone achieved tumor control in 79.6% of patients and in 78.12% of those who received radiotherapy after surgery. Topical application of bromocriptine into the sellar cavity after tumor removal seems to provide superior results.
Rush, 1997[170]	After a median followup of 8 years, 97% of patients with pituitary macroadenomas receiving combination surgery and postoperative radiotherapy had regression or stabilization of the adenoma, while 84% with visual impairment improved

Supplemental Table 7F: Pregnancy studies

Study	Outcome
Badawy, 1997[171]	After a followup period of 1-14 years, it was concluded that it is safe for patients with prolactinomas to achieve pregnancy following bromocriptine treatment. It was also suggested that pregnancy might lead to a slight decrease in the size of prolactinomas, increase in size, no change, and in some cases complete resolution.
Berinder, 2007[172]	After examining the records of 271 women with primary hyperprolactinemia, it was concluded that parity was reduced compared to normal controls. However, there were no increased risks of adverse pregnancy or delivery outcomes once a woman with treated hyperprolactinemia became pregnant.
Ciccarelli, 1997[32]	After a followup period of 1-82 months (mean: 28.3) it was found that prolactin level in pregnant hyperprolactinemic women were within the normal limits, apart from one case (out of nine) which had a higher prolactin level towards the third trimester. All pregnancies had normal courses and outcomes.
Colao, 2008[173]	After a followup period of 12 years, it was concluded that fetal exposure to cabergoline through early pregnancy does not induce any increase in the risk of miscarriage of fetal malformation.
Cristiani, 1985[174]	After following up on 17 bromocriptine-induced pregnancies in hyperprolactinemic patients, it was observed that plasma prolactin levels were elevated during the entire pregnancy without the progressive rise, as in normal pregnancies. Pregnancies were all uneventful, with only one patient complaining of headache.
Crosignani, 1989[175]	After followup of about 6 months, it was concluded that pregnancy itself normalizes plasma prolactin in 17% of hyperprolactinemic women.
Crosignani, 1992[176]	After 6-18 months of followup of hyperprolactinemic women, it was suggested that prolactin levels can be normalized during pregnancy, and that pregnancy is quite safe for these patients.
Godo, 1989[177]	After following up on 50 hyperprolactinemic women, it was found that pregnancy did not increase the risk of pregnancy, delivery and nursing complications.
Holmgren, 1986[178]	After following up on 35 prolactinoma women, through 41 pregnancies, it was concluded that women with hyperprolactinemia, irrespective of sella pathology, should be treated with bromocriptine for at least one year before pregnancy is recommended.
Jeffcoate, 1996[179]	After followup period of 2.8+/-2.7 years, it was concluded that hyperprolactinemia will be self-limiting in up to one-third of women, and that pregnancy may be one factor which triggers a return to normal function.
Karunakaran, 2001[180]	After followup of 1-19 years, it was concluded that women with hyperprolactinemia who pass through menopause or through pregnancy have a significant chance of normalizing their prolactin levels.
Kelly, 1979[181]	After following up on 27 women through 41 pregnancies, it was found that tumor enlargement as shown by diminished visual acuity, visual field defects, severe headaches, diabetes insipidus and radiological changes occurred only in 3 of 14 patients who had not had pituitary implantation with 90 yttrium.
Kupersmith, 1994[182]	After following up on 65 hyperprolactinemic women, during 111 pregnancies, it was concluded that the risk of developing visual loss during single or multiple pregnancies in patient with microadenoma was small. Six of eight pregnant women with macroadenomas, however, developed visual field loss during pregnancy,
Mattei, 1991[15]	After a followup of 6-180 months (mean: 44.9), it was concluded pregnancy had a beneficial, yet unexplained effect on prolactin in women with pathologic hyperprolactinemia.
Ono, 2010[101]	After 8 years of follow-up after initiation of cabergoline in hyperprolactinemic infertile women with micro- or macroprolactinoma, hyperprolactinemia and ovulatory cycle was recovered in 100% of patients, 94% became pregnant with

	95 pregnancies (93 achieved while still on cabergoline). Of the 93, 86 resulted in 93 single live births, one stillbirth, and two abortions with 7 ongoing at the conclusion of the study. All babies were born healthy without malformations.
Rasmussen, 1985[183]	After followup of 58 hyperprolactinemic women for 13-108 months, it was concluded that bromocriptine induction of ovulation and pregnancy followed by lactation in these women, did not worsen their condition but rather a considerable number of them had a decrease in prolactin hypersecretion and even experienced the return of spontaneous uterine bleedings.
Rossi, 1995[184]	After examining the outcome of 103 pregnancies in 64 women with constant hyperprolactinemia, it was found 66% of all pregnancies ended in delivery, 17% in miscarriages, 10% in tubal pregnancy and 7% in induced abortion. The only significant effect of hyperprolactinemia was determined to be an increased risk of tubal pregnancy
Stalldecker, 2010[105]	90 women with hyperprolactinemia achieved 103 pregnancies after cabergoline treatment for 1 to 120 months with doses ranging from 0.125 to 5 mg/week with no significant complications during pregnancy. 7.2% had spontaneous abortions, preterm deliveries occurred in 8.8%, and neonatal abnormalities were seen in 3.6%.
Woodhouse, 1985[185]	After following up 14 previously infertile patients with hyperprolactinemia, it was concluded that fertility can be restored using bromocriptine even in patients with a large prolactin secreting tumor.
Zarate, 1979[186]	After 6-14 months of post pregnancy followup, it was concluded that patients with microadenomas can be allowed to become pregnant on bromocriptine alone, provided they are carefully supervised during pregnancy.

References:

1. Asano S, Ueki K, Suzuki I, Kirino T: **Clinical features and medical treatment of male prolactinomas.** *Acta Neurochirurgica* 2001, **143:**465-470.
2. Bahceci M, Sismanoglu A, Ulug U: **Comparison of cabergoline and bromocriptine in patients with asymptomatic incidental hyperprolactinemia undergoing ICSI-ET.** *Gynecol Endocrinol* 2010, **26:**505-508.
3. Brue T, Pellegrini I, Priou A, Morange I, Jaquet P: **Prolactinomas and resistance to dopamine agonists.** *Horm Res* 1992, **38:**84-89.
4. Candrina R, Galli G, Bollati A, Pizzocolo G, Orlandini A, Gualandi GF, Giustina G: **Results of combined surgical and medical therapy in patients with prolactin-secreting pituitary macroadenomas.** *Neurosurgery* 1987, **21:**894-897.
5. Colao A, Merola B, Sarnacchiaro F, Di Sarno A, Landi ML, Marzullo P, Cerbone G, Ferone D, Lombardi G: **Comparison among different dopamine-agonists of new formulation in the clinical management of macroprolactinomas.** *Horm Res* 1995, **44:**222-228.
6. De Rosa M, Colao A, Di Sarno A, Ferone D, Landi ML, Zarrilli S, Paesano L, Merola B, Lombardi G: **Cabergoline treatment rapidly improves gonadal function in hyperprolactinemic males: a comparison with bromocriptine.** *Eur J Endocrinol* 1998, **138:**286-293.
7. Di Sarno A, Landi ML, Cappabianca P, Di Salle F, Rossi FW, Pivonello R, Di Somma C, Faggiano A, Lombardi G, Colao A: **Resistance to cabergoline as compared with bromocriptine in hyperprolactinemia: prevalence, clinical definition, and therapeutic strategy.** *J Clin Endocrinol Metab* 2001, **86:**5256-5261.
8. Di Somma C, Colao A, Di Sarno A, Klain M, Landi ML, Facciolli G, Pivonello R, Panza N, Salvatore M, Lombardi G: **Bone marker and bone density responses to dopamine agonist therapy in hyperprolactinemic males.** *Journal of Clinical Endocrinology & Metabolism* 1998, **83:**807-813.
9. Hildebrandt G, Bauer T, Stracke H, Fassbender WJ, Mueller HW, Agnoli AL, Federlin K, Roosen K: **Surgery, dopamine agonist therapy of combined treatment--results in prolactinoma patients after a 12 month follow-up.** *Zentralbl Neurochir* 1992, **53:**123-134.
10. Hirahara F, Andoh N, Sawai K, Hirabuki T, Uemura T, Minaguchi H: **Hyperprolactinemic recurrent miscarriage and results of randomized bromocriptine treatment trials.** *Fertility and Sterility* 1998, **70:**246-252.
11. Homburg R, West C, Brownell J, Jacobs HS: **A double-blind study comparing a new non-ergot, long-acting dopamine agonist, CV 205-502, with bromocriptine in women with hyperprolactinaemia.** *Clinical endocrinology* 1990, **32:**565-571.
12. Jeffcoate WJ, Pound N, Sturrock ND, Lambourne J: **Long-term follow-up of patients with hyperprolactinaemia.[see comment].** *Clin Endocrinol (Oxf)* 1996, **45:**299-303.
13. Lappohn RE, van de Wiel HB, Brownell J: **The effect of two dopaminergic drugs on menstrual function and psychological state in hyperprolactinemia.** *Fertil Steril* 1992, **58:**321-327.
14. Mahmood IH, Al-Husaynei AJ, Mohamad SH: **Comparative effects of bromocriptine and cabergoline on serum prolactin levels, liver and kidney function tests in hyperprolactinemic women.** *Pak J Med Sci* 2010, **26:**255-260.
15. Mattei AM, Severini V, Crosignani PG: **Natural history of hyperprolactinemia.** *Annals of the New York Academy of Sciences* 1991, **626:**130-136.
16. Motazedian S, Babakhani L, Fereshtehnejad SM, Mojtahedi K: **A comparison of bromocriptine & cabergoline on fertility outcome of hyperprolactinemic infertile women undergoing intrauterine insemination.** *Indian Journal of Medical Research* 2010, **131:**670-674.
17. Pascal-Vigneron V, Weryha G, Bosc M, Leclere J: **[Hyperprolactinemic amenorrhea:treatment with cabergoline versus bromocriptine. Results of a national multicenter randomized double-blind study].** *Presse Medicale* 1995, **24:**753-757.
18. Perrin G, Treluyer C, Trouillas J, Sassolas G, Goutelle A: **Surgical outcome and pathological effects of bromocriptine preoperative treatment in prolactinomas.** *Pathol Res Pract* 1991, **187:**587-592.
19. Pinzone JJ, Katznelson L, Danila DC, Pauler DK, Miller CS, Klibanski A: **Primary medical therapy of micro- and macroprolactinomas in men.[see comment].** *J Clin Endocrinol Metab* 2000, **85:**3053-3057.

20. Rush S, Donahue B, Cooper P, Lee C, Persky M, Newall J: **Prolactin reduction after combined therapy for prolactin macroadenomas.** *Neurosurgery* 1991, **28:**502-505.
21. Samaan NA, Schultz PN, Leavens TA, Leavens ME, Lee YY: **Pregnancy after treatment in patients with prolactinoma: operation versus bromocriptine.** *Am J Obstet Gynecol* 1986, **155:**1300-1305.
22. Sartorio A, Conti A, Ambrosi B, Muratori M, Morabito F, Faglia G: **Osteocalcin levels in patients with microprolactinoma before and during medical treatment.** *J Endocrinol Invest* 1990, **13:**419-422.
23. Shih CJ, Tsou CK, Chiu WT, Tsai SH: **Management of prolactin-secreting pituitary adenomas with surgery and bromocriptine.** *Southeast Asian Journal of Surgery* 1983, **6:**38-46.
24. Sluijmer AV, Lappohn RE: **Clinical history and outcome of 59 patients with idiopathic hyperprolactinemia.** *Fertility and Sterility* 1992, **58:**72-77.
25. Torres I, Carral F, Vilchez F, GavilÃ¡n I, Aguilar M: **Clinical, radiologic, and follow-up findings in patients with macroprolactinoma.** *Endocrinologist* 2006, **16:**241-244.
26. Touraine P, Plu-Bureau G, Beji C, Mauvais-Jarvis P, Kuttenn F: **Long-term follow-up of 246 hyperprolactinemic patients.** *Acta Obstetricia et Gynecologica Scandinavica* 2001, **80:**162-168.
27. van der Heijden PF, de Wit W, Brownell J, Schoemaker J, Rolland R: **CV 205-502, a new dopamine agonist, versus bromocriptine in the treatment of hyperprolactinaemia.** *Eur J Obstet Gynecol Reprod Biol* 1991, **40:**111-118.
28. Verhelst JA, Froud AL, Touzel R, Wass JA, Besser GM, Grossman AB: **Acute and long-term effects of once-daily oral bromocriptine and a new long-acting non-ergot dopamine agonist, quinagolide, in the treatment of hyperprolactinemia: a double-blind study.** *Acta Endocrinol (Copenh)* 1991, **125:**385-391.
29. Webster J, Piscitelli G, Polli A, Ferrari CI, Ismail I, Scanlon MF: **A comparison of cabergoline and bromocriptine in the treatment of hyperprolactinemic amenorrhea. Cabergoline Comparative Study Group.** *N Engl J Med* 1994, **331:**904-909.
30. Biswas M, Smith J, Jadon D, McEwan P, Rees DA, Evans LM, Scanlon MF, Davies JS: **Long-term remission following withdrawal of dopamine agonist therapy in subjects with microprolactinomas.** *Clinical endocrinology* 2005, **63:**26-31.
31. Cannavo S, Curto L, Squadrito S, Almoto B, Vieni A, Trimarchi F: **Cabergoline: a first-choice treatment in patients with previously untreated prolactin-secreting pituitary adenoma.** *Journal of endocrinological investigation* 1999, **22:**354-359.
32. Ciccarelli E, Grottoli S, Razzore P, Gaia D, Bertagna A, Cirillo S, Cammarota T, Camanni M, Camanni F: **Long-term treatment with cabergoline, a new long-lasting ergoline derivate, in idiopathic or tumorous hyperprolactinaemia and outcome of drug-induced pregnancy.** *Journal of endocrinological investigation* 1997, **20:**547-551.
33. Colao A, Di Sarno A, Cappabianca P, Di Somma C, Pivonello R, Lombardi G: **Withdrawal of long-term cabergoline therapy for tumoral and nontumoral hyperprolactinemia.[see comment].** *N Engl J Med* 2003, **349:**2023-2033.
34. Corenblum B, Taylor PJ: **Idiopathic hyperprolactinemia may include a distinct entity with a natural history different from that of prolactin adenomas.** *Fertility & Sterility* 1988, **49:**544-546.
35. Di Sarno A, Landi ML, Marzullo P, Di Somma C, Pivonello R, Cerbone G, Lombardi G, Colao A: **The effect of quinagolide and cabergoline, two selective dopamine receptor type 2 agonists, in the treatment of prolactinomas.** *Clinical endocrinology* 2000, **53:**53-60.
36. Eversmann T, Fahlbusch R, Rjosk HK, von Werder K: **Persisting suppression of prolactin secretion after long-term treatment with bromocriptine in patients with prolactinomas.** *Acta Endocrinol (Copenh)* 1979, **92:**413-427.
37. Hancock KW, Scott JS, Lamb JT, Gibson RM, Chapman C: **Long term suppression of prolactin concentrations after bromocriptine induced regression of pituitary prolactinomas.** *Br Med J (Clin Res Ed)* 1985, **290:**117-118.
38. Johnston DG, Prescott RW, Kendall-Taylor P, Hall K, Crombie AL, Hall R, McGregor A, Watson MJ, Cook DB: **Hyperprolactinemia. Long-term effects of bromocriptine.** *Am J Med* 1983, **75:**868-874.

39. Kharlip J, Salvatori R, Yenokyan G, Wand GS: **Recurrence of hyperprolactinemia after withdrawal of long-term cabergoline therapy.[see comment].** *J Clin Endocrinol Metab* 2009, **94:**2428-2436.
40. Liuzzi A, Dallabonzana D, Oppizzi G, Verde GG, Cozzi R, Chiodini P, Luccarelli G: **Low doses of dopamine agonists in the long-term treatment of macroprolactinomas.** *The New England journal of medicine* 1985, **313:**656-659.
41. Mattei AM, Ferrari C, Ragni G, Benco R, Picciotti MC, Rampini P, Caldara R, Crosignani PG: **Serum prolactin and ovarian function after discontinuation of drug treatment for hyperprolactinaemia: a study with bromocriptine and metergoline.** *British journal of obstetrics and gynaecology* 1984, **91:**244-250.
42. Moriondo P, Travaglini P, Nissim M, Conti A, Faglia G: **Bromocriptine treatment of microprolactinomas: evidence of stable prolactin decrease after drug withdrawal.** *The Journal of clinical endocrinology and metabolism* 1985, **60:**764-772.
43. Muratori M, Arosio M, Gambino G, Romano C, Biella O, Faglia G: **Use of cabergoline in the long-term treatment of hyperprolactinemic and acromegalic patients.** *Journal of endocrinological investigation* 1997, **20:**537-546.
44. Passos VQ, Souza JJ, Musolino NR, Bronstein MD: **Long-term follow-up of prolactinomas: normoprolactinemia after bromocriptine withdrawal.** *J Clin Endocrinol Metab* 2002, **87:**3578-3582.
45. Tartagni M, Nicastri PL, Diaferia A, Di Gesu I, Loizzi P: **Long-term follow-up of women with amenorrhea-galactorrhea treated with bromocriptine.** *Clinical & Experimental Obstetrics & Gynecology* 1995, **22:**301-306.
46. van 't Verlaat JW, Croughs RJ: **Withdrawal of bromocriptine after long-term therapy for macroprolactinomas; effect on plasma prolactin and tumour size.[see comment].** *Clin Endocrinol (Oxf)* 1991, **34:**175-178.
47. Winkelmann W, Allolio B, Deuss U, Heesen D, Kaulen D: **Persisting normoprolactinemia after withdrawal of bromocriptine long-term therapy in patients with prolactinomas.** *Macleod RM, Thorner MO, Scapagnini U, eds Basic and clinical correlates* 1985**:**817-822.
48. Wu ZB, Yu CJ, Su ZP, Zhuge QC, Wu JS, Zheng WM: **Bromocriptine treatment of invasive giant prolactinomas involving the cavernous sinus: results of a long-term follow up.** *Journal of Neurosurgery* 2006, **104:**54-61.
49. Zarate A, Canales ES, Cano C, Pilonieta CJ: **Follow-up of patients with prolactinomas after discontinuation of long-term therapy with bromocriptine.** *Acta Endocrinologica* 1983, **104:**139-142.
50. Al-Suleiman SA, Najashi S, Rahman J, Rahman MS: **Outcome of treatment with bromocriptine in patients with hyperprolactinaemia.** *Australian and New Zealand Journal of Obstetrics and Gynaecology* 1989, **29:**176-179.
51. Bergh T, Nillius J, Wide L: **Bromocriptine treatment of 42 hyperprolactinaemic women with secondary amenorrhoea.** *Acta Endocrinologica* 1978, **88:**435-451.
52. Brue T, Lancranjan I, Louvet JP, Dewailly D, Roger P, Jaquet P: **A long-acting repeatable form of bromocriptine as long-term treatment of prolactin-secreting macroadenomas: a multicenter study.** *Fertility & Sterility* 1992, **57:**74-80.
53. Cannavo S, De Natale R, Curto L, Li Calzi L, Trimarchi F: **Effectiveness of computer-assisted perimetry in the follow-up of patients with pituitary microadenoma responsive to medical treatment.** *Clinical endocrinology* 1992, **37:**157-161.
54. Chattopadhyay A, Bhansali A, Masoodi SR: **Long-term efficacy of bromocriptine in macroprolactinomas and giant prolactinomas in men.** *Pituitary* 2005, **8:**147-154.
55. Corenblum B, Taylor PJ: **Long-term follow-up of hyperprolactinemic women treated with bromocriptine.** *Fertility & Sterility* 1983, **40:**596-599.
56. Espinos JJ, Rodriguez-Espinosa J, Webb SM, Calaf-Alsina J: **Long-acting repeatable bromocriptine in the treatment of patients with microprolactinoma intolerant or resistant to oral dopaminergics.** *Fertility & Sterility* 1994, **62:**926-931.
57. Essais O, Bouguerra R, Hamzaoui J, Marrakchi Z, Hadjri S, Chamakhi S, Zidi B, Ben Slama C: **Efficacy and safety of bromocriptine in the treatment of macroprolactinomas.** *Annales d Endocrinologie* 2002, **63:**524-531.

58. Falsetti L, Zanagnolo V, Gastaldi A: **A retrospective study of idiopathic hyperprolactinemias.** *Current Therapeutic Research - Clinical and Experimental* 1988, **43:**1063-1072.
59. Fletes Rabago VM, Torres Farias S, Dominguez Jimenez A, Padilla Ruiz R: **[Alternative to bromocriptine (BEC) management in patients with prolactinoma and intolerance to oral BEC].** *Ginecologia y Obstetricia de Mexico* 1991, **59:**283-288.
60. Greenspan SL, Oppenheim DS, Klibanski A: **Importance of gonadal steroids to bone mass in men with hyperprolactinemic hypogonadism.** *Annals of Internal Medicine* 1989, **110:**526-531.
61. Haase R, Jaspers C, Schulte HM, Lancranja I, Pfingsten H, Orri-Fend M, Reinwein D, Benker G: **Control of prolactin-secreting macroadenomas with parenteral, long-acting bromocriptine in 30 patients treated for up to 3 years.** *Clinical endocrinology* 1993, **38:**165-176.
62. Holtkamp W, Nagel GA: **Bromocriptine with chemotherapy resistant, metastatic breast cancer - Results of the AIO-Study GO-MC-BROMO 2/82.** *Onkologie* 1988, **11:**121-127.
63. Jamrozik SI, Bennet AP, James-Deidier A, Tremollieres F, Saint-Martin F, Dumoulin S, Valat-Coustols M, de Glisezinski I, Tremoulet M, Manelfe C, Louvet JP: **Treatment with long acting repeatable bromocriptine (Parlodel-LAR*) in patients with macroprolactinomas: long-term study in 29 patients.** *Journal of endocrinological investigation* 1996, **19:**472-479.
64. Lengyel AM, Mussio W, Imamura P, Vieira JG, Lancranjan I: **Long-acting injectable bromocriptine (Parlodel LAR) in the chronic treatment of prolactin-secreting macroadenomas.** *Fertility & Sterility* 1993, **59:**980-987.
65. Lin SQ: **[Experiences with bromocriptine treatment of female infertility due to hyperprolactinemia].** *Chung-Hua Fu Chan Ko Tsa Chih [Chinese Journal of Obstetrics & Gynecology]* 1992, **27:**28-31.
66. Moberg E, af Trampe E, Wersall J, Werner S: **Long-term effects of radiotherapy and bromocriptine treatment in patients with previous surgery for macroprolactinomas.** *Neurosurgery* 1991, **29:**200-204; discussion 204-205.
67. Maraschini C, Moro M, Masala A, Toja P, Alagna S, Brunani A, Rovasio PP, Ginanni A, Lancranjan I, Cavagnini F: **Chronic treatment with parlodel LAR of patients with prolactin-secreting tumours. Different responsiveness of micro- and macroprolactinomas.** *Acta Endocrinologica* 1991, **125:**494-501.
68. Merola B, Colao A, Caruso E, Sarnacchiaro F, Lancranjan I, Lombardi G, Schettini G: **Effectiveness and long-term tolerability of the slow release oral form of bromocriptine on tumoral and non-tumoral hyperprolactinemia.** *Journal of endocrinological investigation* 1992, **15:**173-176.
69. Molitch ME, Elton RL, Blackwell RE, Caldwell B, Chang RJ, Jaffe R, Joplin G, Robbins RJ, Tyson J, Thorner MO: **Bromocriptine as primary therapy for prolactin-secreting macroadenomas: results of a prospective multicenter study.** *Journal of Clinical Endocrinology & Metabolism* 1985, **60:**698-705.
70. Mornex R, Orgiazzi J, Hugues B, Gagnaire JC, Claustrat B: **Normal pregnancies after treatment of hyperprolactinemia with bromoergocryptine, despite suspected pituitary tumors.** *Journal of Clinical Endocrinology & Metabolism* 1978, **47:**290-295.
71. Moro M, Maraschini C, Toja P, Masala A, Alagna S, Rovasio PP, Ginanni A, Lancranjan I, Cavagnini F: **Comparison between a slow-release oral preparation of bromocriptine and regular bromocriptine in patients with hyperprolactinemia: a double blind, double dummy study.** *Hormone Research* 1991, **35:**137-141.
72. Paoletti AM, Cagnacci A, Mais V, Ajossa S, Guerriero S, Murgia C, Depau GF, Serra GG, Melis GB: **Shrinkage of pituitary PRL-secernent adenoma after short-term treatment with bromocriptine long-acting repeatable injections.** *Clinical and Experimental Obstetrics and Gynecology* 1994, **21:**124-128.
73. Rasmussen C, Larsson SG, Bergh T: **Long-term radiographic follow-up of the sella turcica in hyperprolactinaemic women.** *Australian & New Zealand Journal of Obstetrics & Gynaecology* 1990, **30:**257-264.
74. Schettini G, Lombardi G, Merola B, Colao A, Miletto P, Caruso E, Lancranjan I: **Rapid and long-lasting suppression of prolactin secretion and shrinkage of prolactinomas after injection of long-acting repeatable form of bromocriptine (Parlodel LAR).** *Clinical endocrinology* 1990, **33:**161-169.

75. Skrabanek P, McDonald D, De Valera E: **Plasma prolactin in amenorrhoea, infertility, and other disorders: A retrospective study of 608 patients.** *Irish Journal of Medical Science* 1980, **149:**236-245.
76. Spark RF, Baker R, Bienfang DC, Bergland R: **Bromocriptine reduces pituitary tumor size and hypersection. Requiem for pituitary surgery?** *JAMA* 1982, **247:**311-316.
77. Thorner MO, Besser GM: **Bromocriptine treatment of hyperprolactinaemic hypogonadism.** *Acta Endocrinologica Supplementum* 1978, **216:**131-146.
78. Tsagarakis S, Tsiganou E, Tzavara I, Nikolou H, Thalassinos N: **Effectiveness of a long-acting injectable form of bromocriptine in patients with prolactin and growth hormone secreting macroadenomas.** *Clinical endocrinology* 1995, **42:**593-599.
79. van 't Verlaat JW, Croughs RJ, Hendriks MJ, Bosma NJ, Nortier JW, Thijssen JH: **Bromocriptine treatment of prolactin secreting macroadenomas: a radiological, ophthalmological and endocrinological study.** *Acta Endocrinologica* 1986, **112:**487-493.
80. Walsh JP, Pullan PT: **Hyperprolactinaemia in males: a heterogeneous disorder.** *Australian & New Zealand Journal of Medicine* 1997, **27:**385-390.
81. Wass JA, Williams J, Charlesworth M, Kingsley DP, Halliday AM, Doniach I, Rees LH, McDonald WI, Besser GM: **Bromocriptine in management of large pituitary tumours.** *British Medical Journal Clinical Research Ed* 1982, **284:**1908-1911.
82. Weingrill CO, Mussio W, Moraes CR, Portes E, Castro RC, Lengyel AM: **Long-acting oral bromocriptine (Parlodel SRO) in the treatment of hyperprolactinemia.** *Fertility & Sterility* 1992, **57:**331-335.
83. Yang MS, Hong JW, Lee SK, Lee EJ, Kim SH: **Clinical management and outcome of 36 invasive prolactinomas treated with dopamine agonist.** *J Neurooncol* 2011, **104:**195-204.
84. Bhansali A, Walia R, Dutta P, Khandelwal N, Sialy R, Bhadada S: **Efficacy of cabergoline on rapid escalation of dose in men with macroprolactinomas.** *Indian Journal of Medical Research* 2010, **131:**530-535.
85. Biller BMK, Molitch ME, Vance ML, Cannistraro KB, Davis KR, Simons JA, Schoenfelder JR, Klibanski A: **Treatment of prolactin-secreting macroadenomas with the once-weekly dopamine agonist cabergoline.** *Journal of Clinical Endocrinology and Metabolism* 1996, **81:**2338-2343.
86. Bolko P, Jaskula M, Wasko R, Wolun M, Sowinski J: **The assessment of cabergoline efficacy and tolerability in patients with pituitary prolactinoma type.** *Polskie Archiwum Medycyny Wewnetrznej* 2003, **109:**489-495.
87. Buyukbayrak EE, Karageyim Karsidag AY, Kars B, Balcik O, Pirimoglu M, Unal O, Turan C: **Effectiveness of short-term maintenance treatment with cabergoline in microadenoma-related and idiopathic hyperprolactinemia.** *Arch Gynecol Obstet* 2010, **282:**561-566.
88. Cho E-H, Lee SA, Chung JY, Koh EH, Cho YH, Kim JH, Kim CJ, Kim M-S: **Efficacy and safety of cabergoline as first line treatment for invasive giant prolactinoma.** *J Korean Med Sci* 2009, **24:**874-878.
89. Ciccarelli E, Giusti M, Miola C, Potenzoni F, Sghedoni D, Camanni F, Giordano G: **Effectiveness and tolerability of long term treatment with cabergoline, a new long-lasting ergoline derivative, in hyperprolactinemic patients.** *Journal of Clinical Endocrinology & Metabolism* 1989, **69:**725-728.
90. Colao A, Di Sarno A, Landi ML, Cirillo S, Sarnacchiaro F, Facciolli G, Pivonello R, Cataldi M, Merola B, Annunziato L, Lombardi G: **Long-term and low-dose treatment with cabergoline induces macroprolactinoma shrinkage.** *Journal of Clinical Endocrinology & Metabolism* 1997, **82:**3574-3579.
91. Colao A, Di Sarno A, Landi ML, Scavuzzo F, Cappabianca P, Pivonello R, Volpe R, Di Salle F, Cirillo S, Annunziato L, Lombardi G: **Macroprolactinoma shrinkage during cabergoline treatment is greater in naive patients than in patients pretreated with other dopamine agonists: a prospective study in 110 patients.** *Journal of Clinical Endocrinology & Metabolism* 2000, **85:**2247-2252.
92. Colao A, Vitale G, Di Sarno A, Spiezia S, Guerra E, Ciccarelli A, Lombardi G: **Prolactin and prostate hypertrophy: a pilot observational, prospective, case-control study in men with prolactinoma.** *Journal of Clinical Endocrinology & Metabolism* 2004, **89:**2770-2775.

93. Colao A, Vitale G, Cappabianca P, Briganti F, Ciccarelli A, De Rosa M, Zarrilli S, Lombardi G: **Outcome of cabergoline treatment in men with prolactinoma: effects of a 24-month treatment on prolactin levels, tumor mass, recovery of pituitary function, and semen analysis.** *Journal of Clinical Endocrinology & Metabolism* 2004, **89:**1704-1711.
94. Corsello SM, Ubertini G, Altomare M, Lovicu RM, Migneco MG, Rota CA, Colosimo C: **Giant prolactinomas in men: Efficacy of cabergoline treatment.** *Clinical endocrinology* 2003, **58:**662-670.
95. De Bellis A, Colao A, Savoia A, Coronella C, Pasquali D, Conte M, Pivonello R, Bellastella A, Sinisi AA, Bizzarro A, et al: **Effect of long-term cabergoline therapy on the immunological pattern and pituitary function of patients with idiopathic hyperprolactinaemia positive for antipituitary antibodies.** *Clinical endocrinology* 2008, **69:**285-291.
96. De Rosa M, Ciccarelli A, Zarrilli S, Guerra E, Gaccione M, Di Sarno A, Lombardi G, Colao A: **The treatment with cabergoline for 24 month normalizes the quality of seminal fluid in hyperprolactinaemic males.** *Clinical endocrinology* 2006, **64:**307-313.
97. Delgrange E, Maiter D, Donckier J: **Effects of the dopamine agonist cabergoline in patients with prolactinoma intolerant or resistant to bromocriptine.** *Eur J Endocrinol* 1996, **134:**454-456.
98. Ferrari C, Paracchi A, Mattei AM, De Vincentiis S, D'Alberton A, Crosignani P: **Cabergoline in the long-term therapy of hyperprolactinemic disorders.** *Acta Endocrinologica* 1992, **126:**489-494.
99. Ferrari CI, Abs R, Bevan JS, Brabant G, Ciccarelli E, Motta T, Mucci M, Muratori M, Musatti L, Verbessem G, Scanlon MF: **Treatment of macroprolactinoma with cabergoline: a study of 85 patients.** *Clinical endocrinology* 1997, **46:**409-413.
100. Ono M, Miki N, Kawamata T, Makino R, Amano K, Seki T, Kubo O, Hori T, Takano K: **Prospective study of high-dose cabergoline treatment of prolactinomas in 150 patients.[see comment].** *Journal of Clinical Endocrinology & Metabolism* 2008, **93:**4721-4727.
101. Ono M, Miki N, Amano K, Kawamata T, Seki T, Makino R, Takano K, Izumi S-i, Okada Y, Hori T: **Individualized high-dose cabergoline therapy for hyperprolactinemic infertility in women with micro- and macroprolactinomas.** *J Clin Endocrinol Metab* 2010, **95:**2672-2679.
102. Pontikides N, Krassas GE, Nikopoulou E, Kaltsas T: **Cabergoline as a first-line treatment in newly diagnosed macroprolactinomas.** *Pituitary* 2000, **2:**277-281.
103. Raverot G, Jacob M, Jouanneau E, Delemer B, Vighetto A, Pugeat M, Borson-Chazot F: **Secondary deterioration of visual field during cabergoline treatment for macroprolactinoma.** *Clinical endocrinology* 2009, **70:**588-592.
104. Shimon I, Benbassat C, Hadani M: **Effectiveness of long-term cabergoline treatment for giant prolactinoma: study of 12 men.** *Eur J Endocrinol* 2007, **156:**225-231.
105. Sinha S, Sharma BS, Mahapatra AK: **Microsurgical management of prolactinomas - clinical and hormonal outcome in a series of 172 cases.** *Neurol India* 2011, **59:**532-536.
106. Walia R, Bhansali A, Dutta P, Khandelwal N, Sialy R, Bhadada S: **Recovery pattern of hypothalamo-pituitary-testicular axis in patients with macroprolactinomas after treatment with cabergoline.** *Indian Journal of Medical Research* 2011, **134:**314-319.
107. Webster J, Piscitelli G, Polli A, D'Alberton A, Falsetti L, Ferrari C, Fioretti P, Giordano G, L'Hermite M, Ciccarelli E, et al.: **The efficacy and tolerability of long-term cabergoline therapy in hyperprolactinaemic disorders: an open, uncontrolled, multicentre study. European Multicentre Cabergoline Study Group.** *Clinical endocrinology* 1993, **39:**323-329.
108. Colao A, De Rosa M, Sarnacchiaro F, Di Sarno A, Landi ML, Iervolino E, Zarrilli S, Merola B, Lombardi G: **Chronic treatment with CV 205-502 restores the gonadal function in hyperprolactinemic males.** *Eur J Endocrinol* 1996, **135:**548-552.
109. Duranteau L, Chanson P, Lavoinne A, Horlait S, Lubetzki J, Kuhn JM: **Effect of the new dopaminergic agonist CV 205-502 on plasma prolactin levels and tumour size in bromocriptine-resistant prolactinomas.** *Clinical endocrinology* 1991, **34:**25-29.
110. Kvistborg A, Halse J, Bakke S, Bjoro T, Hansen E, Djoseland O, Brownell J, Jervell J: **Long-term treatment of macroprolactinomas with CV 205-502.** *Acta Endocrinologica* 1993, **128:**301-307.
111. Merola B, Sarnacchiaro F, Colao A, Disomma C, Disarno A, Landi ML, Marzullo P, Panza N, Battista C, Lombardi G: **CV-205-502 in the treatment of utmoral and nontumoral hyperprolactinemic states.** *Biomedicine & Pharmacotherapy* 1994, **48:**167-174.

112. Morange I, Barlier A, Pellegrini I, Brue T, Enjalbert A, Jaquet P: **Prolactinomas resistant to bromocriptine: Long-term efficacy of quinagolide and outcome of pregnancy.** *Eur J Endocrinol* 1996, **135:**413-420.
113. Newman CB, Hurley AM, Kleinberg DL: **Effect of CV 205-502 in hyperprolactinaemic patients intolerant of bromocriptine.** *Clinical endocrinology* 1989, **31:**391-400.
114. Nickelsen T, Jungmann E, Althoff P, Schummdraeger PM, Usadel KH: **Treatment of macroprolactinoma with the new potent nonergot D2-dopamine agonist quinagolide and effects on prolactin levels, pituitary-function, and the renin-aldosterone system- results of a clinical long-term study.** *Arzneimittel-Forschung/Drug Research* 1993, **43-1:**421-425.
115. Rasmussen C, Bergh T, Wide L, Brownell J: **Long-term treatment with a new non-ergot long-acting dopamine agonist, CV 205-502, in women with hyperprolactinaemia.** *Clinical endocrinology* 1988, **29:**271-279.
116. Rohmer V, Freneau E, Morange I, Simonetta C: **Efficacy of quinagolide in resistance to dopamine agonists: results of a multicenter study. Club de l'Hypophyse.** *Annales d Endocrinologie* 2000, **61:**411-417.
117. Shoham Z, Homburg R, Jacobs HS: **CV 205-502--effectiveness, tolerability, and safety over 24-month study.** *Fertility & Sterility* 1991, **55:**501-506.
118. van der Heijden PF, Lappohn RE, Corbey RS, de Goeij WB, Brownell J, Rolland R: **The effectiveness, safety, and tolerability of CV 205-502 in hyperprolactinemic women: a 12-month study.** *Fertility & Sterility* 1989, **52:**574-579.
119. van der Lely AJ, Brownell J, Lamberts SW: **The efficacy and tolerability of CV 205-502 (a nonergot dopaminergic drug) in macroprolactinoma patients and in prolactinoma patients intolerant to bromocriptine.** *Journal of Clinical Endocrinology & Metabolism* 1991, **72:**1136-1141.
120. Vance ML, Lipper M, Klibanski A, Biller BMK, Samaan NA, Molitch ME: **Treatment of prolactin-secreting pituitary macroadenomas with the long-acting non-ergot dopamine agonist CV 205-502.** *Annals of Internal Medicine* 1990, **112:**668-673.
121. Vilar L, Burke CW: **Quinagolide efficacy and tolerability in hyperprolactinaemic patients who are resistant to or intolerant of bromocriptine.[see comment].** *Clinical endocrinology* 1994, **41:**821-826.
122. Freda PU, Andreadis CI, Khandji AG, Khoury M, Bruce JN, Jacobs TP, Wardlaw SL: **Long-term treatment of prolactin-secreting macroadenomas with pergolide.** *Journal of Clinical Endocrinology & Metabolism* 2000, **85:**8-13.
123. Jaspers C, Benker G, Reinwein D: **Treatment of prolactinoma patients with the new non-ergot dopamine agonist roxindol: First results.** *Clinical Investigator* 1994, **72:**451-456.
124. Orrego JJ, Chandler WF, Barkan AL: **Pergolide as primary therapy for macroprolactinomas.** *Pituitary* 2000, **3:**251-256.
125. Sibal L, Ugwu P, Kendall-Taylor P, Ball SG, James RA, Pearce SH, Hall K, Quinton R: **Medical therapy of macroprolactinomas in males: I. Prevalence of hypopituitarism at diagnosis. II. Proportion of cases exhibiting recovery of pituitary function.** *Pituitary* 2002, **5:**243-246.
126. Verde G, Chiodini PG, Liuzzi A, Cozzi R, Favales F, Botalla L, Spelta B, Dalla Bonzana D, Rainer E, Horowski R: **Effectiveness of the dopamine agonist lisuride in the treatment of acromegaly and pathological hyperprolactinemic states.** *Journal of endocrinological investigation* 1980, **3:**405-414.
127. Jezkova J, Hana V, Krsek M, Weiss V, Vladyka V, Liscak R, Vymazal J, Pecen L, Marek J: **Use of the Leksell gamma knife in the treatment of prolactinoma patients.** *Clinical endocrinology* 2009, **70:**732-741.
128. Pan L, Zhang N, Wang EM, Wang BJ, Dai JZ, Cai PW: **Gamma knife radiosurgery as a primary treatment for prolactinomas.** *JOURNAL OF NEUROSURGERY* 2000, **93:**10-13.
129. Pouratian N, Sheehan J, Jagannathan J, Laws ER, Jr., Steiner L, Vance ML: **Gamma knife radiosurgery for medically and surgically refractory prolactinomas.** *Neurosurgery* 2006, **59:**255-266; discussion 255-266.
130. Sun DQ, Cheng JJ, Frazier JL, Batra S, Wand G, Kleinberg LR, Rigamonti D, Quinones-Hinojosa A, Salvatori R, Lim M: **Treatment of pituitary adenomas using radiosurgery and radiotherapy: a single center experience and review of literature.** *Neurosurg Rev* 2010, **34:**181-189.

131. Tsagarakis S, Grossman A, Plowman PN, Jones AE, Touzel R, Rees LH, Wass JA, Besser GM: **Megavoltage pituitary irradiation in the management of prolactinomas: long-term follow-up.** *Clinical endocrinology* 1991, **34:**399-406.
132. Yoon SC, Suh TS, Jang HS, Chung SM, Kim YS, Ryu MR, Choi KH, Son HY, Kim MC, Shinn KS: **Clinical results of 24 pituitary macroadenomas with linac-based stereotactic radiosurgery.** *International Journal of Radiation Oncology Biology Physics* 1998, **41:**849-853.
133. Zhang N, Pan L, Dai J, Wang B, Wang E, Zhang W, Cai P: **Gamma Knife radiosurgery as a primary surgical treatment for hypersecreting pituitary adenomas.** *Stereotactic & Functional Neurosurgery* 2000, **75:**123-128.
134. Zierhut D, Flentje M, Adolph J, Erdmann J, Raue F, Wannenmacher M: **EXTERNAL RADIOTHERAPY OF PITUITARY-ADENOMAS.** *International Journal of Radiation Oncology Biology Physics* 1995, **33:**307-314.
135. Kelly WF, Mashiter K, Doyle FH, Banks LM, Joplin GF: **Treatment of prolactin-secreting pituitary tumours in young women by needle implantation of radioactive yttrium.** *Quarterly Journal of Medicine* 1978, **47:**473-493.
136. Littley MD, Shalet SM, Reid H, Beardwell CG, Sutton ML: **The effect of external pituitary irradiation on elevated serum prolactin levels in patients with pituitary macroadenomas.** *Quarterly Journal of Medicine* 1991, **81:**985-998.
137. Rudoler SB, Ruffer JE, Gennarelli TA, Savino PJ, Fowble BF: **Long-term results of radiotherapy for prolactin-secreting pituitary macroadenomas.** *Radiation Oncology Investigations* 1996, **4:**185-191.
138. Tanaka S, Link MJ, Brown PD, Stafford SL, Young WF, Jr., Pollock BE: **Gamma knife radiosurgery for patients with prolactin-secreting pituitary adenomas.** *World Neurosurg* 2010, **74:**147-152.
139. Amar AP, Couldwell WT, Chen JCT, Weiss MH: **Predictive value of serum prolactin levels measured immediately after transsphenoidal surgery.** *Journal of Neurosurgery* 2002, **97:**307-314.
140. Babey M, Sahli R, Vajtai I, Andres RH, Seiler RW: **Pituitary surgery for small prolactinomas as an alternative to treatment with dopamine agonists.** *Pituitary* 2011, **14:**222-230.
141. Barbarino A, De Marinis L, Anile C, Menini E, Merlini G, Maira G: **Dopaminergic mechanisms regulating prolactin secretion in patients with prolactin-secreting pituitary adenoma. Long-term studies after selective transsphenoidal surgery.** *Metabolism: Clinical & Experimental* 1982, **31:**1100-1104.
142. Charpentier G, de Plunkett T, Jedynak P, Peillon F, Le Gentil P, Racadot J, Visot A, Derome P: **Surgical treatment of prolactinomas. Short- and long-term results, prognostic factors.** *Hormone Research* 1985, **22:**222-227.
143. Dusick JR, Fatemi N, Mattozo C, McArthur D, Cohan P, Wang C, Swerdloff RS, Kelly DF: **Pituitary function after endonasal surgery for nonadenomatous parasellar tumors: Rathke's cleft cysts, craniopharyngiomas, and meningiomas.** *Surgical Neurology* 2008, **70:**482-490.
144. Fatemi N, Dusick JR, Mattozo C, McArthur DL, Cohan P, Boscardin J, Wang C, Swerdloff RS, Kelly DF: **Pituitary hormonal loss and recovery after transsphenoidal adenoma removal.** *Neurosurgery* 2008, **63:**709-718.
145. Feigenbaum SL, Downey DE, Wilson CB, Jaffe RB: **Transsphenoidal pituitary resection for preoperative diagnosis of prolactin-secreting pituitary adenoma in women: long term follow-up.** *Journal of Clinical Endocrinology & Metabolism* 1996, **81:**1711-1719.
146. Gokalp HZ, Deda H, Attar A, Ugur HC, Arasil E, Egemen N: **The neurosurgical management of prolactinomas.** *Journal of Neurosurgical Sciences* 2000, **44:**128-132.
147. Guidetti B, Fraioli B, Cantore GP: **Results of surgical management of 319 pituitary adenomas.** *Acta Neurochirurgica* 1987, **85:**117-124.
148. Hamilton DK, Vance ML, Boulos PT, Laws ER: **Surgical outcomes in hyporesponsive prolactinomas: analysis of patients with resistance or intolerance to dopamine agonists.** *Pituitary* 2005, **8:**53-60.
149. Hirohata T, Uozumi T, Mukada K, Arita K, Kurisu K, Yano T, Takechi A, Onda J: **Influence of pregnancy on the serum prolactin level following prolactinoma surgery.** *Acta Endocrinologica* 1991, **125:**259-267.

150. Laws ER, Jr., Fode NC, Redmond MJ: **Transsphenoidal surgery following unsuccessful prior therapy. An assessment of benefits and risks in 158 patients.** *Journal of Neurosurgery* 1985, **63:**823-829.
151. Maira G, Anile C, De Marinis L, Barbarino A: **Prolactin-secreting adenomas: Surgical results and long-term follow-up.** *Neurosurgery* 1989, **24:**736-743.
152. Massoud F, Serri O, Hardy J, Somma M, Beauregard H: **Transsphenoidal adenomectomy for microprolactinomas: 10 to 20 years of follow-up.** *Surgical Neurology* 1996, **45:**341-346.
153. Nakagawa H, Iwatsuki K, Yamada M, Hagiwara Y, Moriuchi S, Kadota T: **Latent prolactinoma on MRI-selective venous sampling and trans-sphenoidal microsurgical treatment.** *Neurological Research* 2001, **23:**691-696.
154. Pandey P, Ojha BK, Mahapatra AK: **Pediatric pituitary adenoma: A series of 42 patients.** *Journal of Clinical Neuroscience* 2005, **12:**124-127.
155. Qu X, Wang M, Wang GD, Han T, Mou CZ, Han LZ, Jiang M, Qu YM, Zhang MA, Pang Q, Xu GM: **Surgical outcomes and prognostic factors of transsphenoidal surgery for prolactinoma in men: a single-center experience with 87 consecutive cases.** *Eur* 2011, **164:**499-504.
156. Rawe SE, Williamson HO, Levine JH, Phansey SA, Hungerford D, Adkins WY: **Prolactinomas: surgical therapy, indications and results.** *Surgical Neurology* 1980, **14:**161-167.
157. Saitoh Y, Mori S, Arita N, Nagatani M, Hayakawa T, Koizumi K, Tanizawa O, Uozumi T, Mogami H: **Treatment of prolactinoma based on the results of transsphenoidal operations.** *Surgical Neurology* 1986, **26:**338-344.
158. Santoro A, Minniti G, Ruggeri A, Esposito V, Jaffrain-Rea ML, Delfini R: **Biochemical remission and recurrence rate of secreting pituitary adenomas after transsphenoidal adenomectomy: long-term endocrinologic follow-up results.** *Surgical Neurology* 2007, **68:**513-518.
159. Scamoni C, Balzarini C, Crivelli G, Dorizzi A: **Treatment and long-term follow-up results of prolactin secreting pituitary adenomas.** *Journal of Neurosurgical Sciences* 1991, **35:**9-16.
160. Shen CC, Wang YC, Wei-Shan H, Chang CS, Sun MH: **Endoscopic endonasal transsphenoidal surgery for pituitary tumors.** *Chinese Medical Journal (Taipei)* 2000, **63:**301-310.
161. Soule SG, Farhi J, Conway GS, Jacobs HS, Powell M: **The outcome of hypophysectomy for prolactinomas in the era of dopamine agonist therapy.[see comment].** *Clinical endocrinology* 1996, **44:**711-716.
162. Webster J, Page MD, Bevan JS, Richards SH, Douglas-Jones AG, Scanlon MF: **Low recurrence rate after partial hypophysectomy for prolactinoma: the predictive value of dynamic prolactin function tests.** *Clinical endocrinology* 1992, **36:**35-44.
163. Wolfsberger S, Czech T, Vierhapper H, Benavente R, Knosp E: **Microprolactinomas in males treated by transsphenoidal surgery.** *Acta Neurochirurgica* 2003, **145:**935-940; discussion 940-931.
164. Zhang HW, Sun W, Yang J, Yan CX, Yu CJ: **Diagnosis and treatment of pituitary microadenoma: report of 80 cases.** *Neurological Research* 2008, **30:**587-593.
165. Jamjoom ZAB, Malabarey T, Jamjoom AHB, Sulimani R, Rahman NU, Sadiq S: **Problems in the management of large prolactin-secreting pituitary adenomas.** *Saudi Medical Journal* 1995, **16:**119-125.
166. Saeki N, Nakamura M, Sunami K, Yamaura A: **Surgical indication after bromocriptine therapy on giant prolactinomas: Effects and limitations of the medical treatment.** *Endocrine Journal* 1998, **45:**529-537.
167. Thompson CJ, Tam NNC, Joyce JM, Leav I, Ho SM: **Gene expression profiling of testosterone and estradiol-17 beta-induced prostatic dysplasia in noble rats and response to the antiestrogen ICI 182,780.** *Endocrinology* 2002, **143:**2093-2105.
168. Thomson JA, Davies DL, McLaren EH, Teasdale GM: **Ten year follow up of microprolactinoma treated by transsphenoidal surgery.** *British Medical Journal* 1994, **309:**1409-1410.
169. Oruckaptan HH, Senmevsim O, Ozcan OE, Ozgen T: **Pituitary adenomas: results of 684 surgically treated patients and review of the literature.** *Surgical Neurology* 2000, **53:**211-219.

170. Rush S, Cooper PR: **Symptom resolution, tumor control, and side effects following postoperative radiotherapy for pituitary macroadenomas.** *International Journal of Radiation Oncology, Biology, Physics* 1997, **37:**1031-1034.
171. Badawy SZ, Marziale JC, Rosenbaum AE, Chang JK, Joy SE: **The long-term effects of pregnancy and bromocriptine treatment on prolactinomas--the value of radiologic studies.** *Early Pregnancy* 1997, **3:**306-311.
172. Berinder K, Hulting AL, Granath F, Hirschberg AL, Akre O: **Parity, pregnancy and neonatal outcomes in women treated for hyperprolactinaemia compared with a control group.** *Clinical endocrinology* 2007, **67:**393-397.
173. Colao A, Abs R, Barcena DG, Chanson P, Paulus W, Kleinberg DL: **Pregnancy outcomes following cabergoline treatment: extended results from a 12-year observational study.** *Clinical endocrinology* 2008, **68:**66-71.
174. Cristiani P, De March A, Bulletti C: **Follow-up of 17 hyperprolactinemic pregnant women by plasma prolactin levels and pituitary politomograms.** *Journal of Gynaecological Endocrinology* 1985, **1:**48-51.
175. Crosignani PG, Mattei AM, Scarduelli C, Cavioni V, Bora cchi P: **Is pregnancy the best treatment for hyperprolactinaemia?** *Human Reproduction* 1989, **4:**910-912.
176. Crosignani PG, Mattei AM, Severini V, Cavioni V, Maggioni P, Testa G: **Long-term effects of time, medical treatment and pregnancy in 176 hyperprolactinemic women.** *European Journal of Obstetrics Gynecology and Reproductive Biology* 1992, **44:**175-180.
177. Godo G, Koloszar S, Szilagyi I, Daru J, Sas M: **Experience relating to pregnancy, lactation, and the afterweaning condition of hyperprolactinemic patients treated with bromocriptine.** *Fertility and Sterility* 1989, **51:**529-531.
178. Holmgren U, Bergstrand G, Hagenfeldt K, Werner S: **Women with prolactinoma--effect of pregnancy and lactation on serum prolactin and on tumour growth.** *Acta Endocrinologica* 1986, **111:**452-459.
179. W. J. Jeffcoate NP, N. D. Sturrock and J. Lambourne": **Long-term follow-up of patients with hyperprolactinaemia.[see comment].** 1996, **45:**299-303.
180. Karunakaran S, Page RCL, Wass JAH: **The effect of the menopause on prolactin levels in patients with hyperprolactinaemia.** *Clinical endocrinology* 2001, **54:**295-300.
181. Kelly WF, Doyle FH, Mashiter K, Banks LM, Gordon H, Joplin GF: **Pregnancies in women with hyperprolactinaemia: clinical course and obstetric complications of 41 pregnancies in 27 women.** *British Journal of Obstetrics & Gynaecology* 1979, **86:**698-705.
182. Kupersmith MJ, Rosenberg C, Kleinberg D: **Visual loss in pregnant women with pituitary adenomas.[see comment].** *Annals of Internal Medicine* 1994, **121:**473-477.
183. Rasmussen C, Bergh T, Nillius SJ, Wide L: **Return of menstruation and normalization of prolactin in hyperprolactinemic women with bromocriptine-induced pregnancy.** *Fertility & Sterility* 1985, **44:**31-34.
184. A. M. Rossi SVaPKH: **Outcome of pregnancies in women with treated or untreated hyperprolactinemia.** 1995, **63:**143-146.
185. Woodhouse NJ, Niles N, McDonald D, McCorkell S: **Prolactin levels in pregnancy: comparison of normal subjects with patients having micro- or macroadenomas after early bromocriptine withdrawal.** *Hormone Research* 1985, **21:**1-9.
186. Zarate A, Canales ES, Alger M, Forsbach G: **The effect of pregnancy and lactation on pituitary prolactin-secreting tumours.** *Acta Endocrinologica* 1979, **92:**407-412.

Search Strategy:

Prolactinoma Agonists

EMBASE/MEDLINE
1. prolactin/bl, se or hyperprolactinemia/ or hyperprolactin*.mp. [mp=title, original title, abstract, name of substance word, subject heading word]
2. exp Adenoma/
3. pituitary neoplasms/ or prolactinoma/
4. 3 or prolactinoma*.mp. or microprolactinoma*.mp. or macroprolactinoma*.mp. [mp=title, original title, abstract, name of substance word, subject heading word]
5. 2 or macroadenoma*.mp. or microadenoma*.mp. [mp=title, original title, abstract, name of substance word, subject heading word]
6. 1 and hypersecreting*.mp. [mp=title, original title, abstract, name of substance word, subject heading word]
7. 1 and 5
8. 4 or 6 or 7
9. ((lactotroph or prl or prolactin) adj (producing or secreting)).mp. [mp=title, original title, abstract, name of substance word, subject heading word]
10. 8 or 9
11. exp Dopamine Agonists/
12. ((dopaminergic or dopamine) adj3 (stimulating or agonist*)).mp. [mp=title, original title, abstract, name of substance word, subject heading word]
13. (abeorphine or acetergamine or adrogolide or aletnamol or amantadine or apocodeine or apomorphine or aripiprazole or bifeprunox or bromocriptine or cabergoline or carmoxirole or ciladopa or dihydrexidine).mp. [mp=title, original title, abstract, name of substance word, subject heading word]
14. (dihydroergocryptine or disulergine or docarpamine or dopamine or dopexamine or dronabinol or epinine).mp. [mp=title, original title, abstract, name of substance word, subject heading word]
15. (fenoldopam or glutamyldopa or ibopamine or indatraline or isomolpan or lisuride or mergocriptine or mesulergine or minaprine or moxiraprine or naxagolide or nolomirole or orotirelin or pardoprunox or pergolide or piribedil or pramipexole or preclamol).mp. [mp=title, original title, abstract, name of substance word, subject heading word]
16. (propylnorapomorphine or quinagolide or quinelorane or quinpirole or roxindole or sibenadet or sumanirole or talixpexole or terguride or vanoxerine).mp. [mp=title, original title, abstract, name of substance word, subject heading word]
17. or/11-16
18. 10 and 17
19. ..l/ 18 hu=y
20. 1 and hyperprolactinemia/dt, su, rt, th
21. 17 and 20
22. ..l/ 21 hu=y
23. 19 or 22
24. exp clinical trials as topic/
25. 23 and (exp cohort studies/ or exp case-control studies/ or cohort*.mp. or observation*.mp. or prospective studies/ or retrospective studies/)
26. 23 and 24
27. limit 23 to (clinical trial, all or clinical trial, phase i or clinical trial, phase ii or clinical trial, phase iii or clinical trial, phase iv or clinical trial or comparative study or controlled clinical trial or evaluation studies or meta analysis or multicenter study or practice guideline or randomized controlled trial)
28. 23 and follow-up studies/ and comparative studies/
29. 25 or 26 or 27 or 28
30. 29 and (outcome*.mp. or treatment outcome/ or follow-up studies/) [mp=title, original title, abstract, name of substance word, subject heading word]
31. (postoperative complications/ or exp radiotherapy/ae) and 29
32. 29 and ae.fs.
33. exp infertility, male/ or exp infertility, female/ or amenorrhea/ or exp headaches/ or exp cranial nerve disorders/ or gynecomastia/ or exp vision disorders/
34. exp Sexual Dysfunction, Physiological/ or exp Sexual Behavior/ or exp Erectile Dysfunction/
35. exp heart valve diseases/ or exp fractures, bone/ or exp bone diseases, metabolic/ or exp menstruation/
36. exp Withholding Treatment/
37. exp pituitary neoplasms/pa
38. or/33-37
39. exp quality of life/

40. (volume or size or growth or enlarge* or withdraw*).mp. [mp=title, original title, abstract, name of substance word, subject heading word]
41. 29 and 38
42. 29 and (39 or 40)
43. 42 or 32 or 30 or 41 or 31
44. from 43 keep 1-540
45. restor*.mp. or recovery of function/ or neoplasm recurrence, local/ or normaliz*.mp. or normalis*.mp. [mp=title, original title, abstract, name of substance word, subject heading word]
46. 29 and 45
47. 46 not 44

WOS/SCOPUS
# 6 468 #5 OR #1
Databases=SCI-EXPANDED Timespan=1993-2009
# 5 311 #4 AND #3 AND #2
Databases=SCI-EXPANDED Timespan=1993-2009
# 4 >100,000 TS=(trial* or cohort* or random* or meta-analysis or metaanalys* or compar* or outcome*)
Databases=SCI-EXPANDED Timespan=1993-2009
# 3 4,433 TS=(prolactinoma* or hyperprolactin* or macroprolactin* or microprolactin* OR ((PRL or prolactin*) SAME (adenoma* or tumor* or tumour* or microadenoma* or macroadenoma*)))
Databases=SCI-EXPANDED Timespan=1993-2009
# 2 74,897 TS=(dihydroergocryptine or disulergine or docarpamine or dopamine or dopexamine or dronabinol or epinine or fenoldopam or glutamyldopa or ibopamine or indatraline or isomolpan or lisuride or mergocriptine or mesulergine or minaprine or moxiraprine or naxagolide or nolomirole or orotirelin or pardoprunox or pergolide or piribedil or pramipexole or preclamol or propylnorapomorphine or quinagolide or quinelorane or quinpirole or roxindole or sibenadet or sumanirole or talixpexole or terguride or vanoxerine)
Databases=SCI-EXPANDED Timespan=1993-2009
# 1 369 Topic=(prolactinoma* or hyperprolactin* or macroprolactin* or microprolactin* OR ((PRL or prolactin*) SAME (adenoma* or tumor* or tumour* or microadenoma* or macroadenoma*))) AND Topic=(((dopamine or dopaminergic) SAME (stimulat* or agonist*)) or (abeorphine or acetergamine or adrogolide or aletnamol or amantadine or apocodeine or apomorphine or aripiprazole or bifeprunox or bromocriptine or cabergoline or carmoxirole or ciladopa or dihydrexidine)) AND Topic=(trial* or cohort* or random* or meta-analysis or metaanalys* or compar* or outcome*)
Databases=SCI-EXPANDED Timespan=1993-2009

Prolactinoma - Natural History

1. hyperprolactinemia/ or prolactin/bl, se or hyperprolactin*.mp. [mp=title, original title, abstract, name of substance word, subject heading word]
2. 1 and ((prolactinom* or microprolatinom* or macroprolactinom*).mp. or exp adenoma/ or microadenoma*.mp. or macroadenoma*.mp. or exp pituitary neoplasms/) [mp=title, original title, abstract, name of substance word, subject heading word]
3. ..l/ 2 hu=y
4. exp clinical trials as topic/ or registries/ or interventional study/
5. longitudinal studies/ or case-control studies/ or exp cohort studies/ or observation*.mp. or prospective*.mp. [mp=title, original title, abstract, name of substance word, subject heading word]
6. follow-up studies/ or series.mp. or longterm*.mp. or baseline*.mp. [mp=title, original title, abstract, name of substance word, subject heading word]
7. limit 3 to (clinical trial, all or clinical trial, phase i or clinical trial, phase ii or clinical trial, phase iii or clinical trial, phase iv or clinical trial or comparative study or controlled clinical trial or evaluation studies or meta analysis or multicenter study or practice guideline or randomized controlled trial)
8. 3 and (4 or 5 or 6)
9. 7 or 8
10. exp adenoma/th, rt, su or exp pituitary neoplasms/th, su, rt or prolactinoma/th, rt, su or hyperprolactinemia/rt, th, su
11. exp radiotherapy/
12. exp Hypophysectomy/
13. exp Pituitary Gland/rt, th, de, su [Radiotherapy, Therapy, Drug Effects, Surgery]
14. 9 and (10 or 11 or 12 or 13)
15. 9 and (treatment outcomes/ or prognosis/ or disease progression/ or natural history/ or recurr*.mp. or treatment withdrawal.mp. or withdraw*.mp.) [mp=title, original title, abstract, name of substance word, subject heading word]
16. ((clinical or natural) adj2 (progress* or course* or history)).mp. [mp=title, original title, abstract, name of substance word, subject heading word]

17. (watchful* or monitor* or wait* or observ* or untreat* or nonoperat* or nontreat*).mp. [mp=title, original title, abstract, name of substance word, subject heading word]
18. 9 and (16 or 17)
19. (volume or size or growth or enlarge* or withdraw*).mp. [mp=title, original title, abstract, name of substance word, subject heading word]
20. exp Hypogonadism/
21. infertility/ or exp infertility, male/ or exp infertility, female/
22. exp Sexual Dysfunction, Physiological/
23. Galactorrhea/
24. exp bone diseases, metabolic/ or exp fractures, bone/ or exp heart valve diseases/
25. Amenorrhea/
26. Gynecomastia/
27. exp headaches/ or exp cranial nerve disorders/ or osteopenia.mp. [mp=title, original title, abstract, name of substance word, subject heading word]
28. exp Vision Disorders/
29. exp quality of life/
30. or/20-29
31. 14 or 15 or 18
32. exp Hypogonadism/co, pc or (infertility/co, pc or exp infertility, male/co, pc or exp infertility, female/co, pc) or exp Sexual Dysfunction, Physiological/co, pc or Galactorrhea/co, pc or (exp bone diseases, metabolic/co, pc or exp fractures, bone/co, pc or exp heart valve diseases/co, pc) or Amenorrhea/co, pc
33. 9 and 32
34. Amenorrhea/co, pc or Gynecomastia/co, pc or (exp headaches/co, pc or exp cranial nerve disorders/co or osteopenia/co, pc) or exp Vision Disorders/co, pc or exp quality of life/ or treatment outcomes/
35. 9 and 34
36. 31 or 33 or 35
37. 36 and (untreat* or nontreat* or sham or control* or nonoperat* or nonsurg* or wait*).mp. [mp=title, original title, abstract, name of substance word, subject heading word]
38. 36 and ((clinical or normal or natural) adj2 (course or progress* or history)).mp. [mp=title, original title, abstract, name of substance word, subject heading word]
39. 36 and placebo*.mp. [mp=title, original title, abstract, name of substance word, subject heading word]
40. 36 and watch*.mp. [mp=title, original title, abstract, name of substance word, subject heading word]
41. or/37-40
42. from 41 keep 1-140

SCOPUS
(TITLE-ABS-KEY(hyperprolactin* AND (hypersecret* OR prolactinom* OR macroprolactinom* OR microprolactin* OR adenoma* OR macroadenom* OR microadenoma*)) AND TITLE-ABS-KEY((untreat* OR "un-treated" OR "natural history" OR "normal course" OR progress*)))

WOS
Topic=(hyperprolactin* or prolactinoma* or macroprolactinoma* or microprolactinom* OR (prolactin secreting)) AND Topic=(((clinical or normal or natural) SAME (course or history or progress)) OR untreat* or "un-treat*")

COCHRANE
1 (hyperprolactin* or prolactinom* or microprolactin* or macroprolactin*).mp. [mp=title, short title, abstract, full text, keywords, caption text] 26 Advanced Display
2 ((clinical or normal or natural) adj2 (course or history or progress*)).mp. [mp=title, short title, abstract, full text, keywords, caption text] 580 Advanced Display
3 (untreat* or wait* or nontreat* or unoperat*).mp. [mp=title, short title, abstract, full text, keywords, caption text] 996 Advanced Display
4 1 and (2 or 3) 5 Advanced Display
5 1 and (arm*1 or control* or placebo* or sham).mp. [mp=title, short title, abstract, full text, keywords, caption text] 26
6 5 and (2 or 3) 5
7 4 or 6 5 Advanced Display
